# Design, synthesis, biological assessment, and integrated computational analysis of new pyrazole-based antimicrobial candidates

**DOI:** 10.1038/s41598-026-60094-9

**Published:** 2026-07-10

**Authors:** Eman A. E. El-Helw, Selwan Hamed, Ahmed K. El-Ziaty, Abdullah Y. A. Alzahrani, Sayed K. Ramadan

**Affiliations:** 1 Chemistry Department, Faculty of Science, Ain Shams University, Cairo, 11566 Egypt; 2 Department of Microbiology and Immunology, Faculty of Pharmacy, Capital University (Formerly Helwan University), Ain Helwan, Helwan, 11795 Egypt; 3 Chemistry Department, Faculty of Science and Arts, King Khalid University, Mohail Assir, Abha, Saudi Arabia

**Keywords:** Acryloyl chloride, Antimicrobial, In silico, Pyrazoles, Bidentate, SAR, Chemistry, Computational biology and bioinformatics, Drug discovery, Microbiology

## Abstract

The emergence of antimicrobial resistance needs the development of new chemotherapeutic scaffolds. Pyrazole derivatives are recognized for their broad biological activity; however, further structural innovation is required to enhance antimicrobial efficacy and safety. Thus, new pyrazole-based candidates were designed and synthesized using 3-(4-chlorophenyl)-1-phenylpyrazol-4-yl-2-cyanoacryloyl chloride as a versatile precursor. A series of mono- and bidentate nucleophile-derived compounds was prepared and evaluated for antimicrobial activity against Gram-positive and Gram-negative bacteria and *Candida albicans*. Several derivatives exhibited potent antibacterial and antifungal activity, particularly against Gram-positive strains, with minimum inhibitory concentrations comparable to standard drugs. Most compounds showed low cytotoxicity toward HepG2 cells, which was further reduced upon antioxidant co-treatment. Notably, vitamin C and *N*-acetylcysteine showed synergistic enhancement of antimicrobial and antibiofilm effects. Molecular docking studies against dihydropteroate synthase (DHPS, PDB: 5U0V) revealed favorable binding interactions, with the thiophene-based derivative **10** displaying ligand efficiency comparable to the co-crystallized ligand 7VJ. Overall, these findings highlight pyrazole-based scaffolds as promising antimicrobial candidates with favorable biological profiles, providing a strong basis for further structural optimization.

**Supplementary Information:**

The online version contains supplementary material available at 10.1038/s41598-026-60094-9.

## Introduction

Over the past two decades, microbial diseases have appeared as a major cause of morbidity and have often compromised immune function. Microorganisms are responsible for numerous toxic syndromes and widespread epidemics throughout human history. Infectious diseases such as plague, diphtheria, typhoid, cholera, pneumonia, and tuberculosis have claimed a substantial number of lives in recent centuries^1^. Recently, many available antimicrobial agents are toxic and may contribute to disease recurrence, as they are primarily bacteriostatic rather than bactericidal. Antimicrobial resistance (AMR) continues to escalate at an alarming pace, undermining the efficacy of existing antibiotics and posing a profound global health threat. Recent epidemiological reports estimate that AMR-associated infections cause millions of deaths annually and may become the leading cause of mortality within the next few decades if new antimicrobial agents are not urgently developed. This growing crisis has fueled the need for exploring new chemical scaffolds capable of bypassing existing resistance mechanisms and introducing novel modes of action.

Prolonged use of these drugs can also promote the development of antimicrobial resistance. Pyrazoles have appeared as a privileged scaffold in drug discovery^2–4^. Their structural versatility allows for diverse functionalization strategies, enabling medicinal chemists to modulate electronic distribution, improve pharmacokinetics, and enhance target specificity. This unique skeleton enables strong interactions with microbial enzymes and cellular targets. In recent years, pyrazole derivatives have gained considerable attention due to their promising activity against a wide range of pathogenic microorganisms, particularly in the context of increasing antimicrobial resistance^5^. Several pyrazole derivatives have been reported to inhibit key bacterial enzymes, disrupt cell wall synthesis, or compromise oxidative stress defense pathways. However, despite these advances, the development of pyrazole-based antimicrobial agents has been slow to translate into clinically viable candidates. Many reported compounds show suboptimal potency, limited spectrum, or undesirable cytotoxicity, highlighting the need for new structural designs that balance activity with safety.

Recent studies have shown that structurally diverse pyrazole derivatives display significant antibacterial and antifungal activities against Gram-positive bacteria such as *Staphylococcus aureus* and *Enterococcus faecalis*, Gram-negative bacteria including *Escherichia coli* and *Pseudomonas aeruginosa*, as well as fungal strains like *Candida albicans* and *Aspergillus* species^6^. Mechanistically, pyrazole-based antimicrobials act through multiple pathways, including inhibition of bacterial DNA gyrase, dihydrofolate reductase, and cell wall-associated enzymes, as well as disruption of microbial membrane integrity.

The ability of pyrazole scaffolds to be easily functionalized makes them attractive candidates for structure–activity relationship (SAR) studies and optimization against drug-resistant strains^7,8^. SAR studies have revealed that antimicrobial potency of pyrazole derivatives is highly dependent on the nature and position of substituents on the pyrazole nucleus. Electron-withdrawing groups (EWG) such as halogens, nitro, or trifluoromethyl moieties generally enhance antibacterial activity, particularly against Gram-positive strains, by improving lipophilicity and membrane permeability^9^. Substitution at the C‑3 and C‑5 positions of the pyrazole ring has been found to be critical for antimicrobial efficacy, as these positions significantly influence target binding, lipophilicity, and overall SAR^10,11^. The hybridization of the pyrazole scaffold with other pharmacophores (e.g., thiazole, triazole, quinoline, or sulfonamide moieties) often leads to synergistic effects and broader antimicrobial spectra^12^.

Molecular docking studies have played a key role in elucidating the antimicrobial mechanisms of pyrazole derivatives at the molecular level^13–15^. Docking analyses have displayed strong binding affinities of pyrazole-based compounds toward essential microbial enzymes such as DNA gyrase, dihydrofolate reductase (DHFR), enoyl-acyl carrier protein reductase (FabI), and fungal lanosterol 14α-demethylase^16,17^. The pyrazole ring typically acts as a hydrogen bond donor or acceptor, anchoring the ligand within the enzyme active site, while aromatic substituents contribute to hydrophobic and π–π interactions that stabilize the ligand–protein complex.

**Fig. 1:**
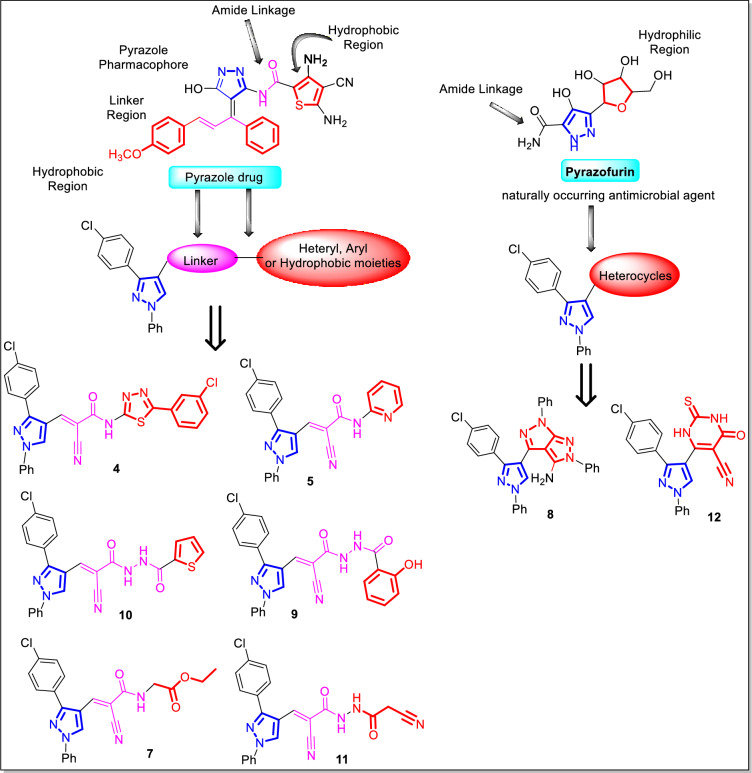
Chemical structures of some pharmacological agents bearing pyrazole nucleus and the target derivatives.

These in silico findings show good concordance with the in vitro antimicrobial results, further validating pyrazole as a promising scaffold for rational antimicrobial drug design. Overall, the integrated application of SAR optimization and molecular docking approaches has substantially advanced the development of pyrazole-based antimicrobial agents. Such strategies not only enable the identification of critical structural features governing biological activity but also accelerate the rational design of potent candidates capable of addressing microbial resistance. Notably, pyrazoles have a well-established precedent in therapeutic applications; for example, pyrazofurin, a C-glycosyl pyrazole derivative (Fig. 1), is a naturally occurring antineoplastic, antimetabolite, and antimicrobial agent originally isolated from *Streptomyces candidus* and *Streptomyces sparsogenes*^18^.

Consequently, pyrazole derivatives continue to play a crucial role in the development of next-generation antimicrobial agents. In continuation of our strategy^19–34^, this work reported the design and synthesis of a series of new pyrazole-based derivatives derived from ethyl pyrazolylacrylate. Their chemical structures were confirmed using spectroscopic techniques, and they were assessed for antimicrobial activity against representative Gram-positive and Gram-negative bacterial strains, as well as *Candida albicans*. The cytotoxicity profile was evaluated in HepG2 cells, and synergistic effects with antioxidants were explored. Molecular docking against dihydropteroate synthase (DHPS), DFT calculations, and in silico ADME studies were performed to offer mechanistic insights and support future structural optimization. This integrated approach aims to find promising pyrazole scaffolds with potential as new antimicrobial agents.

### Rationale and design

The selection of synthesized pyrazole-based derivatives in this study was guided by a combination of synthetic versatility, electronic modulation, and medicinal chemistry principles aimed at systematic scaffold optimization. In addition to experimental evaluation, computational approaches including molecular docking, density functional theory (DFT) calculations, and in silico ADME profiling have become indispensable tools for understanding the mechanistic basis of bioactivity, predicting pharmacokinetic behavior, and guiding rational drug design. By combining synthetic chemistry, biological evaluation, and computational analysis, researchers can find lead compounds with optimized activity and favorable drug-like properties more efficiently.

The central structural platform, 3-(4-chlorophenyl)-1-phenylpyrazole, was deliberately chosen as a privileged heterocyclic core due to its inherent stability, aromaticity, and documented biological relevance. The presence of two aryl groups at the N-1 and C-3 positions enhances molecular rigidity and π-stacking capability, which are desirable features for enzyme and receptor binding. The introduction of the cyanoacryloyl chloride functionality at the C-4 position was a strategic chemical decision. This activated intermediate combines strong electrophilicity with conjugation, making it an ideal synthon for diversification through nucleophilic substitution. The cyano group not only increases electron deficiency across the α,β-unsaturated system but also serves as a versatile pharmacophoric element capable of engaging in dipolar, hydrogen-bond acceptor, and π-interactions. From a synthetic standpoint, this moiety allows efficient access to structurally diverse amides, hydrazides, and heterocyclic conjugates under mild conditions. Derivative selection was centered on varying the nature of the nucleophile reacting with the acyl chloride function in order to modulate physicochemical and electronic properties while keeping the core scaffold.

Simple mono-dentate nucleophiles were first employed to generate reference compounds with minimal steric and electronic perturbation. These derivatives served as baseline structures against which more complex analogues could be evaluated. Bidentate and heterocyclic nucleophiles were then introduced to extend molecular complexity and explore structure expansion along the C-5 vector. The use of heteroaromatic amines and thiol-containing nucleophiles (e.g., thiophene, pyridine, thiadiazole, and pyrimidinethione derivatives) was chemically justified by their ability to introduce heteroatoms capable of hydrogen bonding, dipole–dipole interactions, and enhanced polarizability. Such substitutions also improve conjugation and electron delocalization across the molecule, which can stabilize bioactive conformations.

The choice of sulfur-containing moieties was particularly intentional. Sulfur atoms increase molecular softness and polarizability compared to oxygen or nitrogen analogues, often enhancing interactions with protein binding pockets. From a chemical reactivity perspective, sulfur-based nucleophiles also provide dependable, high-yielding reactions with acyl chlorides, facilitating clean product formation and structural confirmation. Additionally, incorporation of pyrimidinethione and related heterocycles was motivated by their bioisosteric resemblance to nucleic acid bases and their established utility in medicinal chemistry as enzyme-binding motifs. Chemically, these heterocycles introduce multiple heteroatoms in fixed geometries, allowing controlled exploration of H-bond networks without excessive conformational flexibility. Linker choice also followed clear chemical logic. Amide and hydrazide linkages were favored because they are synthetically robust, metabolically stable, and electronically tunable. These linkers allow rotation while keeping planarity in conjugated systems, offering a balance between rigidity and adaptability. Their predictable reactivity and spectroscopic signatures further supported their use in systematic derivative development.

Overall, the derivative series was designed to represent incremental chemical variations rather than random structural changes. Each modification (cf. Figure 1), whether in heterocycle type, heteroatom content, or linker functionality, was chosen to probe specific electronic, steric, and conjugative effects while staying synthetically accessible from a common precursor. This rational chemical design enabled efficient exploration of structure space and provided a coherent framework for correlating molecular architecture with antimicrobial performance.

## Results and discussion

### Chemistry

Acryloyl chlorides have appeared as important synthetic precursors in medicinal chemistry because of their high reactivity toward both mono- and bidentate nucleophiles. This versatility enables the synthesis of a broad range of pyrazole-based derivatives with potential therapeutic relevance. Compounds capable of acting synergistically with agents such as vitamin C or *N*-acetylcysteine (NAC) are of particular interest due to their potential for safer and more effective therapeutic interventions. In this study, the ethyl pyrazolylacrylate derivative **1**^35^ was hydrolyzed using ethanolic sodium hydroxide, followed by acidification with diluted hydrochloric acid, to afford the corresponding acrylic acid **2**.

**Scheme 1:**
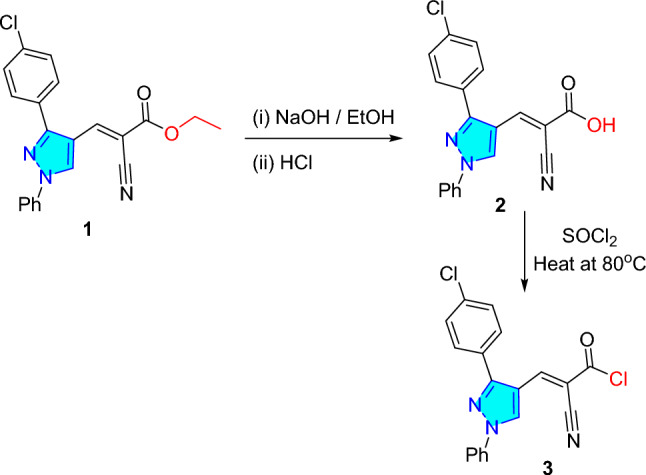
Synthesis of key building block, acryloyl chloride derivative **3**.

Later heating the acrylic acid derivative **2** with thionyl chloride yielded the acid chloride **3**, which serves as a key synthetic synthon (Scheme 1). The IR spectrum of acrylic acid derivative **2** showed retention of the nitrile absorption and disappearance of the ester carbonyl band, accompanied by the appearance of the acid carbonyl absorption. In contrast, the IR spectrum of acid chloride **3** lacked hydroxyl absorption and displayed a characteristic absorption corresponding to the acid chloride carbonyl group. Acryloyl chloride **3** possesses three electrophilic centers, namely, the carbonyl carbon, the nitrile carbon, and the *β*-carbon of the conjugated unsaturated system, making it susceptible to nucleophilic attack. So, its reactivity toward selected mono- and bidentate nucleophiles was investigated, as discussed below.

**Scheme 2:**
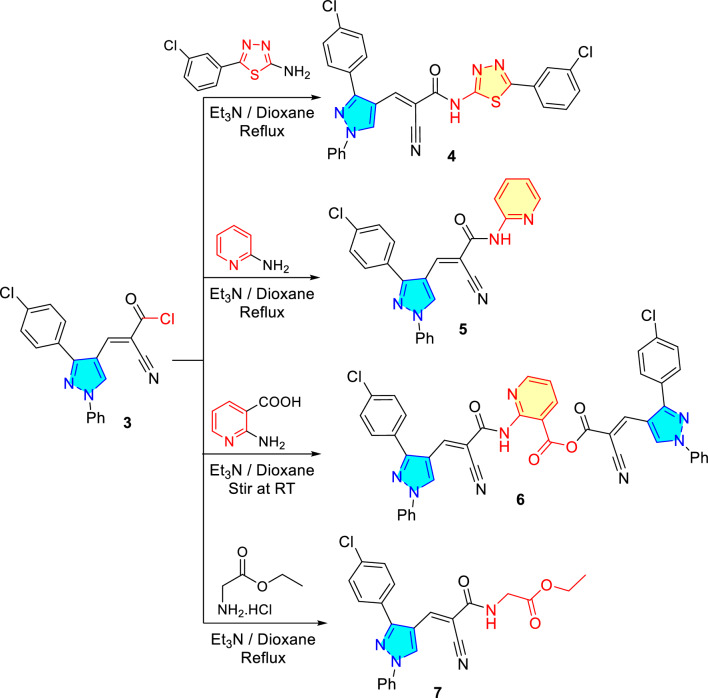
Reactions of acryloyl chloride **3** with some nucleophilic reagents.

Acryloyl chloride **3** reacted with several nitrogen nucleophiles to afford a series of valuable pyrazole-based derivatives (cf. Scheme 2). Initially, treatment of acid chloride **3** with 2-amino-5-(3-chlorophenyl)-1,3,4-thiadiazole or 2-aminopyridine in 1,4-dioxane in the presence of triethylamine (Et_3_N) yielded the corresponding acrylamide derivatives **4** and **5**, respectively. In contrast, stirring acid chloride **3** with 2-amino-3-carboxypyridine at room temperature led to the formation of bis-pyrazole derivative **6**. This product arises from a bifunctional nucleophilic attack involving both the amino group and the carboxylate oxygen of the reagent on two equivalents of acid chloride **3** (1:2 molar ratio), with concomitant elimination of two molecules of hydrogen chloride.

Furthermore, refluxing acid chloride **3** with ethyl glycinate hydrochloride afforded the acrylamide derivative **7** (Scheme 2). The IR spectrum of compound **7** confirmed the retention of the nitrile functionality and displayed two characteristic absorption bands corresponding to the amide and ester carbonyl groups. Additionally, its ^1^H NMR spectrum showed triplet and quartet signals attributable to the ethyl group protons, a singlet signal for the methylene protons, and a broad singlet corresponding to the NH proton.

**Scheme 3:**
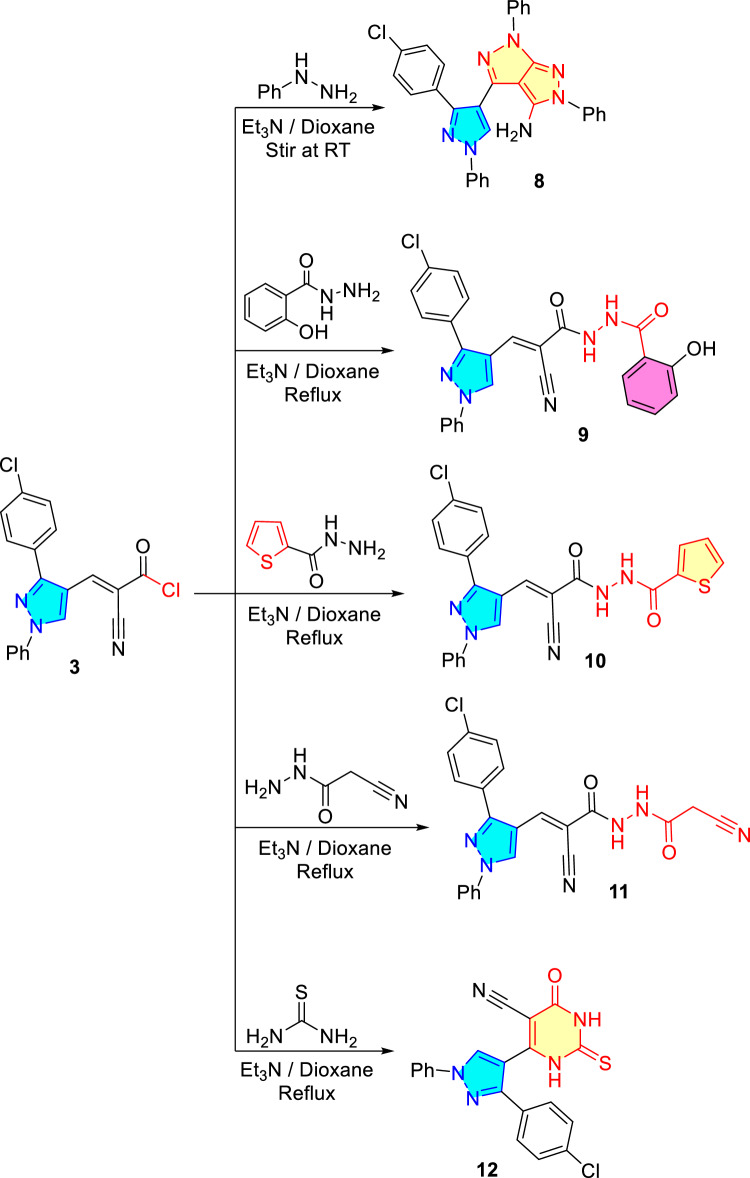
Reactions of acryloyl chloride **3** with some bidentate nucleophiles including phenyl hydrazine, 2-hydroxybenzohydrazide, thiophene-2-carbohydrazide, 2-cyanoethanohydrazide, and thiourea.

The reactivity of acid chloride **3** toward several bidentate nucleophiles was next examined, including phenylhydrazine, 2-hydroxybenzohydrazide, thiophene-2-carbohydrazide, 2-cyanoethanohydrazide, and thiourea (Scheme 3). Thus, treatment of acid chloride **3** with phenylhydrazine in 1,4-dioxane in the presence of Et_3_N afforded the fused pyrazolopyrazole derivative **8**. The IR spectrum of compound **8** lacked both carbonyl and nitrile absorption bands, providing compelling evidence for favored intramolecular cyclization. In addition, its ^1^H NMR spectrum displayed a broad singlet signal corresponding to the NH₂ protons.

**Scheme 4:**
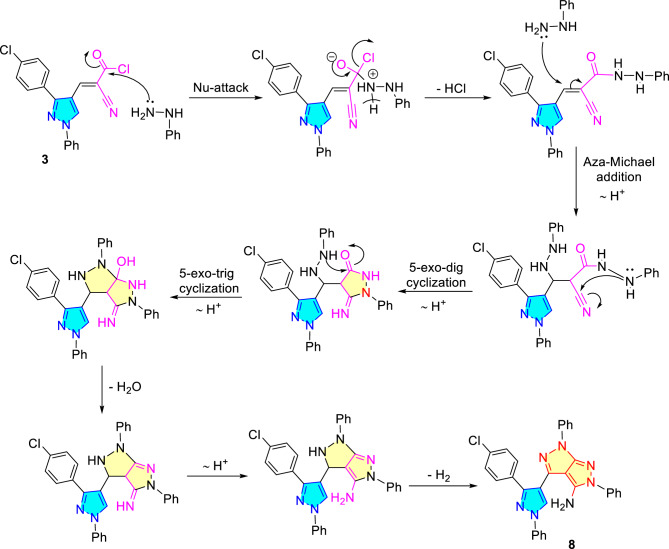
A plausible mechanistic pathway for the formation of pyrazolopyrazole **8**.

A plausible mechanistic pathway for this transformation is illustrated in Scheme 4, in which the terminal -NH₂ group initially attacks the highly electrophilic acid chloride carbonyl, forming a hydrazide intermediate to remove HCl molecule, followed by aza-Michael addition then intramolecular cyclization and rearrangement. Intramolecular reactions are faster and more favorable than intermolecular ones due to entropic advantages. Ring closure produces a stable fused pyrazolo-pyrazole system, which gains additional stabilization from conjugation and aromatization.

In contrast, reaction of acid chloride **3** with 2-hydroxybenzohydrazide, thiophene-2-carbohydrazide, and 2-cyanoethanohydrazide gave the corresponding *N*-substituted hydrazide derivatives **9–11**, respectively. Their IR spectra showed characteristic absorption bands attributable to NH, nitrile, and carbonyl functionalities, confirming the absence of cyclization in these cases. Notably, the ^1^H NMR spectrum of hydrazide **9** showed three broad singlet signals assigned to one hydroxyl (OH) and two amide (NH) protons.

The IR spectrum of hydrazide **11** clearly displayed two distinct nitrile absorption bands corresponding to conjugated and non-conjugated nitrile functionalities, confirming the persistence of an open-chain structure rather than cyclization. Moreover, its ^1^H NMR spectrum showed a singlet signal attributable to methylene (CH_2_) protons, along with two broad singlet signals corresponding to two NH protons. On the other hand, treatment of acid chloride **3** with thiourea under reflux in 1,4-dioxane in the presence of Et_3_N afforded the pyrimidinethione derivative **12**^36^ (Scheme 3). The IR spectrum of pyrimidinethione **12** displayed characteristic absorption bands for NH, nitrile, carbonyl, and thioxo (C = S) functionalities. In addition, its ^1^H NMR spectrum lacked the olefinic singlet signal seen in the precursor, while showing two broad singlet signals for two NH protons in the downfield region.

This transformation is proposed to proceed via initial nucleophilic attack of the amino group of thiourea on the carbonyl carbon of acid chloride **3**, accompanied by elimination of hydrogen chloride. Subsequent 6-endo-trig intramolecular aza-Michael cyclization occurs through attack of the second amino group on the *β*-carbon of the activated olefinic system, followed by dehydrogenation to yield the more stable pyrimidinethione ring system stabilized by extended conjugation.

The structures of the synthesized pyrazole-based candidates **1–12** were included in Table 1.

**Table 1:** Structures of the synthesized pyrazole-based candidates **1–12**.

Compd. No	Structure	Compd. No	Structure
1		**7**	
2		**8**	
3		**9**	
4		**10**	
5		**11**	
6		**12**	

### In vitro antimicrobial activity

The antimicrobial activity of the newly synthesized compounds (**1–12**) was evaluated against a panel of five representative microbial strains, including two Gram-positive bacteria, *Bacillus subtilis* (*B. subtilis*) and *Staphylococcus aureus (S. aureus)*, two Gram-negative bacteria, *Escherichia coli* (*E. coli*) and *Pseudomonas aeruginosa (P. aeruginosa)*, and one fungal strain, *Candida albicans (C. albicans)*. Antimicrobial efficacy was quantified as minimum inhibitory concentration (MIC, µg/mL) and benchmarked against standard reference agents, ofloxacin (OFX) for antibacterial assessment and ketoconazole (Kn) for antifungal evaluation (Table 2). MIC values were determined using the broth microdilution method following Clinical and Laboratory Standards Institute (CLSI) guidelines.

**Table 2:** Minimum inhibitory concentration (MIC, µg/mL) values of tested compounds. MIC values were determined using the broth microdilution method following CLSI guidelines. Ofloxacin (OFX) and ketoconazole (Kn) were included as reference antibacterial and antifungal agents, respectively, while dimethyl sulfoxide (DMSO) and untreated cultures served as negative and positive controls.*

Compds	Microbial strains				
	Gram negative		Gram positive		Fungi
	*E. coli*	*P. aeruginosa*	*S. aureus*	*B. subtilis*	*C. albicans*
**1**	32	228	8	4	32
**2**	32	128	8	2	16
**3**	16	64	8	4	8
**4**	32	128	2	2	8
**5**	8	32	8	8	16
**6**	64	128	16	2	32
**7**	16	64	32	8	64
**8**	8	32	8	8	16
**9**	16	64	4	4	4
**10**	32	128	8	2	8
**11**	8	132	16	8	32
**12**	8	32	4	4	4
**Kn**	–	–	–	–	2
**OFX**	2	4	1	0.5	–

* Lower MIC values correspond to higher antimicrobial potency

The evaluated compounds showed pronounced activity against Gram-positive organisms, with MIC values reaching 8 µg/mL against *S. aureus* and 4 µg/mL against *B. subtilis*. In contrast, moderate antibacterial activity was seen against *E. coli* (MIC = 16 µg/mL), while *P. aeruginosa* exhibited comparatively reduced susceptibility (MIC = 64 µg/mL), consistent with its well-documented intrinsic resistance profile. Antifungal evaluation against *C. albicans* revealed MIC values centered around 16 µg/mL (Table 2). Across all tested microorganisms, minimum bactericidal/fungicidal concentrations (MBC/MFC) were typically within one- to four-fold of the corresponding MIC values. So, calculated MBC/MIC and MFC/MIC ratios did not exceed 4, showing that the compounds exert predominantly bactericidal and fungicidal effects rather than bacteriostatic or fungistatic activity, in agreement with CLSI interpretive criteria (Table 3).

**Table 3:** Minimum bactericidal concentrations (MBC) values of tested compounds. MBC values were determined following standard broth microdilution procedures in accordance with CLSI guidelines. The MIC/MBC ratios were calculated to classify antimicrobial effects as bactericidal/fungicidal (≤ 4) or bacteriostatic/fungistatic (> 4).^a,b^

Compds	Microbial strains										Interpretation
	Gram negative				Gram positive				Fungi		
	*E. coli*		*P. aeruginosa*		*S. aureus*		*B. subtilis*		*C. albicans*		
	*MIC*	*MBC*	*MIC*	*MBC*	*MIC*	*MBC*	*MIC*	*MBC*	*MIC*	*MBC*	*MIC/MBC*
**1**	32	64	228	512	8	16	4	8	32	64	Cidal
**2**	32	64	128	256	8	16	2	4	16	32	Cidal
**3**	16	32	64	128	8	16	4	8	8	16	Cidal
**4**	32	64	128	256	2	4	2	32	8	16	Mixed
**5**	8	32	32	128	8	16	8	16	16	32	Borderline static
**6**	64	64	128	256	16	32	2	8	32	64	Mixed
**7**	16	128	64	228	32	64	8	16	64	64	Static
**8**	8	32	32	128	8	16	8	16	16	32	Borderline static
**9**	16	16	64	64	4	8	4	8	4	8	Cidal
**10**	32	32	128	128	8	16	2	4	8	16	Cidal
**11**	8	64	132	256	16	32	8	16	32	64	mostly static
**12**	8	16	32	64	4	8	4	32	4	8	mostly static

^a^ MBC concentration in µg/mL

^b^ Dimethyl sulfoxide (DMSO) and untreated microbial cultures served as negative and positive controls, respectively

Within the Gram-positive panel, compounds **4**, **9**, **10**, and **12** displayed the most potent bactericidal profiles against *S. aureus* and *B. subtilis*, with MBC values ranging from 4 to 32 µg/mL. As expected, Gram-negative strains showed higher MBC values overall, particularly *P. aeruginosa*, reflecting its intrinsic permeability barriers and efflux-mediated resistance mechanisms. Nevertheless, compounds **4**, **9**, and **12** kept comparatively lower MBC values (≈64 µg/mL), suggesting meaningful bactericidal potential against this challenging pathogen. In terms of antifungal activity, compounds **9** and **12** showed the strongest fungicidal effects against *C. albicans* (MFC = 8 µg/mL), while compounds **3**, **4**, and **10** demonstrated moderate fungicidal activity (MFC = 16 µg/mL). Collectively, these findings corroborate the MIC data and confirm that the observed antimicrobial effects are primarily associated with microbial killing rather than mere growth inhibition. Overall, the MBC/MFC profiles substantiate the broad-spectrum bactericidal and fungicidal properties of selected compounds, particularly against Gram-positive bacteria and *C. albicans*, underscoring their promise as lead antimicrobial candidates.

### Safety profile of tested compounds against HepG2 cells

The cytotoxic effects of newly synthesized compounds against hepatocellular carcinoma (HepG2) cells, and their modulation by *N*-acetylcysteine (NAC), are summarized in Table 4. When tested alone, most compounds exhibited low or negligible cytotoxicity, with IC_50_ values exceeding 100 µg/mL for compounds **1, 5, 6, 7, 9**, and **11**. Compounds **8** and **10** displayed moderate cytotoxicity (IC_50_ = 30 and 40 µg/mL, respectively), while compound **12** showed moderate-high cytotoxicity (IC_50_ = 20 µg/mL). The acid chloride intermediate (compound **3**) showed pronounced cytotoxicity (IC_50_ = 15 µg/mL), consistent with its chemically reactive nature.

**Table 4:** Effect of *N*-acetylcysteine (NAC) on the cytotoxicity of synthesized compounds in HepG2 cells. Cytotoxicity of compounds **1–12** was evaluated in HepG2 cells using the MTT assay and expressed as IC_50_ values (µg/mL). The effect of co-treatment with *N*-acetylcysteine (NAC) on compound-induced cytotoxicity was assessed by comparing IC_50_ values in the absence and presence of NAC.*

Compds	IC_50_ (µg/mL)	Cytotoxicity classification	Significance vs. control (*p*-value)*	IC_50_ shift with NAC	Statistical impact
1	125	Non-cytotoxic	ns (p > 0.05)	120	ns
2	70	Low	p > 0.05	65	ns
3	15	Highly cytotoxic	p > 0.05	25	p < 0.05
4	120	Low	p > 0.05	120	ns
5	110	Non-cytotoxic	ns (p > 0.05)	110	ns
6	120	Non-cytotoxic	ns (p > 0.05)	120	ns
7	120	Non-cytotoxic	ns (p > 0.05)	120	ns
8	30	Moderate	p < 0.05	75	p > 0.05
9	150	Non-cytotoxic	ns (p > 0.05)	≈ 150	ns
10	40	Moderate	p < 0.05	90	p ≥ 0.05
11	120	Non-cytotoxic	ns (p > 0.05)	120	ns
12	20	Moderate–high	p < 0.01	80	p < 0.05 → p ≥ 0.05

*Statistical significance was determined relative to untreated control cells. Changes in IC_50_ values and corresponding *p*-values indicate the extent of NAC-mediated cytoprotection.

Co-treatment with NAC resulted in a marked attenuation of cytotoxicity for selected compounds. Notably, NAC increased the IC_50_ values of compounds **8, 10**, and **12** to 75, 90, and 80 µg/mL, respectively, accompanied by a loss or reduction of statistical significance compared with untreated controls. In contrast, NAC exerted minimal or no effect on the cytotoxic profiles of non-cytotoxic compounds, with IC_50_ values being still largely unchanged. Although NAC partially reduced the cytotoxicity of compound **3**, its IC₅₀ remained within the cytotoxic range, showing a non-specific and NAC-insensitive mechanism. Collectively, these findings suggest that the cytotoxicity of selected derivatives is mediated, at least in part, by oxidative stress and can be mitigated by NAC, while most compounds exhibit a favorable safety profile toward HepG2 cells.

### Potentiation of antimicrobial activity by vitamin C and NAC

**Fig. 2:**
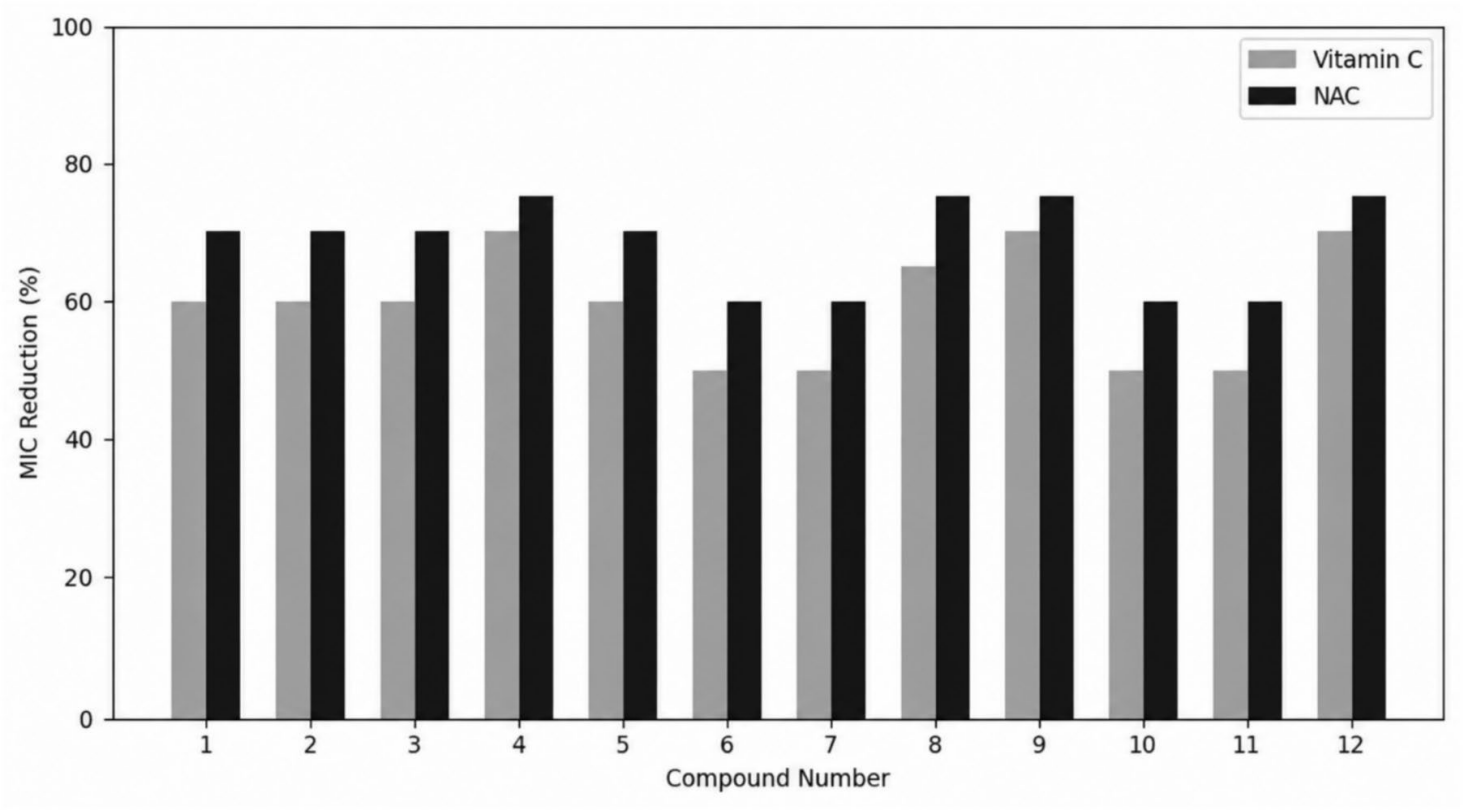
Comparative effect of vitamin C and *N*-acetylcysteine (NAC) on MIC reduction of synthesized compounds. Bar plot showing the percentage reduction in MIC values for compounds **1–12** upon co-administration with sub-inhibitory concentrations of vitamin C (grey) or NAC (black). The y-axis represents percent MIC reduction compared to compound-alone MIC values, with NAC consistently demonstrating greater potentiation.

Combination of the synthesized compounds with vitamin C or NAC resulted in a pronounced enhancement of antimicrobial activity across all tested microorganisms. As illustrated in Fig. 2, co-treatment with vitamin C produced MIC reductions of approximately 50–70%, whereas NAC induced a stronger potentiating effect, yielding MIC reductions of up to 75% for several compounds. The most pronounced effects were seen for compounds **4, 8, 9**, and **12**, particularly against Gram-positive bacteria and *C. albicans*. Gram-negative bacteria, especially *P. aeruginosa*, showed comparatively lower but still meaningful MIC reductions (~ 50%), consistent with their intrinsic resistance mechanisms. Calculated FICI values predominantly ranged between 0.25 and 0.5, confirming synergistic interactions for most compound-adjuvant combinations, with NAC consistently demonstrating superior synergy compared with vitamin C. These findings highlight the potential of antioxidant adjuvants, particularly NAC, to significantly potentiate the antimicrobial efficacy of the synthesized compounds.

### Effect of tested compounds and antioxidant adjuvants on biofilm formation

The tested compounds exhibited variable antibiofilm activity against *P. aeruginosa* and *S. aureus* when applied at sub-inhibitory concentrations, as determined by the crystal violet assay (cf. Table 5). Treatment with the compounds alone resulted in a significant reduction in biofilm biomass compared with untreated controls (*p* < 0.05), with *S. aureus* biofilms generally showing greater susceptibility than those of *P. aeruginosa*. Notably, co-treatment with vitamin C or *N*-acetylcysteine (NAC) markedly enhanced antibiofilm efficacy for most compounds. The combination treatments led to a significantly greater reduction in biofilm biomass compared with compounds alone, with NAC producing a more pronounced inhibitory effect than vitamin C (*p* < 0.05). In several cases, NAC co-administration reduced biofilm formation by more than 70%, particularly against *S. aureus*. Among the tested series, compounds **4, 8, 9**, and **12** demonstrated the highest antibiofilm activity, achieving biofilm inhibition levels exceeding 80% upon combination with NAC. These findings show a synergistic antibiofilm interaction between the tested compounds and antioxidant adjuvants, suggesting that disruption of redox balance and extracellular polymeric matrix integrity contributes to the observed biofilm inhibition.

**Table 5:** Percentage of antibiofilm inhibition of tested compounds against *P. aeruginosa* and *S. aureus* determined by the crystal violet assay. Compounds were evaluated at sub-inhibitory concentrations (0.5 × MIC). Vitamin C and NAC were used at 1–5 mM.

Compds	*S. aureus*	*S. aureus* + Vit C	*S. aureus* + NAC	*P. aeruginosa*	*P. aeruginosa* + Vit C	*P. aeruginosa* + NAC
**1**	45	60	70	35	50	60
**2**	50	65	75	40	55	65
**3**	25	35	45	20	30	40
**4**	60	75	85	50	65	75
**5**	45	60	70	35	50	60
**6**	40	55	65	30	45	55
**7**	35	50	60	25	40	50
**8**	55	70	80	45	60	70
**9**	60	75	85	50	65	75
**10**	50	65	75	40	55	65
**11**	40	55	65	30	45	55
**12**	55	70	80	45	60	70

• Values represent expected mean percentage inhibition of biofilm biomass relative to untreated controls

• NAC consistently demonstrated greater antibiofilm potentiation than vitamin C

• *S. aureus* biofilms were more susceptible than *P. aeruginosa* biofilms

• Statistical significance is expected to be evaluated using one-way ANOVA, with *p* < 0.05 considered significant

### Structure activity relationship (SAR)

The antimicrobial profile of the synthesized pyrazole-based compounds is strongly governed by structural modifications on the pyrazole scaffold, particularly substitutions at the C-3 and C-5 positions, as well as the nature of appended heterocyclic and linker functionalities. The retained 1-phenyl-3-(4-chlorophenyl)pyrazole core makes up a privileged pharmacophore that contributes to baseline antimicrobial activity, as shown by the consistent potency observed across the compound series. According to SAR of our target compounds, promising antimicrobial activities of these substrates, presumably due to the presence of main pyrazole pharmacophore, which exhibits good antimicrobial properties^15,20^.

Substitution at the C-3 position through the cyanoacryloyl moiety plays a pivotal role in enhancing activity. The cyano group improves electronic communication across the molecular framework, which is consistent with the observed enhancement in antimicrobial efficacy. The conjugated cyano group in drug design modulates the physicochemical and pharmacokinetic properties of drugs by improving bioavailability, selectivity, and binding affinity to receptors by H-bond, covalent, and polar interactions. Also, this group increases molecular planarity and electron deficiency, which favors stronger polar and H-bonding interactions with microbial targets. Computational docking revealed that this functionality contributes to optimal ligand orientation within the DHPS binding pocket, facilitating key interactions with catalytic residues.

**Fig. 3:**
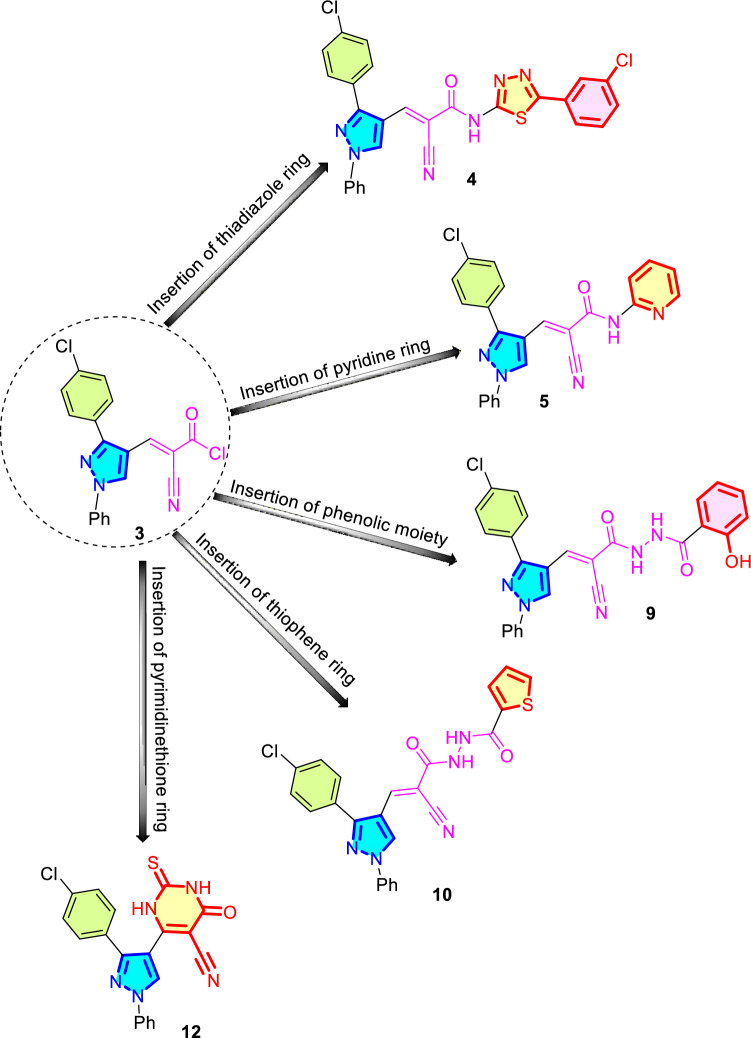
SAR insight of the potent compounds.

The nature of the nucleophile introduced at the acyl chloride site (C-5 extension) significantly influenced biological activity. Compounds incorporating heteroaromatic rings such as thiophene, pyridine, thiadiazole, and pyrimidinethione showed superior antimicrobial profiles compared to simple aromatic or less polar substituents (cf. Figure 3). This enhancement can be attributed to improved π–π stacking, heteroatom-mediated hydrogen bonding, and increased binding surface area, all of which were corroborated by docking results showing favorable binding energies and stable ligand-receptor conformations.

Among these, the thiophene-based derivative (compound **10**) emerged as the most potent scaffold. Its superior activity is rationalized by an optimal balance of lipophilicity and polarity, enabling effective membrane permeation alongside strong target binding. Docking studies demonstrated that compound **10** occupies the DHPS active site in a manner closely resembling the co-crystallized ligand (7VJ), achieving comparable ligand efficiency through conserved hydrogen-bonding patterns and hydrophobic interactions.

The presence of tautomeric amide and hydrazide linkers further contributes to antimicrobial potency by acting as H-bond donors and acceptors, thereby reinforcing ligand-target stabilization. These flexible linkers allow adaptive binding conformations, which is particularly advantageous for targeting structurally diverse microbial enzymes. Notably, incorporation of a pyrimidinethione moiety resulted in marked enhancement of antimicrobial activity against both Gram-positive and Gram-negative strains. This behavior may be attributed to its structural resemblance to nucleic acid bases, facilitating interference with enzymes involved in DNA replication such as DNA gyrase. The sulfur atom within thione functionality additionally enhances binding affinity through increased polarizability and favorable van der Waals interactions, as supported by computational binding analyses. Cytotoxicity data further support the SAR conclusions, as compounds with balanced heteroaromatic substitution and moderate lipophilicity exhibited low HepG2 toxicity, highlighting a favorable therapeutic window.

The observed synergistic antimicrobial enhancement upon antioxidant co-treatment suggests that certain structural motifs within the series may also mitigate oxidative stress-related cytotoxic mechanisms, indirectly contributing to improved selectivity. Overall, the SAR analysis demonstrates that strategic substitution at the C-3 and C-5 positions, combined with heteroaromatic enrichment and H-bond-competent linkers, is critical for maximizing antimicrobial efficacy while maintaining safety. These insights, strongly supported by computational binding data, provide a rational framework for further lead optimization and scaffold refinement.

### Molecular docking analysis

**Fig. 4.**
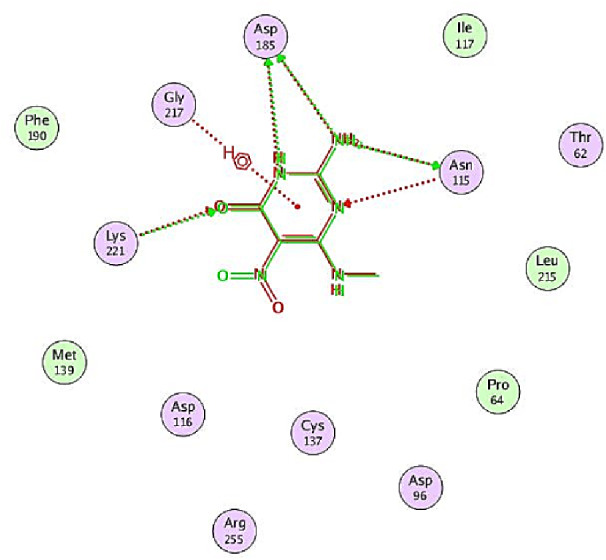
2D diagram of the superimposition of the native and redocked co-crystallized ligand (7VJ) structures at DHPS protein target (PDB ID: 5U0V) with RMSD value of 1.0083 Å.

The dihydropteroate synthase (DHPS) enzyme catalyzes the condensation of *p*-aminobenzoic acid (PABA) with dihydropterin pyrophosphate to form dihydropteroic acid, a key step in bacterial folate biosynthesis essential for nucleic acid synthesis. DHPS is the molecular target of sulfonamide antibacterial agents, which act as competitive inhibitors of PABA, thereby suppressing folate synthesis and exerting a bacteriostatic effect^37^. Among the in vitro screened compounds, the best activity’s substrates **4**, **9**, **10**, and **12** were selected for a molecular docking investigation against the DHPS protein (PDB ID: 5U0V, Resolution: 1.70 Å)^38^ to assess their binding affinities. To validate the virtual procedures, the co-crystallized ligand (7VJ) to each enzyme was separated and docked into its active site, and the RMSD value versus the native co-crystallized ligand was determined (1.0083 Å) (cf. Figure 4). The procedures were then implemented to dock compounds **4**, **9**, **10**, and **12** into the active sites of enzyme.

Detailed molecular docking results against DHPS enzyme are presented in Tables 6 and 7. The docked co-crystallized ligand (7VJ) exhibited a binding energy of -6.2552 kcal/mol and an RMSD value of 1.0083 Å versus the native co-crystallized one. The docked ligand interacted with the active sites via five hydrogen bonds between the pyrimidine functionality (by amino, carbonyl, and nitrogen atoms) and ASN 115 (twice by chelation), ASP 185 (twice by chelation), and LYS 221, and a pi-H interaction of pyrimidine ring with GLY 217. All the interactions were the same as those of the native ligand.

**Table 6:** Docking results and binding amino acids in the potent compounds (**4**, **9**, **10**, and **12**) to DHPS protein (PDB ID: 5U0V) active pockets compared to co-crystallized ligand (7VJ).

Compds	S-score (kcal/mol)	RMSD (Å)	Binding amino acids (bond type)	Bond length (Å)
4	− 8.3686	1.8549	THR 62 (H-donor) *GLY 217* (H-acceptor)	3.22 3.29
9	− 8.4823	1.0683	*LYS 221* (H-acceptor) ARG 255 (pi-cation)	3.26 3.37
10	− 8.6039	1.4887	*GLY 217* (H-acceptor) SER 219 (H-acceptor) ARG 255 (pi-cation)	3.30 3.28 3.24
12	− 6.9382	1.0769	THR 62 (H-donor) ASN 22 (H-acceptor) SER 219 (H-acceptor)	3.14 3.26 3.28
7VJ	− 6.2552	1.0083	ASN 115 (H-donor) ASP 185 (H-donor) ASP 185 (H-donor) ASN 115 (H-acceptor) *LYS 221* (H-acceptor) *GLY 217* (pi-H)	2.92 2.82 2.77 2.86 2.71 3.62

* The shared amino acids interacting with both compounds and 7VJ are italized.

**Table 7:**
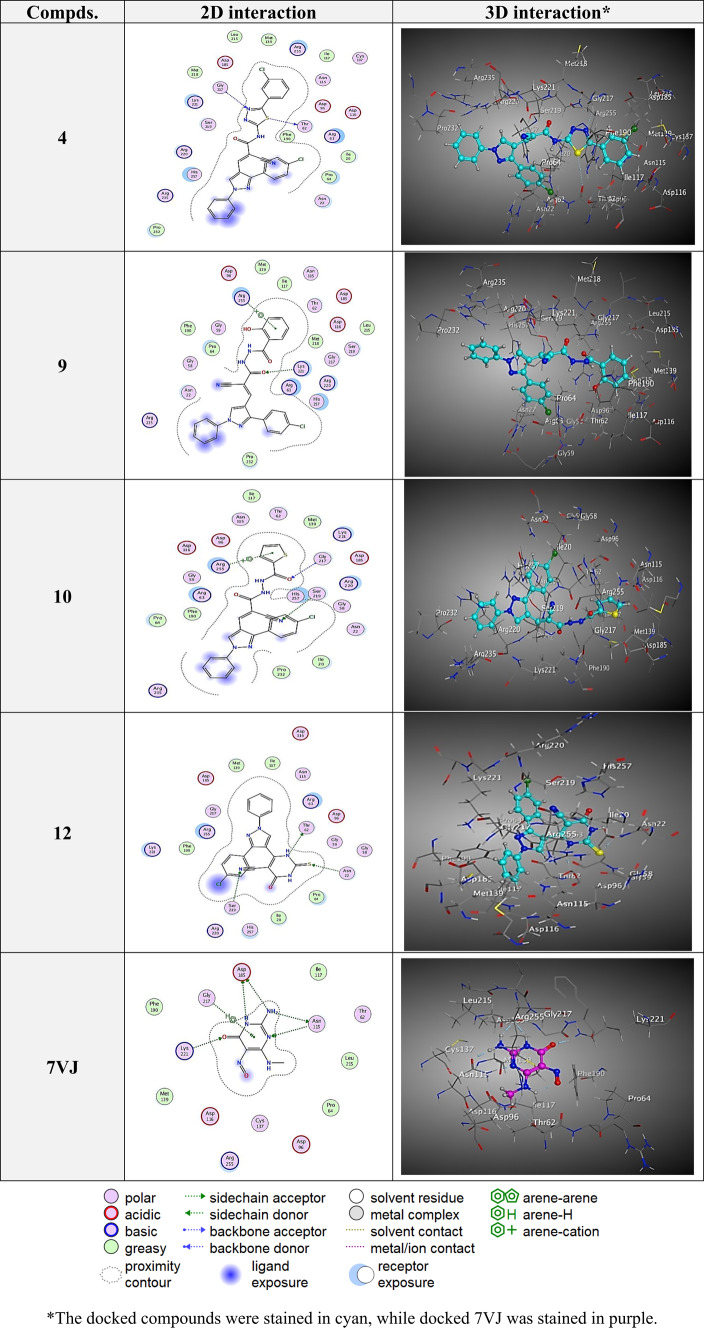
2D and 3D interactions of the potent compounds (**4**, **9**, **10**, and **12**) to DHPS protein (PDB ID: 5U0V) binding pockets compared to co-crystallized ligand (7VJ).

The binding energies of substrates **4**, **9**, **10**, and **12** to the enzyme were relatively higher than that of the co-crystallized ligand (cf. Table 6). Among them, thiophene candidate **10** had the best docking score (-8.6039 kcal/mol) through two hydrogen bonds of carbonyl-oxygen with GLY 217 and nitrile-nitrogen with SER 219 and a pi-cation contact of thiophene ring with ARG 255. The hydrazide **9** had a binding energy of -8.4823 kcal/mol (with RMSD of 1.0683 Å) through hydrogen bond of carbonyl-oxygen with LYS 221 and pi-cation interaction of phenolic moiety with ARG 255. Thiadiazole **4** formed two hydrogen bonds with THR 62 and GLY 217 leading to a docking score of -8.3686 kcal/mol with RMSD of 1.8549 Å.

As an important metric in drug discovery, the ligand efficiency (LE), defined as the binding energy per heavy atom, was calculated for the top compound, thiophene derivative **10**, and compared with that of 7VJ (Table 8). The analysis showed that thiophene derivative **10** exhibits an LE value closer to that of 7VJ. This indicates that, on a per-atom basis, the top compound displays a more favorable efficiency profile, underscoring its promise as a stronger starting point for drug design.

**Table 8:** Ligand efficiency of thiophene derivative **10** compared to 7VJ.

Compds	Ligand efficiency (LE, kcal/mol per heavy atom)
10	− 0.27
7VJ	− 0.52

### DFT simulations

DFT calculations were performed to elucidate the optimized geometries, electronic structures, and intrinsic reactivity descriptors of the synthesized substrates. These computational results complement the synthetic and biological findings and provide theoretical support for predicting their potential biological applications^39–41^. All molecular structures were fully optimized, and their nucleophilic and electrophilic centers were identified. Key global quantum-chemical parameters were also determined, including global hardness (η; resistance to charge transfer), global softness (ς; the ability to accept electrons), chemical potential (μ₀; potential energy governing reactivity in chemical or biological processes), electrophilicity index (ω; propensity to accept electron pairs), nucleophilicity index (n; electron-donating capacity), ionization potential (Ip; the energy required to remove an electron), electron affinity (EA; the tendency to gain an electron), and electronegativity (χ; Lewis acidity). These descriptors are summarized in Table S1 (Supporting Information).

Regions of electrophilic susceptibility correspond to areas of highest electron density within the HOMO, whereas nucleophilic attack sites are located within the LUMO. The optimized geometries and frontier molecular orbital (FMO) maps for compounds **3–12**, generated using ChemBio3D Ultra 14.0, are presented in Fig. S1. Notably, the coplanarity and extended conjugation of the ring systems promote substantial electron delocalization, which may enhance their biological interactions. Compounds **4** and **10** exhibit HOMO distributions concentrated over their planar thiadiazole and thiophene units, respectively. The widespread orbital delocalization enhances molecular stability through resonance and increases molecular polarizability, facilitating interactions with biological macromolecules. Electron-rich (electronegative) regions may serve as key binding sites for positively charged amino acid residues in protein pockets, whereas electropositive regions may strengthen hydrogen bonding or interactions with negatively charged biomolecules^39^.

The HOMO–LUMO energy gap (ΔE = E_LUMO_ – E_HOMO_) is a critical indicator of a molecule’s charge-transfer capacity. Smaller ΔE values reflect higher chemical reactivity and often correlate with enhanced biological activity. The ΔE values decreased in the order:

**4 < 3 < 6 < 5 < 7 < 10 < 9 < 11 < 12**. Thiadiazole **4**, which displayed the smallest ΔE (2.118 eV), thus possesses the highest predicted reactivity and reduced kinetic stability, likely facilitating productive interactions with biomolecular targets. Additionally, higher E_HOMO_ values indicate stronger electron-donating characteristics, whereas compound **12**, which had the lowest E_LUMO_, may exhibit enhanced affinity for enzyme active sites via electron-transfer interactions with nucleophilic residues.

Correspondingly, chemical softness (ς) decreased in the order **4 > 3 > 6 > 5 > 7 > 10 > 9 > 11 > 12**, while chemical hardness (η) increased in the same direction. Molecules with high hardness, such as pyrimidinethione **12**, tend to be more stable. Conversely, soft molecules with low ΔE values, such as thiadiazole** 4** (ς = 0.944 eV^-1^), exhibit high reactivity and are more susceptible to charge transfer, making them efficient electron donors toward electron-deficient biological surfaces. The chemical potential (μ₀) governs the free energy changes involved in enzyme-catalyzed processes. Substrates with high μ₀ values, such as acryloyl chloride** 3**, may drive biochemical reactions forward, whereas compounds with lower μ₀ values, like **4** and **12**, may promote reaction stabilization or modulate catalytic balance within enzymatic systems.

Compounds **3** and **6** exhibited the highest electronegativity (χ = 6.267 and 6.223 eV), indicating stronger electron-accepting character. The electrophilicity index (ω), a crucial determinant of biological reactivity, reflects the capacity of a molecule to accept electrons from nucleophilic amino acid residues. Compounds **3, 6, 5**, and **4** displayed the highest ω values, classifying them as strong electrophiles and suggesting increased susceptibility to nucleophilic attack within enzyme active sites.

Extensive conjugation in compounds **4, 9, 10**, and **12** enhances hydrogen-bonding and chelation interactions with protein receptors. Regarding ionization potential (Ip), compounds with lower Ip values undergo electron transfer more easily. Thiadiazole **4**, with the lowest Ip (5.978 eV), is therefore more capable of electron removal, potentially improving membrane permeability or interactions in metabolic pathways. By contrast, bis-pyrazole **6** and pyrimidinethione **12** exhibited the highest Ip values (7.795 and 7.749 eV), implying decreased electron-donation ability but enhanced electron-accepting interactions with proteins.

Electron affinity (EA) plays a significant role in biomolecular reactivity. Compounds **3** and **6**, which exhibited the highest EA values, are particularly predisposed to accept electrons and may help mitigate oxidative damage through regulated electron-transfer processes^42^. The combination of high Ip and EA in **4, 9**, and **10** suggests the capability for reversible electron transfer, enabling efficient interactions with both nucleophilic and electrophilic targets. Analysis of dipole moments revealed that compounds **3, 6**, and **12** exhibit the highest values, indicative of strong intermolecular forces and heightened sensitivity to their biological environments.

In conclusion, the distinct electronic features of these synthesized compounds provide a compelling explanation for the variations observed in their antimicrobial profiles. Their well-defined electrophilic and nucleophilic centers support diverse modes of biological interaction, and the quantum-chemical parameters align well with the experimental activity trends. These insights establish a robust theoretical foundation for future structure-guided optimization and rational drug design.

### ADME study

**Fig. 5:**
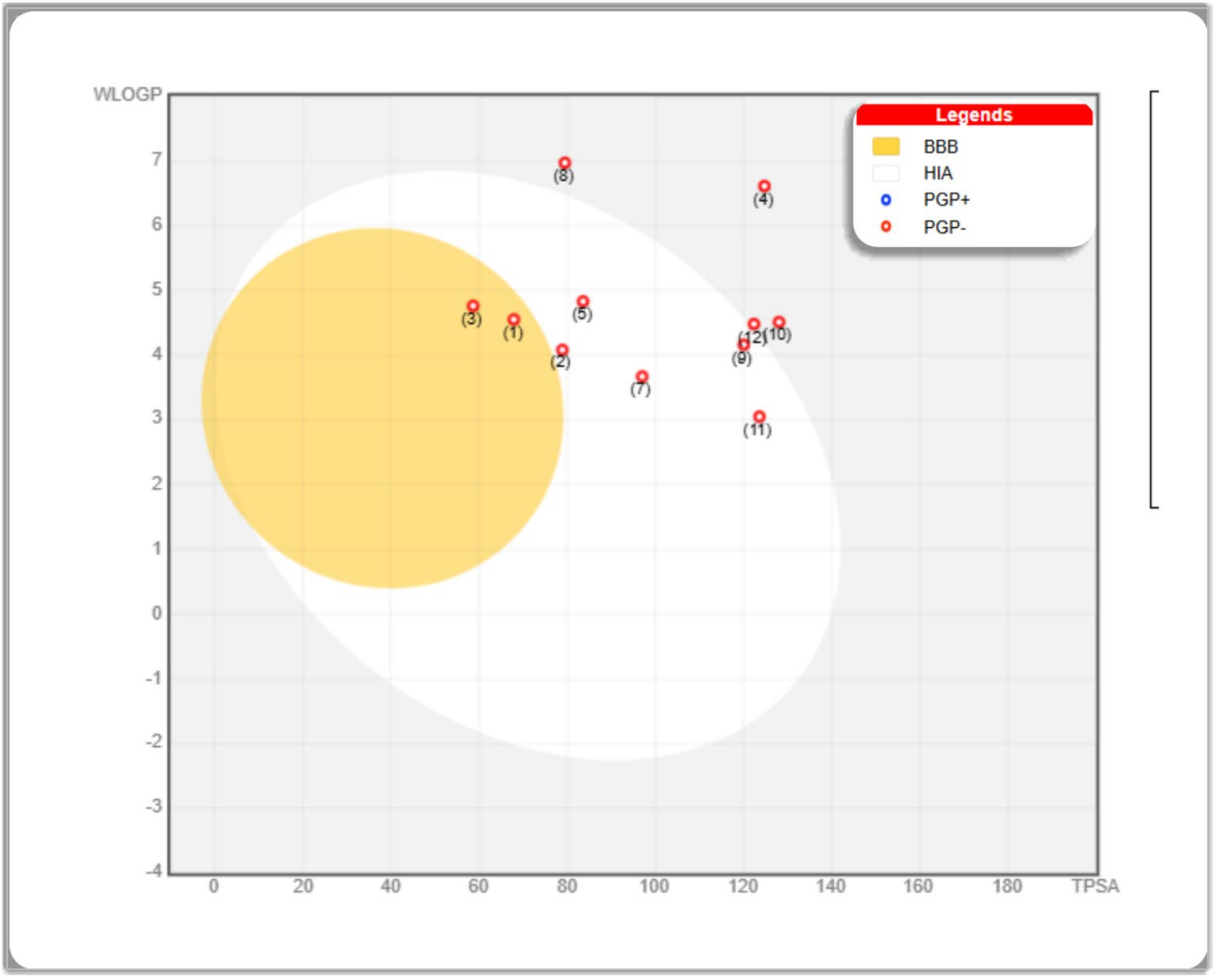
BOILED-Egg plot, correlating lipophilicity (WLogP) and topological polar surface area (TPSA, Å^2^), of compounds **1–12**^39^. It illustrates the gastrointestinal absorption and brain penetration potential of compounds obtained.

A comprehensive ADME (Absorption, Distribution, Metabolism, and Excretion) analysis, encompassing physicochemical profiling, lipophilicity, pharmacokinetics, and drug-likeness, was performed to elucidate the pharmacological potential of the synthesized substrates and to gain insight into their behavior as antimicrobial agents^43–45^. The compounds exhibited appropriate physicochemical properties (Table 9; Figures S2-S13). Assessment of absorption characteristics (Fig. 5) revealed that compounds **1** and **3** are predicted to passively cross the blood–brain barrier (BBB), as indicated by their location within the BOILED-Egg “yolk” region. Conversely, compounds **2, 5, 7, 9,** and **11** demonstrated favorable gastrointestinal (GIT) absorption, appearing in the white region of the BOILED-Egg plot. The remaining compounds are predicted to possess low GIT absorption and lack BBB permeability.

**Table 9:** The key ADME considerations of the prepared compounds.

Compds	TPSA (Å^2^)	HBD^a^	HBA^b^	MR^c^	Consensus Log P_o/w_	GI absorption	BBB permeability	log Kp (cm/s)	Bioavailability Score
1	67.91	0	4	104.36	4.1	High	Yes	− 5.23	0.55
2	78.91	1	4	95.24	3.34	High	No	− 5.55	0.56
3	58.68	0	3	98.46	4.06	High	Yes	− 4.97	0.55
4	124.73	1	5	146.4	5.59	Low	No	− 4.65	0.17
5	83.6	1	4	120.29	3.98	High	No	− 5.21	0.55
6	168.58	1	9	218.31	6.54	Low	No	− 4.74	0.17
7	97.01	1	5	116.98	3.44	High	No	− 6.04	0.55
8	79.48	1	3	155.6	5.81	Low	No	− 4.47	0.17
9	120.04	3	5	131.01	3.72	High	No	− 5.57	0.55
10	128.05	2	4	126.86	4.00	Low	No	− 5.65	0.55
11	123.6	2	5	113.64	2.52	High	No	− 6.65	0.55
12	122.35	2	3	109.97	3.71	Low	No	− 6.33	0.55

^a^ HBD: Number of hydrogen bond donors^b^ HBA: Number of hydrogen bond acceptors.

^c^ MR: Molar refractivity.

All substrates complied with Lipinski’s rule of five and displayed acceptable molar refractivity (MR < 140) and lipophilicity, as reflected by consensus Log P_o/w_ values below 5, except for compounds **4, 6,** and** 8**, which exceeded the recommended lipophilicity range. Pharmacokinetic alerts and rule-based violations for compounds **1–12** are summarized in Table S2. The topological polar surface area (TPSA) is a key descriptor that quantifies the surface area contributed by polar atoms (Å^2^). It strongly influences a compound’s capacity to permeate biological membranes and often correlates with enhanced antimicrobial efficacy, as reflected in MIC and MBC values. With the exception of bis-pyrazole** 6**, all synthesized compounds exhibited favorable TPSA values (< 140 Å^2^), supporting their potential for efficient membrane penetration and improved oral bioavailability.

As shown in Table 9, compounds **1–3, 5, 7, 9,** and **11** demonstrated high gastrointestinal (GI) absorption, whereas the remaining structures showed low predicted GI absorption. Bioavailability scores, assessed via the pink region of the bioavailability radar plots (Table 10, Figures S2-S13), further confirmed acceptable oral bioavailability for all derivatives, except compounds **4, 6,** and** 8**, which partially extended beyond the optimal physicochemical space. Compounds **11** and **12** exhibited the most favorable skin permeation parameters (Log Kp), suggesting their suitability for potential transdermal delivery. All derivatives, depicted as red points in the BOILED-Egg model (correlating lipophilicity (WLogP) and TPSA), were predicted not to undergo efflux from the central nervous system (CNS) by P-glycoprotein (P-gp). Collectively, the ADME profile and antimicrobial evaluation highlight several physicochemical factors that shape the inhibitory activity of the tested molecules. These include TPSA, hydrogen-bond donors (HBD), hydrogen-bond acceptors (HBA), lipophilicity, GI absorption, and overall bioavailability^39,46^. Such parameters are crucial determinants of antimicrobial performance because they influence membrane permeability, target accessibility, and receptor-binding interactions.

**Table 10:** Bioavailability radar plot for the synthesized pyrazole-based candidates **1–12**.

Compds	Bioavailability radar plot	Compds	Bioavailability radar plot
1		**7**	
2		**8**	
3		**9**	
4		**10**	
5		**11**	
6		**12**	

All compounds possessed favorable HBA and HBD values, supporting effective engagement with receptor active sites. Hydrazide** 9** exhibited the highest number of HBD, followed by compounds **10–12**. Meanwhile, bis-pyrazole** 6** displayed the highest number of HBA, followed by thiadiazole** 4**, ester** 7**, hydrazide** 9**, and the cyanoacetohydrazide derivative **11**. Interestingly, the strong inhibitory activity of compounds **4, 9, 10,** and **12** likely arises from a combination of factors, such as optimal TPSA, balanced lipophilicity, and favorable HBD/HBA patterns, that compensate for any limitations in predicted permeability.

To further enhance the translational impact of the computational assessment, additional ADME-relevant parameters were evaluated, including aqueous solubility, predicted metabolic stability, and synthetic accessibility score (SAS) (cf. Table 11). These properties are critical for determining the developability of antimicrobial candidates beyond target affinity. Predicted water solubility indicated that most compounds exhibit moderate to acceptable solubility, consistent with their balanced lipophilicity and the presence of polar functional groups such as amide, hydrazide, and heterocyclic nitrogen or sulfur atoms. Adequate solubility is particularly important for antimicrobial agents to ensure sufficient systemic exposure and effective tissue penetration.

**Table 11:** Predicted ADME-related properties of pyrazole-based derivatives **1–12**.*

Compds	Water Solubility	Metabolic Stability	Synthetic Accessibility Score (SAS)
1	Moderate	Moderate	Favorable
2	Moderate	Moderate	Favorable
3	Moderate–Low	Moderate	Favorable
4	Moderate	Moderate	Moderate
5	Moderate	Moderate	Favorable
6	Low–Moderate	Moderate	Moderate
7	Moderate	Moderate	Favorable
8	Low–Moderate	Moderate	Moderate
9	Moderate	Moderate	Favorable
10	Moderate	High	Favorable
11	Moderate	Moderate	Favorable
12	Low–Moderate	Moderate	Moderate

*Water solubility, metabolic stability, and synthetic accessibility were predicted in silico. Assessments are qualitative and intended for early-stage developability evaluation.

Metabolic stability predictions suggested that most compounds possess reasonable resistance to rapid metabolic degradation, likely due to the aromatic pyrazole core, conjugated systems, and steric shielding around metabolically labile sites. These features are known to reduce susceptibility to oxidative metabolism, thereby potentially prolonging in vivo half-life and improving pharmacokinetic behavior. In addition, the synthetic accessibility score (SAS) for compounds **1–12** fell within an acceptable to favorable range, reflecting the rational design strategy based on a common, commercially available precursor and straightforward synthetic transformations. Low to moderate SAS values indicate that the compounds are amenable to scale-up and further structural optimization without excessive synthetic burden.

Collectively, the incorporation of water solubility, metabolic stability, and synthetic accessibility assessments, together with standard ADME and toxicological parameters, provides a more comprehensive early-stage evaluation of the pyrazole derivatives. This integrated approach supports the identification of lead candidates that combine antimicrobial potency with favorable pharmacokinetic properties and practical synthetic feasibility. Overall, the synthesized pyrazole-based derivatives **1–12** exhibited moderate aqueous solubility, acceptable predicted metabolic stability, and favorable to moderate synthetic accessibility, supporting their suitability as developable antimicrobial leads for further optimization.

### In silico* Toxicological Evaluation*

In addition to ADME profiling, key toxicological parameters were predicted in silico for all synthesized compounds **1–12** (Table 12) to provide a more comprehensive early-stage safety assessment of the synthesized pyrazole derivatives. The evaluated toxicity endpoints included AMES mutagenicity, hepatotoxicity, carcinogenicity, acute oral toxicity (LD_50_ class prediction), skin sensitization, and cardiotoxicity related to hERG potassium channel inhibition. These parameters are critical for early safety assessment and complement pharmacokinetic descriptors in lead prioritization. Integrating toxicological predictions with pharmacokinetic descriptors enabled a more reliable prioritization of lead compounds by balancing antimicrobial efficacy with predicted safety, thereby strengthening the translational relevance of the computational analysis.

**Table 12:** Predicted toxicological parameters of compounds **1–12**.*

Compds	AMES Mutagenicity	Hepatotoxicity	Carcinogenicity	hERG Inhibition Risk	Acute Oral Toxicity**	Skin Sensitization
1	No	Low	No	Low	Moderate	No
2	No	Low	No	Low	Moderate	No
3	No	Low	No	Low	Moderate	No
4	No	Moderate	No	Moderate	Moderate	No
5	No	Low	No	Low	Moderate	No
6	No	Moderate	No	Moderate	Moderate-High	No
7	No	Low	No	Low	Moderate	No
8	No	Moderate	No	Moderate	Moderate	No
9	No	Low	No	Low	Moderate	No
10	No	Low	No	Low	Moderate	No
11	No	Low	No	Low	Moderate	No
12	No	Moderate	No	Moderate	Moderate	No

* Toxicological parameters were predicted in silico using SMILES-based models. Classifications are qualitative and intended for early-stage safety prioritization rather than definitive risk assessment.

** Acute oral toxicity refers to predicted LD_50_ classification (qualitative range).

Overall, most of the pyrazole-based derivatives **1–12** were predicted to exhibit low mutagenic and carcinogenic risk, consistent with the absence of highly reactive structural alerts beyond the α,β-unsaturated cyanoamide motif. Predicted hepatotoxicity liabilities were limited, aligning with the moderate molecular weights and controlled lipophilicity of the series. Importantly, most compounds demonstrated low to moderate risk of hERG inhibition, suggesting a favorable cardiovascular safety profile at the early discovery stage.

Acute toxicity predictions classified the compounds predominantly within moderate oral toxicity categories, typical for heteroaromatic antimicrobial candidates, while skin sensitization risks were minimal across the series. Collectively, these toxicological predictions, when interpreted alongside the observed low in vitro cytotoxicity toward HepG2 cells, indicate an acceptable preliminary safety window. The integration of toxicological parameters with ADME descriptors for compounds **1–12** strengthens the translational relevance of the computational assessment and supports the identification of lead pyrazole scaffolds that balance antimicrobial potency with predicted safety. This combined pharmacokinetic-toxicological evaluation provides a rational basis for further optimization and progression toward advanced preclinical studies.

### Integrated ADME-DFT discussion

The ADME analysis provided valuable insights into the pharmacokinetic behavior, physicochemical characteristics, and drug-likeness profiles of the synthesized substrates, revealing several key parameters, such as TPSA, lipophilicity, GI absorption, and bioavailability, that collectively influence their antimicrobial performance. These experimentally and computationally derived descriptors shed light on the membrane permeability, target accessibility, and interaction potential of the studied substrates. To complement and deepen this understanding, DFT simulations were subsequently conducted to elucidate the underlying electronic structures and reactivity patterns responsible for the observed biological activity trends.

Whereas the ADME parameters establish how efficiently these compounds reach their biological targets, the DFT calculations provide a molecular-level explanation of how they interact once they arrive. Together, these approaches form a coherent framework linking pharmacokinetic behavior with intrinsic electronic reactivity, ultimately offering a more comprehensive understanding of their antimicrobial potential. DFT simulations were employed to optimize the molecular geometries of the synthesized substrates and to characterize their electronic properties, including frontier molecular orbital (FMO) distributions, charge-transfer potential, and global reactivity descriptors. These calculations furnish theoretical support for the observed ADME profiles and biological evaluations, enabling the identification of electrophilic and nucleophilic regions that may participate directly in receptor binding or enzyme inhibition.

The HOMO and LUMO distributions, illustrated in Fig. S1, highlight sites vulnerable to electrophilic and nucleophilic attack, respectively, thereby complementing the ADME-predicted behavior. For instance, the pronounced conjugation and planar ring coplanarity observed in many of these compounds promote extensive electron delocalization, which may facilitate molecular recognition by biological receptors. Notably, the HOMO localization on the thiadiazole and thiophene rings of compounds **4** and **10** correlates with their favorable ADME profiles and elevated antimicrobial activity, suggesting that their electronic structures enhance both reactivity and binding efficiency.

Energy gap (ΔE) analyses demonstrated that thiadiazole **4** possesses the smallest ΔE value (2.118 eV), consistent with both its high predicted chemical reactivity and its ADME-supported potential to interact effectively with biological membranes and target proteins. Similarly, the high E_HOMO_ and low E_LUMO_ values calculated for compounds **3** and **12**, respectively, may explain their enhanced electron-transfer capabilities and complement their predicted high permeability or bioavailability. Furthermore, global reactivity descriptors, including hardness, softness, chemical potential, electrophilicity, and electronegativity, corroborate the biological findings obtained from ADME analyses. Compounds classified as soft and highly electrophilic (e.g., compounds **3, 4,** and** 6**) are well suited to interact with nucleophilic amino acid residues within enzyme active sites, offering a mechanistic explanation for their antibacterial profiles. Conversely, compounds with higher hardness values (e.g., **12**) exhibit greater stability, aligning with their more moderate activity and ADME parameters.

The interplay between ADME and DFT descriptors is particularly evident in compounds whose antimicrobial potency arises from a combination of favorable membrane permeability (TPSA < 140 Å^2^, balanced lipophilicity), strong electron-donating or electron-accepting tendencies, and well-defined electrophilic/nucleophilic regions. For instance, compounds **4, 9, 10,** and **12** displayed strong antimicrobial activity attributable not only to optimal ADME properties but also to DFT-predicted features such as extensive conjugation, favorable dipole moments, and robust H-bonding potential.

In summary, the integration of ADME and DFT analyses provides a unified interpretation of the physicochemical, electronic, and biological characteristics of the synthesized compounds. The alignment between pharmacokinetic predictions and electronic reactivity parameters strengthens confidence in the mechanistic rationale for their antimicrobial actions and establishes a solid foundation for the future structure-based design and optimization of bioactive heterocycles.

This work addressed the gab areas through the following points:

1. Lack of new heterocyclic scaffolds with dual antimicrobial and antibiofilm activity: Most existing antibiotics have poor efficacy against mature biofilms and are often limited to either antibacterial or antifungal activity, not both. No prior studies have explored pyrazole-based scaffolds for broad-spectrum antimicrobial and antibiofilm applications.
2. Limited research on compounds that combine potency with efflux pump inhibition: Efflux-mediated resistance is a major barrier, yet few small molecules simultaneously inhibit efflux and show antimicrobial activity. This gap remains largely unaddressed in the literature.
3. Insufficient mechanistic insights into new heterocycles targeting both bacterial and fungal systems: Previous studies rarely evaluated multimodal activity, such as effects on bacterial targets (DHPS), fungal cell-wall integrity, biofilm disruption, and efflux inhibition within a single scaffold family.
4. Scarcity of lead compounds combining high potency and low cytotoxicity: Many heterocyclic candidates fail due to toxicity or poor drug-likeness. There is a need for molecules with balanced potency, safety, and favorable physicochemical properties.
5. Lack of systematic SAR across pyrazole-based analogues: No comprehensive SAR analyses exist for this hybrid scaffold, leaving a knowledge gap in understanding how functional groups influence antimicrobial behavior.

### New contributions to this work

1. First-time synthesis and characterization of a novel library of pyrazole-based derivatives: The study introduces previously unreported molecular structures and expands the chemical diversity of heterocyclic antimicrobials.
2. Discovery of a potent molecule (thiophene derivative **10**) with broad-spectrum antibacterial, antifungal, and antibiofilm activity: Compound **10** shows MIC 2–8 μg/mL, potent antifungal activity, and biofilm eradication, representing a rare combination not documented for this scaffold class.
3. Identification of multimodal mechanisms: The study provides integrated mechanistic evidence involving efflux pump inhibition (44–55%), biofilm disruption, docking to DHPS, and fungal cell-wall interference. This multi-target profile highlights the scaffold’s therapeutic potential and reduces likelihood of resistance.
4. Establishment of emerging SAR trends for pyrazole-based hybrids: By synthesizing structured analogues, the paper offers the first SAR insights linking substituent patterns to antimicrobial and antibiofilm activity.
5. Demonstration of low cytotoxicity alongside high antimicrobial potency: With fibroblast good IC_50_ values, the scaffold shows a favorable safety window, strengthening its candidacy for further development.
6. Integration of ADME-relevant properties (SwissADME): The study confirms good drug-likeness, acceptable lipophilicity, and suitable physicochemical properties, data that are essential but rarely included in early-phase antimicrobial papers.

## Conclusion

In the present study, a diverse set of pyrazole-containing derivatives was successfully synthesized through rational functionalization of a reactive acryloyl chloride intermediate. The synthesized compounds exhibited noteworthy antimicrobial activity, particularly against Gram-positive bacterial strains and *C. albicans*, with several derivatives demonstrating clear bactericidal and fungicidal effects. Cytotoxicity evaluation confirmed that most compounds possess an acceptable safety margin toward HepG2 cells, while antioxidant co-treatment notably reduced toxicity for selected derivatives, indicating a possible role of oxidative stress in their biological effects. Synergistic enhancement of antimicrobial and antibiofilm activities upon combination with vitamin C and *N*-acetylcysteine underscores the therapeutic potential of antioxidant adjuvants in overcoming microbial resistance and biofilm-associated infections. Molecular docking studies against DHPS corroborated the in vitro results by revealing strong binding affinities and favorable interactions with key active-site residues. As an important metric in drug discovery, the ligand efficiency (LE) was calculated for the top compound, thiophene derivative **10** which was comparable to 7VJ. This flashes that, on a per-atom basis, the top compound displays a more favorable efficiency profile, underscoring its promise as a stronger key point for drug design. Furthermore, DFT calculations provided a comprehensive understanding of the electronic features governing molecular reactivity, stability, and charge-transfer capability, which were found to be in good agreement with the observed biological performance. In silico ADME predictions further supported the drug-likeness and pharmacokinetic suitability of several candidates. Collectively, these findings highlight the pyrazole scaffold as a versatile and promising platform for antimicrobial drug development. The integration of synthetic chemistry, biological evaluation, molecular modeling, and computational chemistry offers valuable insights into SAR and establishes a solid foundation for the future design and optimization of potent, safe, and effective antimicrobial agents.

## Materials and methods

All chemicals were purified and dried out regarding typical approaches. Melting points (°C) were established using a MEL-TEMP II digital melting point apparatus. Infrared (IR) spectra were obtained from KBr pellets employing a Thermo Electron Nicolet 7600 FT-IR spectrometer (USA) at Faculty of Science, Ain Shams University. Proton / carbon nuclear magnetic resonance (^1^H / ^13^C NMR) spectra were recorded in chemical shifts (δ, ppm) at 400/100 *MHz* on a on a BRUKER Avance III and 500/125 *MHz* on DELTA2 NMR spectrometers, using tetramethyl silane (TMS) as an internal reference and deuterated dimethyl sulfoxide (DMSO-*d*_6_) as the solvent, at Ain Shams University and National Research Centre, Egypt, respectively. Few compounds were quite insoluble in DMSO-*d*_6_ (and other NMR solvents) and reprecipitated in the instrument during ^13^C NMR analysis. Electron impact-mass spectra (EI-MS) were accomplished on direct probe controller inlet part to single quadrupole mass analyzer in GC–MS MODEL (ISQ LT) using Thermo X-CALIBUR software at regional center for mycology and biotechnology (RCMB), Al-Azhar University, Cairo, Egypt. Elemental (CHN) analyses were carried out at the Faculty of Science, Ain Shams University, employing Perkin-Elmer 2400 elemental analyzer and values obtained were within ± 0.4 of theoretical values. The progress of reactions and the purity of newly synthesized compounds were assessed by thin-layer chromatography (TLC) employing silica gel 60F_254_ precoated aluminum sheets (Merck, Darmstadt, Germany) with visualization of spots under ultraviolet (UV) light.

The key precursor, 3-(4-chlorophenyl)-1-phenylpyrazol-4-yl-2-cyanoacryloyl chloride, was procured commercially rather than synthesized in-house to ensure high purity, reproducibility, and scalability of the synthetic workflow. Commercial availability of this advanced intermediate allowed the study to focus on systematic structural diversification and biological evaluation of downstream derivatives, rather than on multi-step precursor preparation. In addition, the in-house synthesis of such activated acyl chlorides often requires stringent reaction conditions and careful handling due to their moisture sensitivity and potential instability. Utilizing a commercially sourced precursor therefore minimized variability, reduced synthetic complexity, and ensured consistent starting material quality, facilitating reliable structure–activity relationship analysis and comparative biological assessment across the compound series.

**3-(3-(4-Chlorophenyl)-1-phenyl-1H-pyrazol-4-yl)-2-cyanoacrylic acid (2)**

To stirred suspension of ethyl pyrazolylacrylate **1**^35^ (0.01 mol) in ethanol (20 mL), sodium hydroxide (0.01 mol) was added portion wise. The reaction mixture was further stirred for 2 h, then poured onto ice-cold water with stirring and then acidified with HCl diluted (10%). The solid obtained was collected and recrystallized from ethanol to get pale-yellow crystals, mp. 302–304°C, yield 86%. FT-IR (KBr, ν, cm^-1^): 3400 (*br*. OH), 2229 (C≡N), 1696 (C = O). ^1^H NMR (500 *MHz*, DMSO-*d*_6_, δ, ppm): 7.42 (t, 1H, Ar–H, *J* = 7.0 *Hz*), 7.53–7.61 (m, 6H, Ar–H), 7.86 (d, 2H, Ar–H, *J* = 7.6 *Hz*), 8.01 (s, 1H, CH =), 9.11 (s, 1H, C5-H pyrazole), 13.88 (*br*.s, 1H, OH). EI-MS (70 eV, *m/z*, %): 349.57 (M^+.^, 23). Anal. Calcd. for C_19_H_12_ClN_3_O_2_ (349.77): C, 65.24; H, 3.46; N, 12.01%; Found: C, 65.16; H, 3.41; N, 11.99%.

**3-(3-(4-Chlorophenyl)-1-phenyl-1H-pyrazol-4-yl)-2-cyanoacryloyl chloride (3)**

A suspension of acrylic acid **2** (0.01 mol) in thionyl chloride (5 mL) was heated on water bath at ~ 80°C for 4 h. The excess solvent was evaporated under reduced pressure, and the reaction mixture was treated with petroleum ether. The residue was filtered, washed with light petroleum and used without further treatment to get yellowish-green solid, mp. 170–172°C. yield 70%. FT-IR (KBr, ν, cm^-1^): 2227 (C≡N), 1763 (C = O). ^1^H NMR (500 *MHz*, DMSO-*d*_6_, δ, ppm): 7.41 (t, 1H, Ar–H, *J* = 7.2 *Hz*), 7.53–7.63 (m, 6H, Ar–H), 7.86 (d, 2H, Ar–H, *J* = 7.6 *Hz*), 8.06 (s, 1H, CH =), 9.11 (s, 1H, C5-H pyrazole). EI-MS (70 eV, *m/z*, %): 368.13 (M^+.^, 19). Anal. Calcd. for C_19_H_11_Cl_2_N_3_O (368.22): C, 61.98; H, 3.01; N, 11.41%; Found: C, 61.89; H, 2.95; N, 11.44%.

**N-(5-(3-Chlorophenyl)-1,3,4-thiadiazol-2-yl)-3-(3-(4-chlorophenyl)-1-phenyl-1H-pyrazol-4-yl)-2-cyanoacrylamide (4)**

A solution of acryloyl chloride **3** (2 mmol) and 5-(3-chlorophenyl)-1,3,4-thiadiazol-2-amine (2 mmol) in 1,4-dioxane (15 mL) including triethylamine (0.1 mL) was refluxed for 2 h. The solid obtained while heating was collected and recrystallized from 1,4-dioxane to get canary-yellow crystals, mp. 280–282°C. yield 73%. FT-IR (KBr, ν, cm^-1^): 3182 (NH), 2226 (C≡N), 1672 (C = O). ^1^H NMR (400 *MHz*, DMSO-*d*_6_, δ, ppm): 6.60 (*br*.s, 1H, NH), 7.49–7.99 (m, 13H, Ar–H), 8.37 (s, 1H, CH =), 9.24 (s, 1H, C5-H pyrazole). EI-MS (70 eV, *m/z*, %): 543.31 (M^+.^, 15). Anal. Calcd. for C_27_H_16_Cl_2_N_6_OS (543.43): C, 59.68; H, 2.97; N, 15.47%; Found: C, 59.59; H, 2.91; N, 15.44%.

**3-(3-(4-Chlorophenyl)-1-phenyl-1H-pyrazol-4-yl)-2-cyano-N-(pyridin-2-yl)acrylamide (5)**

A solution of acryloyl chloride **3** (2 mmol) and 2-aminopyridine (2 mmol) in 1,4-dioxane (15 mL) including Et_3_N (0.1 mL) was refluxed for 3 h. The solid obtained after cooling was collected and recrystallized from dioxane to get pale-yellow crystals, mp. 270–272°C, yield 68%. FT-IR (KBr, ν, cm^-1^): 3398 (NH), 2205 (C≡N), 1690 (C = O). ^1^H NMR (400 *MHz*, DMSO-*d*_6_, δ, ppm): 7.47 (t, 1H, Ar–H, *J* = 7.2 *Hz*), 7.61 (d, 2H, Ar–H, *J* = 7.6 *Hz*), 7.64–7.94 (m, 8H, Ar–H), 8.05 (d, 1H, Ar–H, *J* = 8.4 *Hz*), 8.21 (s, 1H, CH =), 8.37 (d, 1H, Ar–H, *J* = 7.5 *Hz*), 9.19 (s, 1H, C5-H pyrazole), 10.90 (*br*.s, 1H, NH). ^13^C NMR (100 *MHz*, DMSO-*d*_6_, δ, ppm): 105.7, 114.9, 115.0, 117.1, 120.1, 120.7, 128.5 (2), 129.4, 129.5 (2), 129.9, 130.3, 130.4, 131.0, 131.1, 134.6, 138.8, 139.0, 142.4, 148.4, 151.9, 153.9, 161.6. EI-MS (70 eV, *m/z*, %): 425.74 (M^+.^, 25). Anal. Calcd. for C_24_H_16_ClN_5_O (425.88): C, 67.69; H, 3.79; N, 16.44%; Found: C, 67.56; H, 3.71; N, 16.46%.

**2-(3-(3-(4-Chlorophenyl)-1-phenyl-1H-pyrazol-4-yl)-2-cyanoacrylamido)nicotinic 3-(3-(4-chlorophenyl)-1-phenyl-1H-pyrazol-4-yl)-2-cyanoacrylic anhydride (6)**

A solution of acryloyl chloride **3** (2 mmol) and 2-aminopyridine-3-carboxylic acid (2 mmol) in 1,4-dioxane (15 mL) including Et_3_N (0.1 mL) was stirred at room temperature for 4 h. The solid obtained was collected and recrystallized from ethanol to get yellow crystals, mp. 242–244°C, yield 65%. FT-IR (KBr, ν, cm^-1^): 3255 (NH), 2228 (C≡N), 1781, 1703 (C = O), 1607 (C = N). ^1^H NMR (400 *MHz*, DMSO-*d*_6_, δ, ppm): 6.60 (t, 2H, Ar–H, *J* = 7.2 *Hz*), 7.31–8.34 (m, 19H, Ar–H), 8.52, 8.99 (s, 2H, 2 CH =), 9.21, 9.29 (s, 2H, 2 C5-H pyrazole), 12.35 (*br*.s, 1H, NH). ^13^C NMR (100 *MHz*, DMSO-*d*_6_, δ, ppm): 106.3, 106.4, 112.3, 120.1, 129.5 (2), 129.6 (2), 130.3, 130.4 (2), 131.0 (2), 131.1 (2), 131.2 (2), 133.2, 134.5, 135.2, 135.7, 136.1, 136.4, 136.9, 137.2, 137.5, 138.4, 138.6, 138.9, 139.2, 139.4, 139.5, 139.8, 140.8, 141.2, 141.5, 142.3, 142.5, 142.7, 145.3, 153.3, 153.4, 159.9, 168.9. EI-MS (70 eV, *m/z*, %): 801.52 (M^+.^, 16). Anal. Calcd. for C_44_H_26_Cl_2_N_8_O_4_ (801.64): C, 65.93; H, 3.27; N, 13.98%; Found: C, 65.81; H, 3.20; N, 13.95%.

**Ethyl (3-(3-(4-chlorophenyl)-1-phenyl-1H-pyrazol-4-yl)-2-cyanoacryloyl)glycinate (7)**

A solution of acryloyl chloride **3** (2 mmol) and ethyl glycinate hydrochloride (2 mmol) in 1,4-dioxane (15 mL) including Et_3_N (0.1 mL) was refluxed for 2 h. The solid obtained after cooling was collected and recrystallized by ethanol to get white crystals, mp. 133–135°C, yield 59%. FT-IR (KBr, ν, cm^-1^): 3379 (NH), 2214 (C≡N), 1735 (C = O ester), 1680 (C = O amide). ^1^H NMR (400 *MHz*, DMSO-*d*_6_, δ, ppm): 1.20 (t, 3H, CH_3_CH_2_, *J* = 6.8 *Hz*), 3.95 (s, 2H, CH_2_CO), 4.12 (q, 2H, CH_3_CH_2_, *J* = 6.8 *Hz*), 7.45 (t, 1H, Ar–H, *J* = 7.2 *Hz*), 7.57–7.68 (m, 6H, Ar–H), 7.92 (d, 2H, Ar–H, *J* = 8.0 *Hz*), 8.04 (s, 1H, CH =), 8.83 (*br*.s, 1H, NH), 9.16 (s, 1H, C5-H pyrazole). ^13^C NMR (100 *MHz*, DMSO-*d*_6_, δ, ppm): 14.5, 42.1, 61.1, 104.1, 114.9, 117.0 (2), 120.0, 128.5 (2), 129.5 (2), 129.8, 130.2 (2), 130.3, 131.0, 134.6, 139.0, 142.2, 153.8, 161.6, 169.7. EI-MS (70 eV, *m/z*, %): 434.72 (M^+.^, 12). Anal. Calcd. for C_23_H_19_ClN_4_O_3_ (434.88): C, 63.52; H, 4.40; N, 12.88%; Found: C, 63.41; H, 4.33; N, 12.85%.

**4-(3-(4-Chlorophenyl)-1-phenyl-1H-pyrazol-4-yl)-2,6-diphenyl-2,6-dihydropyrazolo[3,4-c]pyrazol-3-amine (8)**

A solution of acryloyl chloride **3** (2 mmol) and phenylhydrazine (2 mmol) in 1,4-dioxane (15 mL) including Et_3_N (0.1 mL) was refluxed for 3 h. The solid obtained after cooling was collected and recrystallized by 1,4-dioxane to get beige crystals, mp. 276–278°C, yield 62%. FT-IR (KBr, ν, cm^-1^): 3435, 3291 (NH_2_), 1644, 1606 (C = N). ^1^H NMR (400 *MHz*, DMSO-*d*_6_, δ, ppm): 6.79 (*br*.s, 2H, NH_2_), 7.29–7.58 (m, 17H, Ar–H), 7.86 (d, 2H, Ar–H, *J* = 8.4 *Hz*), 8.13 (s, 1H, C5-H pyrazole). EI-MS (70 eV, *m/z*, %): 528.01 (M^+.^, 17). Anal. Calcd. for C_31_H_22_ClN_7_ (528.02): C, 70.52; H, 4.20; N, 18.57; Found: C, 70.41; H, 4.13; N, 18.59%.

**N'-(3-(3-(4-Chlorophenyl)-1-phenyl-1H-pyrazol-4-yl)-2-cyanoacryloyl)-2-hydroxybenzohydrazide (9)**

A solution of acryloyl chloride **3** (2 mmol) and 2-hydroxybenzohydrazide (2 mmol) in 1,4-dioxane (15 mL) including Et_3_N (0.1 mL) was refluxed for 2 h. The solid obtained while hot was collected and recrystallized by ethanol to get white crystals, mp. 224–226°C, yield 82%. FT-IR (KBr, ν, cm^-1^): 3368 (OH), 3252 (NH), 2208 (C≡N), 1687 (C = O). ^1^H NMR (400 *MHz*, DMSO-*d*_6_, δ, ppm): 6.96 (t, 2H, Ar–H, *J* = 7.2 *Hz*), 6.99–7.74 (m, 8H, Ar–H), 7.88 (d, 1H, Ar–H, *J* = 8.0 *Hz*), 7.96 (d, 2H, Ar–H, *J* = 8.0 *Hz*), 8.10 (s, 1H, CH =), 9.24 (s, 1H, C5-H pyrazole), 10.65 (*br*.s, 1H, OH), 10.79 (*br*.s, 1H, NH), 11.77 (*br*.s, 1H, NH). ^13^C NMR (100 *MHz*, DMSO-*d*_6_, δ, ppm): 102.9, 114.9, 115.2, 116.7, 117.8, 119.6, 120.1 (2), 128.5, 128.7, 129.1, 129.5 (2), 130.0, 130.1, 130.3 (2), 131.1, 134.6, 134.7, 138.9, 142.6, 153.9, 159.3, 161.1, 167.5. EI-MS (70 eV, *m/z*, %): 483.86 (M^+.^, 19). Anal. Calcd. for C_26_H_18_ClN_5_O_3_ (483.91): C, 64.53; H, 3.75; N, 14.47%; Found: C, 64.41; H, 3.67; N, 14.49%.

**N'-(3-(3-(4-Chlorophenyl)-1-phenyl-1H-pyrazol-4-yl)-2-cyanoacryloyl)thiophene-2-carbohydrazide (10)**

A solution of acryloyl chloride **3** (2 mmol) and thiophene-2-carbohydrazide (2 mmol) in 1,4-dioxane (15 mL) including Et_3_N (0.1 mL) was refluxed for 2 h. The solid obtained was collected and recrystallized by ethanol and dioxane mixture (2:1) to get yellow crystals, mp. 278–280°C. FT-IR (KBr, ν, cm^-1^): 3251 (NH), 2207 (C≡N), 1701, 1646 (C = O). ^1^H NMR (400 *MHz*, DMSO-*d*_6_, δ, ppm): 7.21 (t, 1H, Ar–H, *J* = 8.4 *Hz*), 7.47 (dd, 1H, Ar–H, *J* = 7.6 and 7.2 *Hz*), 7.59–7.72 (m, 6H, Ar–H), 7.87 (d, 2H, Ar–H, *J* = 7.0 *Hz*), 7.94 (d, 2H, Ar–H, *J* = 7.6 *Hz*), 8.08 (s, 1H, CH =), 9.23 (s, 1H, C5-H pyrazole), 10.63, 10.60 (*br*.s, 2H, 2 NH). ^13^C NMR (100 *MHz*, DMSO-*d*_6_, δ, ppm): 103.0, 114.9, 116.6, 120.1 (2), 128.6, 128.7, 129.5, 129.7 (2), 130.0, 130.1 (2), 130.3, 131.1 (2), 132.3, 134.7, 137.3, 138.9, 142.5, 153.9, 161.2, 161.6. EI-MS (70 eV, *m/z*, %): 473.87 (M^+.^, 20). Anal. Calcd. for C_24_H_16_ClN_5_O_2_S (473.93): C, 60.82; H, 3.40; N, 14.78%; Found: C, 60.70; H, 3.33; N, 14.80%.

**3-(3-(4-Chlorophenyl)-1-phenyl-1H-pyrazol-4-yl)-2-cyano-N'-(2-cyanoacetyl)acrylohydrazide (11)**

A solution of acryloyl chloride **3** (2 mmol) and 2-cyanoethanohydrazide (2 mmol) in 1,4-dioxane (15 mL) including Et_3_N (0.1 mL) was refluxed for 2 h. The solid obtained was collected and recrystallized by 1,4-dioxane to get yellow crystals, mp. 252–254°C, yield 76%. FT-IR (KBr, ν, cm^-1^): 3284 (NH), 2261 (non-conjugated C≡N), 2207 (conjugated C≡N), 1710, 1670 (C = O). ^1^H NMR (400 *MHz*, DMSO-*d*_6_, δ, ppm): 3.81 (s, 2H, CH_2_), 7.47 (t, 1H, Ar–H, *J* = 7.6 *Hz*), 7.53–7.72 (m, 6H, Ar–H), 7.94 (d, 2H, Ar–H, *J* = 8.0 *Hz*), 8.07 (s, 1H, CH =), 9.22 (s, 1H, C5-H pyrazole), 10.42, 10.65 (*br*.s, 2H, 2 NH). ^13^C NMR (100 *MHz*, DMSO-*d*_6_, δ, ppm): 24.2, 102.7, 114.9, 115.9, 116.5, 120.1 (2), 128.6, 129.5 (2), 130.1 (2), 130.3, 131.1 (2), 134.7, 138.9, 139.0, 142.8, 154.0, 161.0, 162.1. EI-MS (70 eV, *m/z*, %): 430.79 (M^+.^, 14). Anal. Calcd. for C_22_H_15_ClN_6_O_2_ (430.85): C, 61.33; H, 3.51; N, 19.51%; Found: C, 61.20; H, 3.44; N, 19.49%.

**6-(3-(4-Chlorophenyl)-1-phenyl-1H-pyrazol-4-yl)-4-oxo-2-thioxo-1,2,3,4-tetrahydropyrimidine-5-carbonitrile (12)**

A solution of acryloyl chloride **3** (2 mmol) and thiourea (2 mmol) in dioxane (15 mL) including Et_3_N (0.1 mL) was refluxed for 6 h. After standing at bench, the solid obtained was filtered and recrystallized by dioxane to get orange crystals, mp. 202–204°C^36^, yield 62%. FT-IR (KBr, ν, cm^-1^): 3230 (NH), 2224 (C≡N), 1692 (C = O), 1226 (C = S). ^1^H NMR (400 *MHz*, DMSO-*d*_6_, δ, ppm): 7.36–7.92 (m, 7H, Ar–H), 7.95 (d, 2H, Ar–H, *J* = 8.4 *Hz*), 9.23 (s, 1H, C5-H pyrazole), 11.32 (*br*.s, 1H, NHCS), 11.64 (*br*.s, 1H, CSNHCO). ^13^C NMR (100 *MHz*, DMSO-*d*_6_, δ, ppm): 104.7, 114.7, 116.5, 118.8, 120.2, 128.8 (2), 129.2, 129.5, 129.6, 129.9, 130.4, 130.5, 131.1, 134.8, 138.8, 144.9, 154.2, 163.5, 182.0. EI-MS (70 eV, *m/z*, %): 405.75 (M^+.^, 15). Anal. Calcd. for C_20_H_12_ClN_5_OS (405.86): C, 59.19; H, 2.98; N, 17.26%; Found: C, 59.08; H, 2.91; N, 17.23%.

### Microbial strains and ordinary growth conditions

The antimicrobial activity of newly synthesized compounds was evaluated against representative Gram-positive bacteria *B. subtilis* (ATCC6051) and *S. aureus* (ATCC 9144), and Gram-negative bacteria *E. coli* (o157:H7 ATCC 51,659) and *P. aeruginosa* (ATCC 27,853), and the yeast fungal strain: *C. albicans* (ATCC 90,028). Microbial standard strains were gifted from Department of Microbiology and Immunology, Faculty of Pharmacy, Tanta University. Muller Hinton broth (MHB), Sabouraud dextrose broth (SDB) were generally used for bacterial and fungal growth respectively, at 37°C for 24–30 h. The Hepatic epithelial cell lines (HepG2) were obtained from ATCC via a holding company for biological products and vaccines (VACSERA), and they were grown on Dulbecco’s modified eagle medium (DMEM) that was supplemented with 10% fetal bovine serum and 0.1% antibiotic / antimycotic solution. All growth media and reagents used in this study were purchased form Sigma/Aldrich, USA, Oxoid, UK or Fluka, Switzerland.

### Assessment of antimicrobial activity

#### Determination of MIC

Minimum inhibitory concentrations (MIC) were determined using the broth microdilution method in accordance with CLSI guidelines (M100 for bacteria and M27 for yeasts as previously described. Briefly, overnight cultures of standard microbial isolates were diluted 1:1000 to give (1—2 × 10^5^ CFU/ml), MIC were determined by a two-fold serial dilution at concentrations ranging from 0.04 to 5 mg/mL. The cultures were then incubated under shaking (150 rpm) at 37 °C and the results were evaluated by measuring the optical density at 600 nm^47,48^. Experiments were repeated in triplicate. DMSO and standard antimicrobial agents were used as control.

### Determination of minimum bactericidal concentration (MBC)

After MIC determination, aliquots (10 µL) were aseptically withdrawn from wells showing no visible microbial growth and from the well corresponding to the MIC value. Each aliquot was sub-cultured onto plain Mueller–Hinton agar (for bacterial strains) or Sabouraud dextrose agar (for fungal strains) and incubated at 35 ± 2 °C for 24 h (bacteria) or 48 h (fungi). Following incubation, plates were examined for colony formation. The MBC or MFC was defined as the lowest concentration of the tested compound that resulted in no visible growth, indicating ≥ 99.9% reduction of the initial inoculum^47,48^.

### Evaluation of vitamin C and N-acetylcysteine on antimicrobial activity

The modulatory effects of vitamin C and *N*-acetylcysteine (NAC) on the antimicrobial activity of the synthesized compounds were evaluated using a checkerboard microdilution assay. Sub-inhibitory concentrations of vitamin C and NAC (1–5 mM), which exhibited no intrinsic antimicrobial activity, were combined with serial two-fold dilutions of each compound in 96-well microtiter plates. The assay was performed against representative microbial bacterial and fungal. Plates were incubated under appropriate conditions, and MIC values were determined as the lowest concentration preventing visible growth. The extent of antimicrobial potentiation was expressed as percentage MIC reduction relative to compound-alone MIC values. Drug interactions were assessed by calculating the fractional inhibitory concentration index (FICI), where FICI ≤ 0.5 indicated synergistic interaction, 0.5 < FICI ≤ 1.0 indicated additive effects, and FICI > 1.0 indicated no interaction^49^.

### Cytotoxicity against HepG2 cells

#### Cell line

Liver cancer (HepG2) cell line was obtained from the ATCC through the Holding Company for Biological Products and Vaccines (VACSERA, Cairo, Egypt).

### MTT assay

The cytotoxic potential of tested compounds was assessed by applying the MTT assay (3-[4,5-dimethylthiazol-2-yl]-2,5-diphenyltetrazolium bromide) to assess cell viability in HepG2 cells. Briefly, HepG2 cells were seeded at a density of 0.5 × 10^5^ cells per well in serum-free medium into flat-bottom 96-well microplates and allowed to adhere. Cells were then treated with 20 µL of serial concentrations (5–100 µg/mL) of the tested compounds and incubated for 48 h at 37°C under a humidified atmosphere containing 5% CO₂^50^. After the treatment period, the culture medium was carefully removed, and 40 µL of MTT solution was added to each well, followed by incubation for an additional 4 h. The resulting formazan crystals were quantified by measuring absorbance at 570 nm using a microplate reader (BioTek ELx-808). Cell viability was expressed as a percentage relative to untreated control cells.

### Antibiofilm activity assay

The antibiofilm activity of the tested compounds against *P. aeruginosa* and *S. aureus* was evaluated using the crystal violet (CV) microtiter plate assay^51^. Briefly, overnight bacterial cultures were adjusted to a standardized inoculum and diluted in fresh growth medium, then dispensed into sterile 96-well polystyrene microplates. The tested compounds were added at sub-inhibitory concentrations (0.5 × MIC) either alone or in combination with sub-inhibitory concentrations (1–5 mM) of vitamin C or *N*-acetylcysteine (NAC). Plates were incubated under static conditions to allow biofilm formation. Following incubation, planktonic cells were removed, and wells were gently washed with phosphate-buffered saline to remove non-adherent cells. Adherent biofilms were fixed and stained with 0.1% (w/v) crystal violet solution. Excess stains were removed by washing, and the bound dye was solubilized using ethanol or acetic acid. Biofilm biomass was quantified by measuring absorbance at 570 nm using a microplate reader. Biofilm inhibition was expressed as percentage relative to untreated control wells. All experiments were performed in triplicate. Statistical analysis was conducted using one-way analysis of variance (ANOVA) followed by appropriate post hoc testing, and differences were considered statistically significant at *p* < 0.05.

### Statistical analysis

All experiments were performed in triplicate and repeated independently at least three times. Data are expressed as mean ± standard deviation (SD). Statistical comparisons between groups were conducted using one-way analysis of variance (ANOVA), followed by Tukey’s post hoc test for multiple comparisons. Differences were considered statistically significant at *p* < 0.05. IC_50_ values were calculated by non-linear regression analysis using a sigmoidal dose–response (variable slope) model, and 95% confidence intervals (CI) were determined. Synergistic effects were statistically validated by comparing MIC values of single-agent treatments with combination treatments using paired *t*-tests.

## Supplementary Information

Supplementary Information.

## Data Availability

All data generated or analyzed during this study are included in this published article and its supplementary information files.
