# Supplementary material for: Design, synthesis, biological assessment, and integrated computational analysis of new pyrazole-based antimicrobial candidates
Source: Sci Rep. 2026 Jul 10;16:21618. doi: 10.1038/s41598-026-60094-9 (PMC13354578; doi:10.1038/s41598-026-60094-9)
Supplement: Supplementary file 1 — Supplementary Information. [file 41598_2026_60094_MOESM1_ESM.docx]

**Design, Synthesis, Biological Assessment, and Integrated Computational Analysis of New Pyrazole-Based Antimicrobial Candidates**

**Supporting information**

**Table S1**. Energy level distribution of frontier orbitals and global reactivity indices of compounds **3-12**.

| **Compds.** | ****E*** | ***E*_HOMO_**  ***(eV)*** | ***E*_LUMO_**  ***(eV)*** | **Δ*E***  ***(eV)*** | ***µ***  ***(Debye)*** | ***η***  ***(eV)*** | ***ς***  ***(eV^-1^)*** | ***μ_o_***  ***(eV)*** | ***∆N_max_*** | ***ω***  ***(eV)*** | ***n***  ***(eV^-1^)*** | ***Ip***  ***(eV)*** | ***EA***  ***(eV)*** | ***x***  ***(eV)*** |
| --- | --- | --- | --- | --- | --- | --- | --- | --- | --- | --- | --- | --- | --- | --- |
| **3** | 45.916 | -7.627 | -4.907 | 2.720 | 10.793 | 1.360 | 0.735 | -6.267 | 4.608 | 14.44 | 0.069 | 7.627 | 4.907 | 6.267 |
| **4** | 49.967 | -5.978 | -3.860 | 2.118 | -2.976 | 1.059 | 0.944 | -4.919 | 4..645 | 11.42 | 0.087 | 5.978 | 3.860 | 4.919 |
| **5** | 36.407 | -7.683 | -4.483 | 3.200 | -3.307 | 1.600 | 0.625 | -6.083 | 3.802 | 11.56 | 0.086 | 7.683 | 4.483 | 6.083 |
| **6** | 107.284 | -7.795 | -4.652 | 3.143 | 20.084 | 1.571 | 0.636 | -6.223 | 3.961 | 12.32 | 0.081 | 7.795 | 4.652 | 6.223 |
| **7** | 35.470 | -7.568 | -4.348 | 3.220 | -0.200 | 1.610 | 0.621 | -5.958 | 3.700 | 11.02 | 0.091 | 7.568 | 4.348 | 5.958 |
| **9** | 26.957 | -7.675 | -4.138 | 3.537 | -6.459 | 1.768 | 0.565 | -5.906 | 3.340 | 9.86 | 0.101 | 7.675 | 4.138 | 5.906 |
| **10** | 40.374 | -7.351 | -3.990 | 3.361 | -4.623 | 1.680 | 0.595 | -5.670 | 3.375 | 9.57 | 0.104 | 7.351 | 3.990 | 5.670 |
| **11** | 26.949 | -7.674 | -4.124 | 3.550 | -2.445 | 1.775 | 0.563 | -5.899 | 3.323 | 9.80 | 0.102 | 7.674 | 4.124 | 5.899 |
| **12** | 64.392 | -7.749 | -1.487 | 6.262 | 3.575 | 3.131 | 0.319 | -4.618 | 1.475 | 3.40 | 0.294 | 7.749 | 1.487 | 4.618 |

^*^*E*: Minimized Energy (kcal/mol) *µ*: Dipole/dipole *η:* Global Hardness

*ς:* Global Softness *μ_o:_* Chemical Potential *∆N_max_*: Additional electronic charge *ω:* Global Electrophilicity Index

*n*: Nucleophilicity Index *Ip*: Ionization Potential *EA*: Electron Affinity *x*: Electronegativity

**Compound 3**

**Compound 4**

**Compound 5**

**Compound 6**

**Compound 7**

**Compound 9**

**Compound 10**

**Compound 11**

**Compound 12**

**Fig. S1.** Optimized structures (left), HOMO (middle), and LUMO (right) for the synthesized compounds. Atom color index: Grey C, White H, Blue N, Red O, Yellow S, and Green Cl.


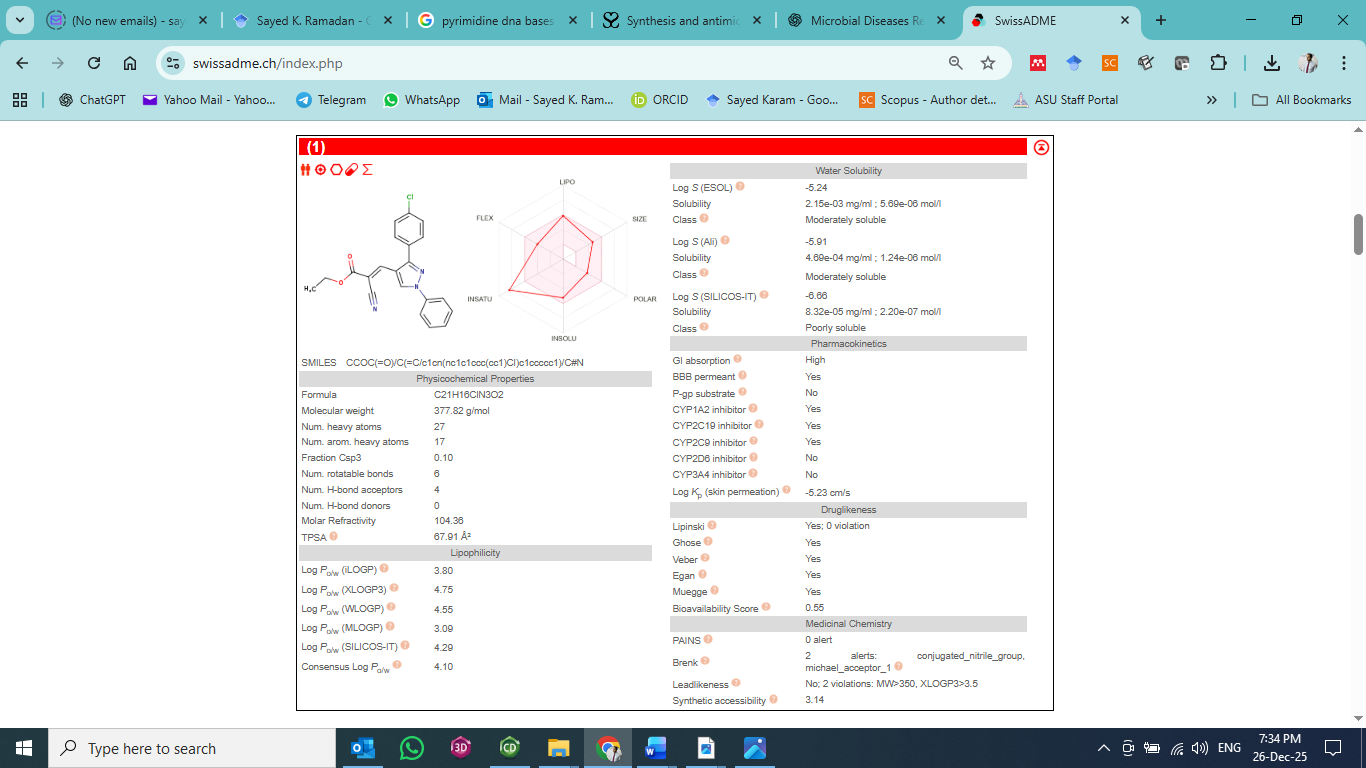


**Fig. S2.** ADME prediction of compound **1**.


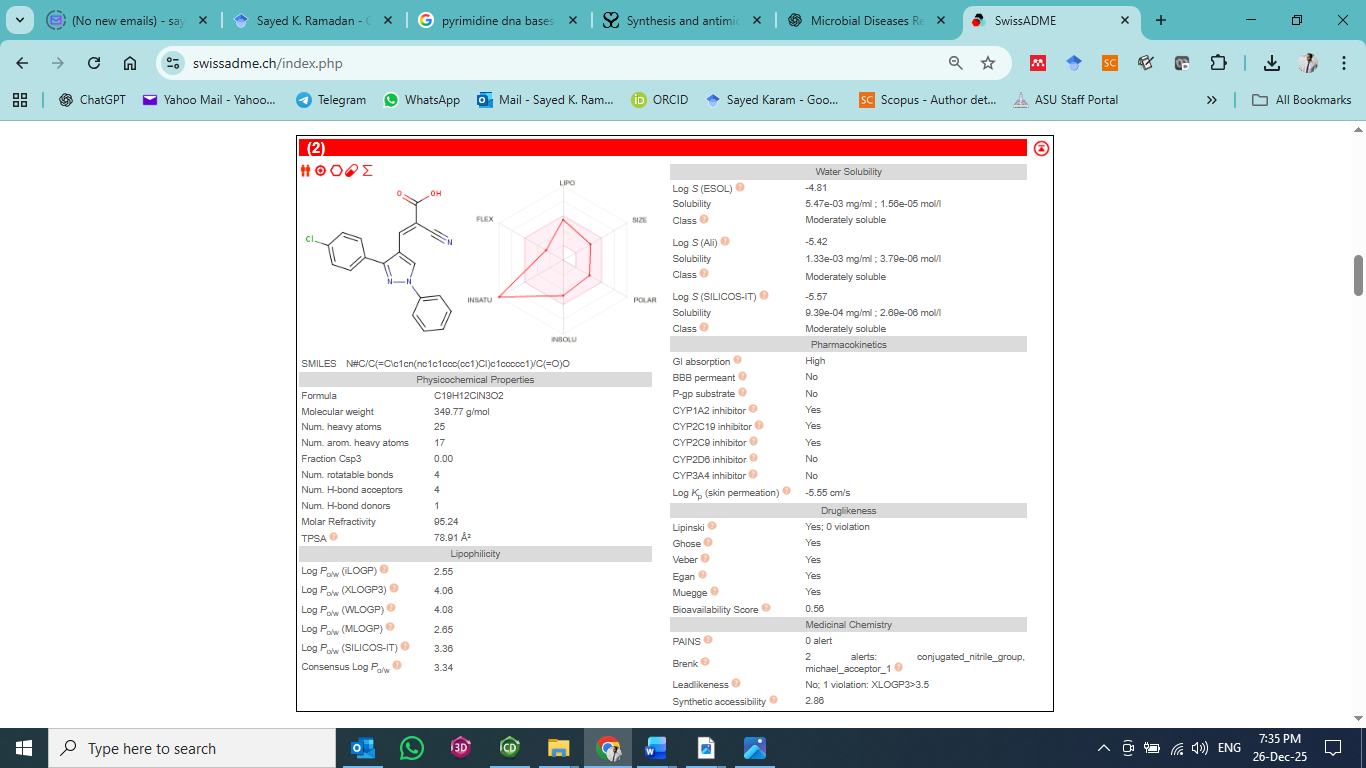


**Fig. S3.** ADME prediction of compound **2**.


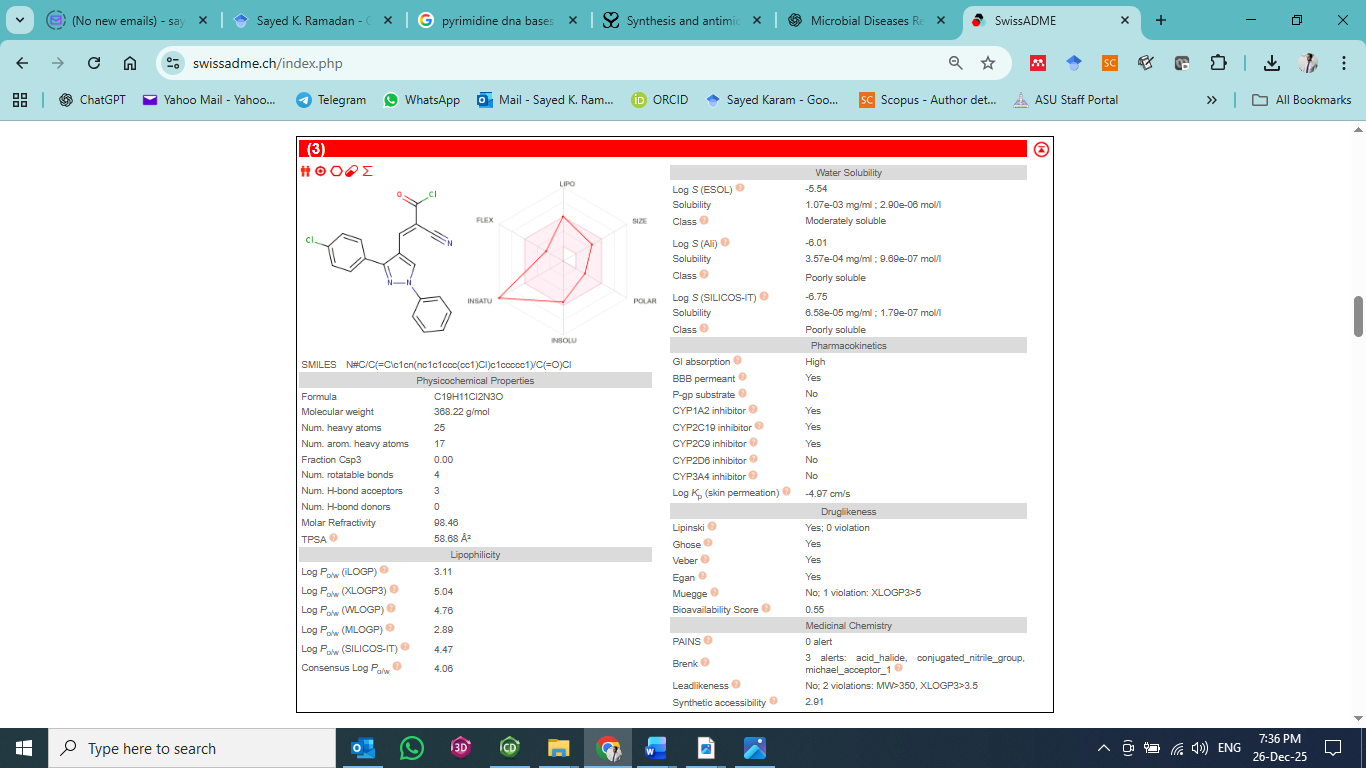


**Fig. S4.** ADME prediction of compound **3**.


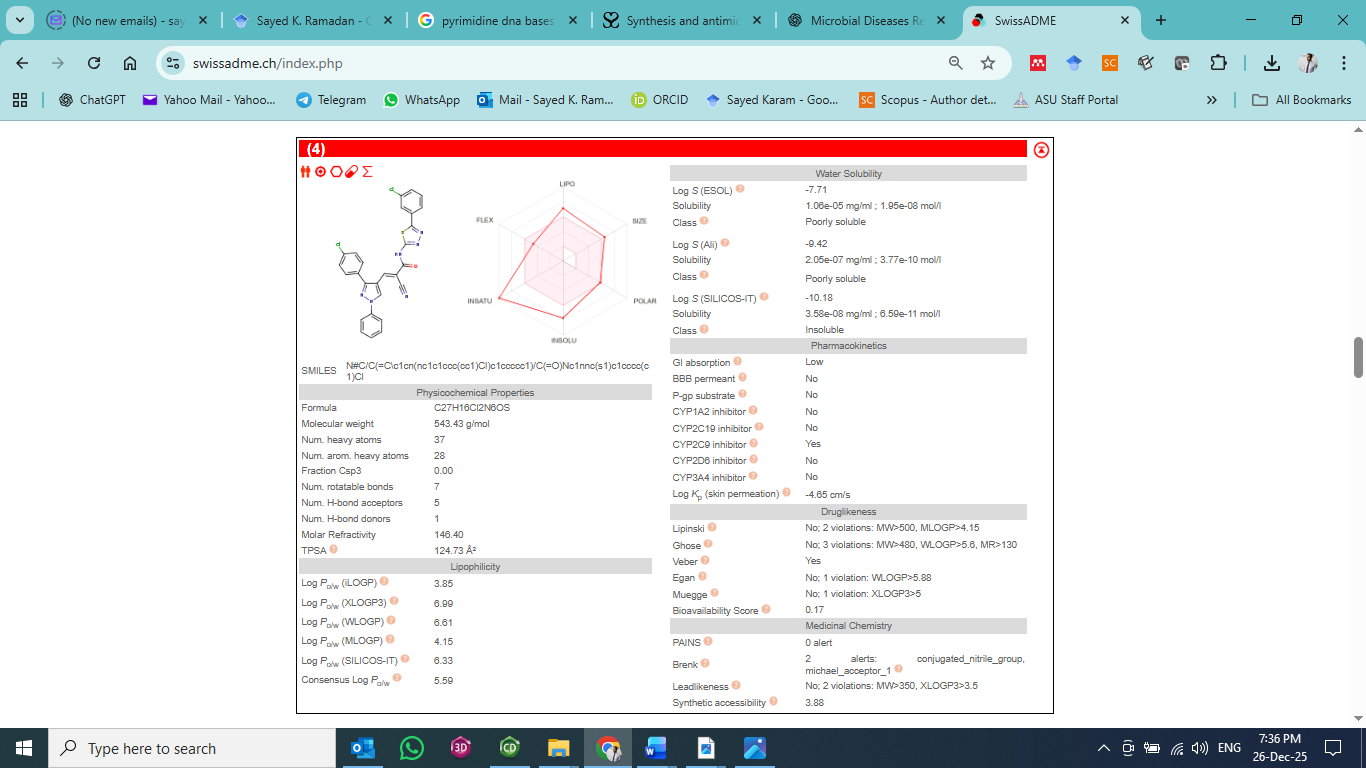


**Fig. S5.** ADME prediction of compound **4**.


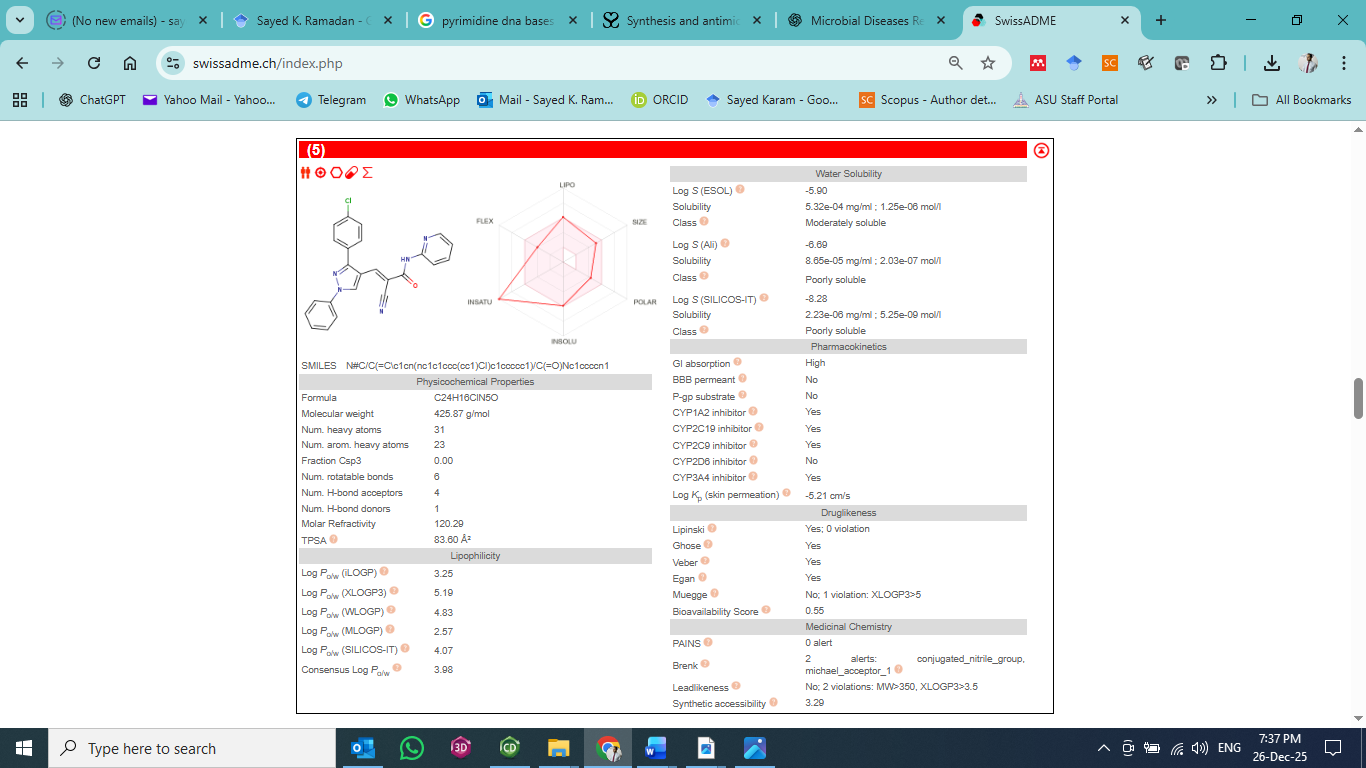


**Fig. S6.** ADME prediction of compound **5**.


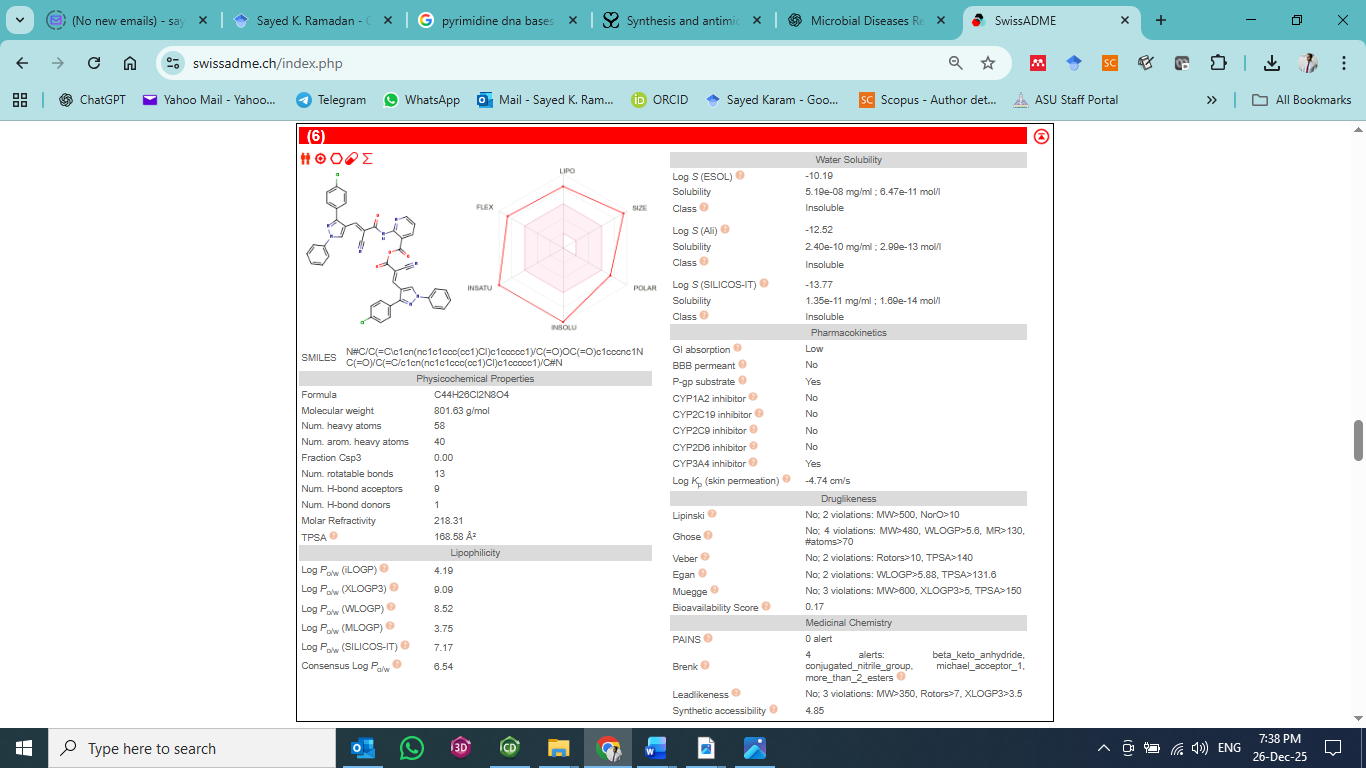


**Fig. S7.** ADME prediction of compound **6**.


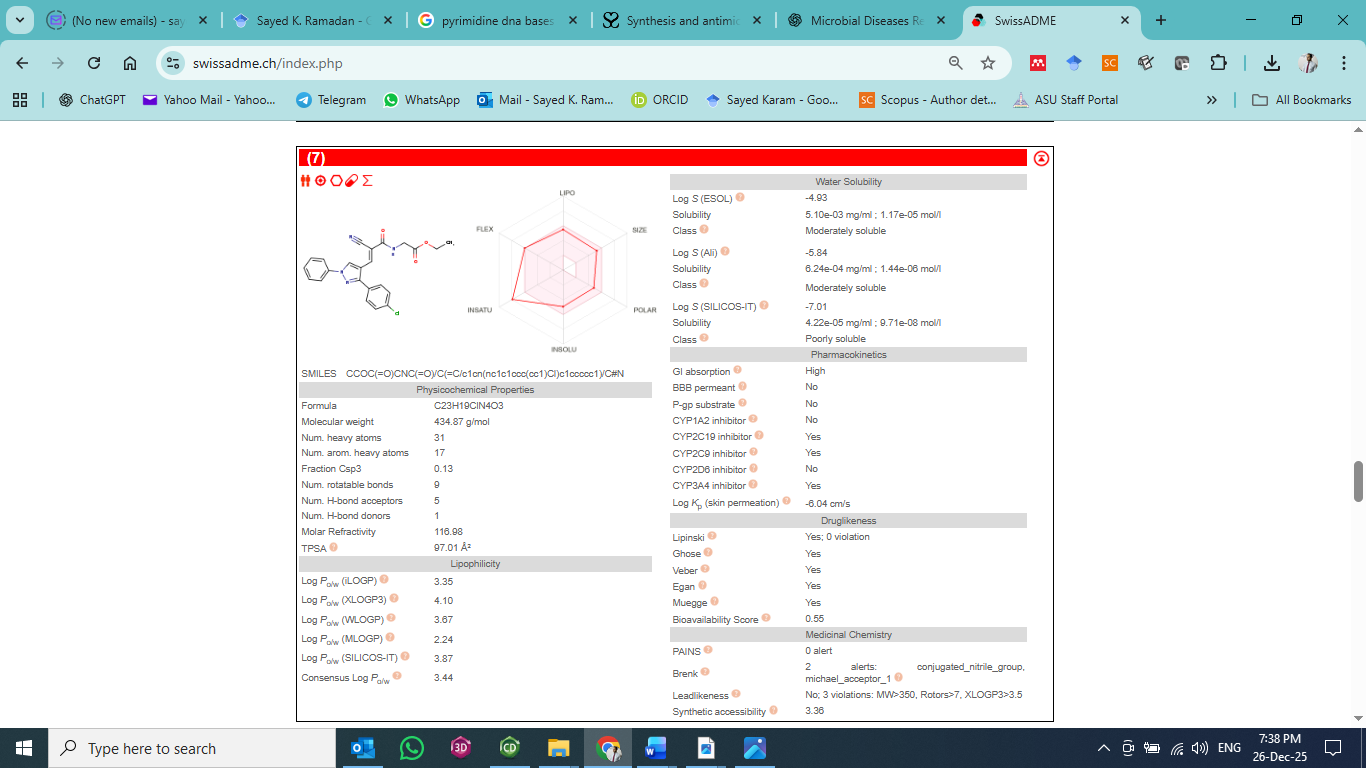


**Fig. S8.** ADME prediction of compound **7**.


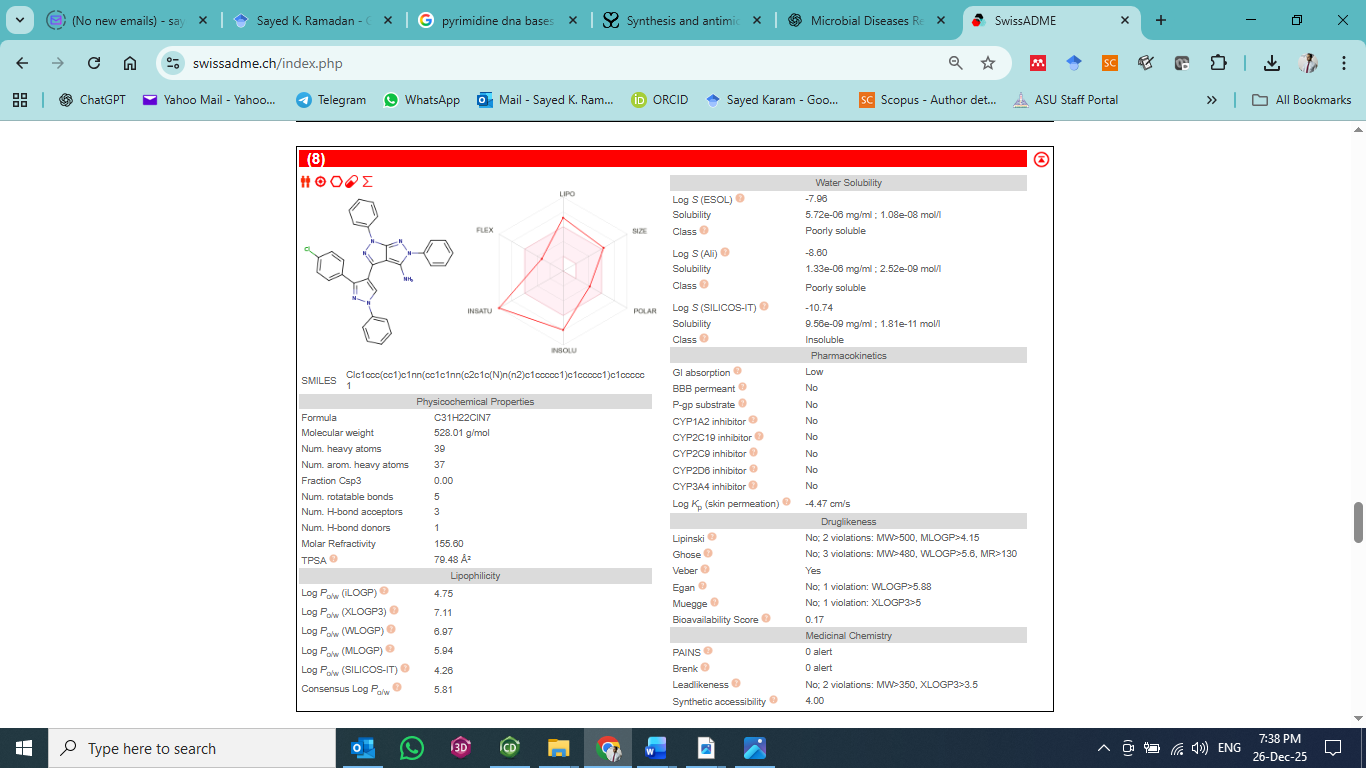


**Fig. S9.** ADME prediction of compound **8**.


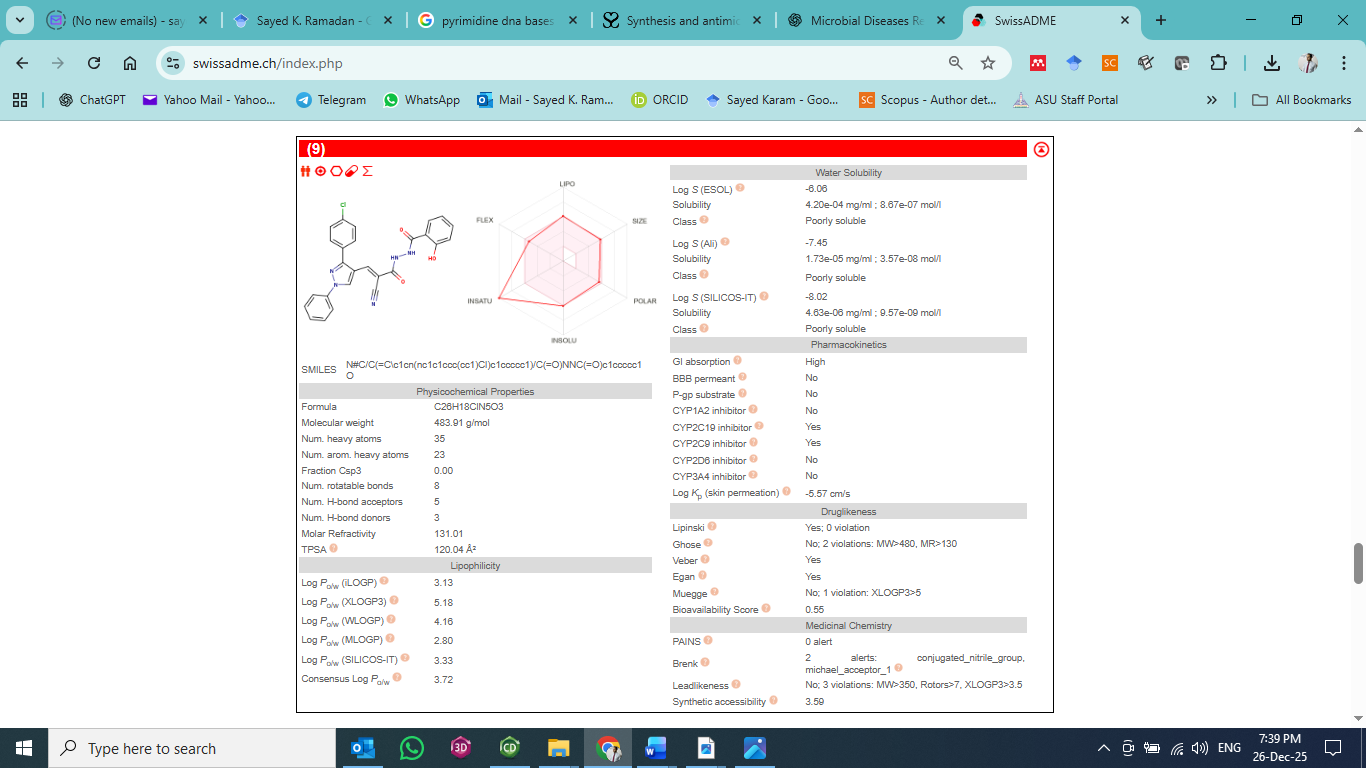


**Fig. S10.** ADME prediction of compound **9**.


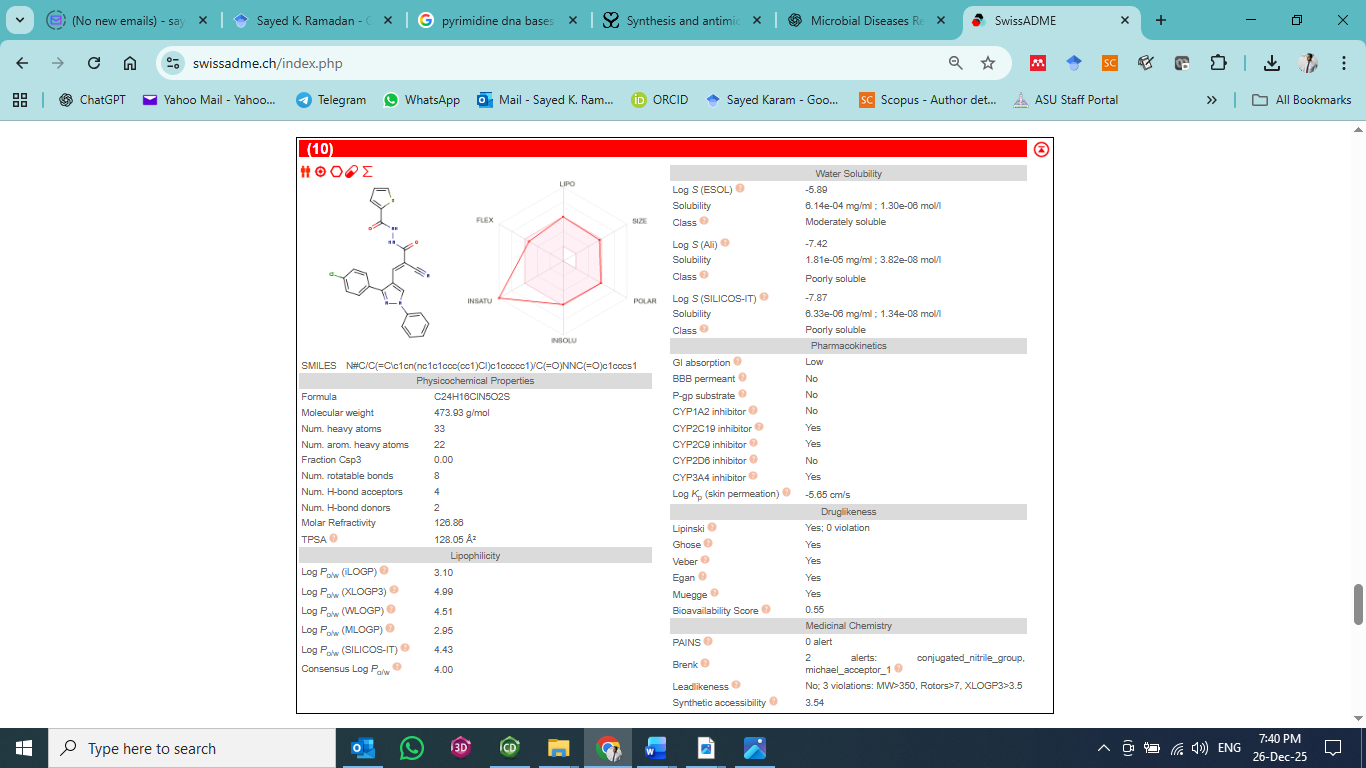


**Fig. S11.** ADME prediction of compound **10**.


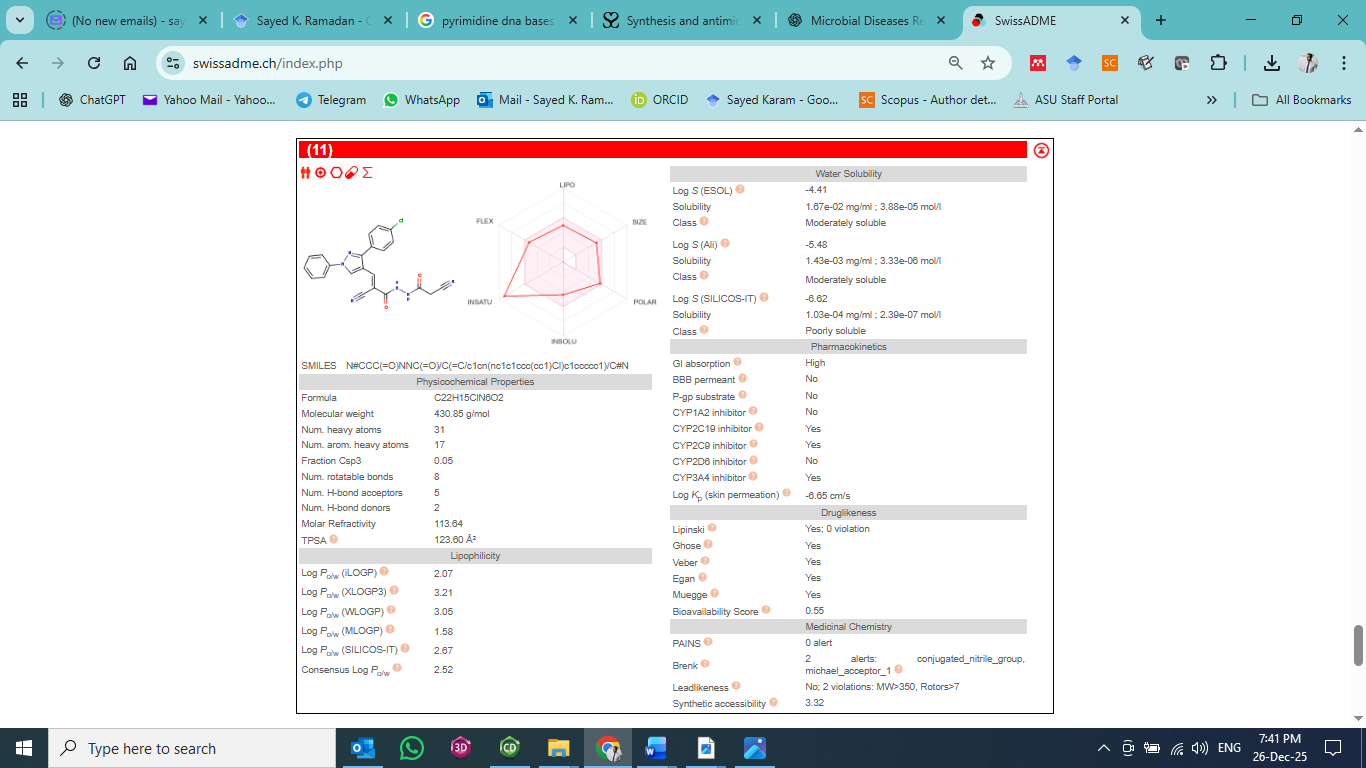


**Fig. S12.** ADME prediction of compound **11**.


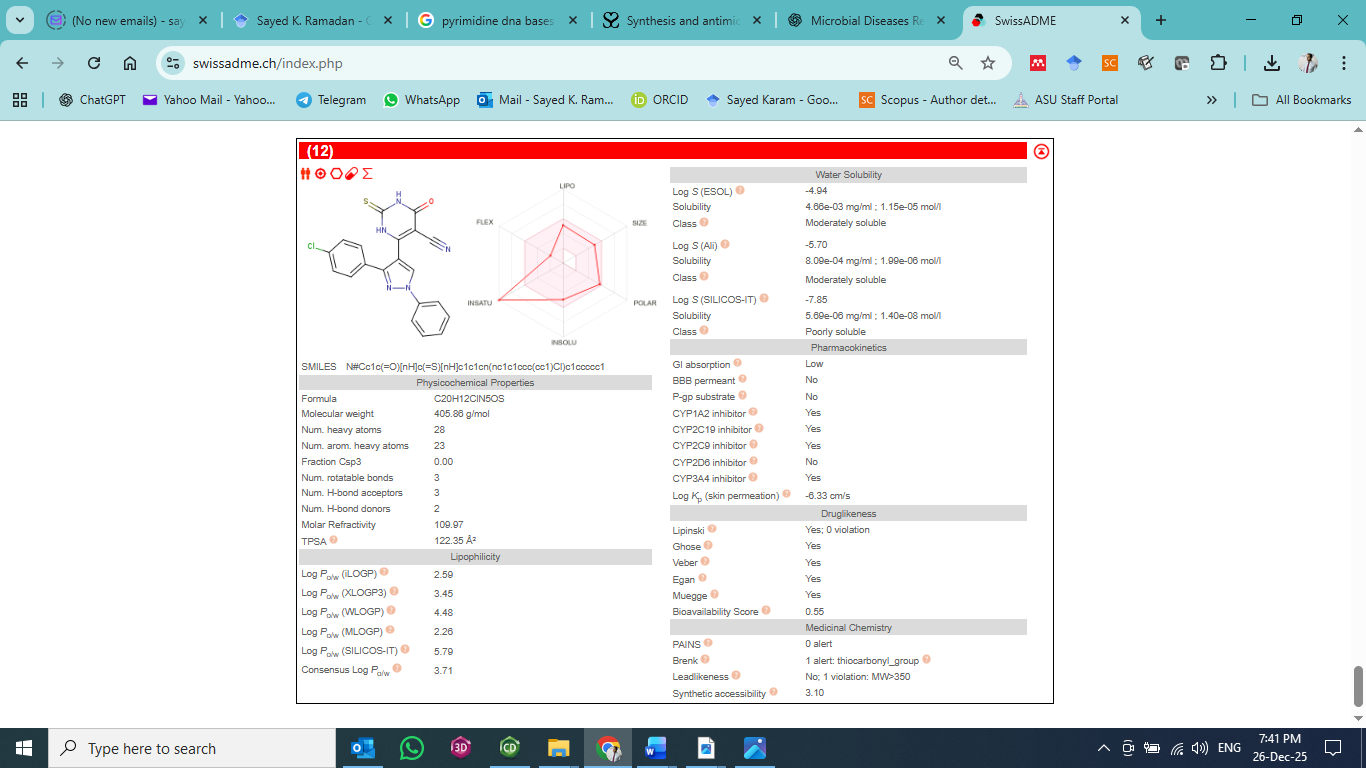


**Fig. S13.** ADME prediction of compound **12**.

**Table** **S2**. The violations and alerts of the prepared compounds **1-12** to pharmacokinetics rules.

| **Compds**. | Lipinski #violations | Ghose #violations | Veber #violations | Egan #violations | Muegge #violations | PAINS #alerts | Brenk #alerts | Leadlikeness #violations |
| --- | --- | --- | --- | --- | --- | --- | --- | --- |
| **1** | 0 | 0 | 0 | 0 | 0 | 0 | 2 | 2 |
| **2** | 0 | 0 | 0 | 0 | 0 | 0 | 2 | 1 |
| **3** | 0 | 0 | 0 | 0 | 1 | 0 | 3 | 2 |
| **4** | 2 | 3 | 0 | 1 | 1 | 0 | 2 | 2 |
| **5** | 0 | 0 | 0 | 0 | 1 | 0 | 2 | 2 |
| **6** | 2 | 4 | 2 | 2 | 3 | 0 | 4 | 3 |
| **7** | 0 | 0 | 0 | 0 | 0 | 0 | 2 | 3 |
| **8** | 2 | 3 | 0 | 1 | 1 | 0 | 0 | 2 |
| **9** | 0 | 2 | 0 | 0 | 1 | 0 | 2 | 3 |
| **10** | 0 | 0 | 0 | 0 | 0 | 0 | 2 | 3 |
| **11** | 0 | 0 | 0 | 0 | 0 | 0 | 2 | 2 |
| **12** | 0 | 0 | 0 | 0 | 0 | 0 | 1 | 1 |

**Materials and methods**

***DFT calculations***

Quantum chemical calculations based on DFT were achieved for the synthesized substrates using the B3LYP exchange-correlation functional in conjunction with the 6-311G(d) basis set, as implemented in the GAUSSIAN 09W program. This computational level is well recognized for presenting reliable insights into molecular stability and electronic reactivity. Geometry optimizations were achieved without applying any symmetry restrictions. The optimized molecular geometries and electronic structures were visualized using GaussView version 5.0.

***Molecular docking***

A molecular docking simulation was achieved using AutoDock tools (ADT, Version 1.5.7, http://vina.scripps.edu/), provided by the Scripps Research Institute (http://autodock.scripps.edu/resources/adt), to show the binding affinities of prepared ligands with amino acid residues at target protein based on their docking scores. The most active candidates were modeled using ChemBio3D Ultra 14.0, with partial charges assigned and energy minimized in line with previously recognized protocols. The target DHPS protein was gained from PDB database (https://www.rcsb.org, PDB ID: 5U0V), processed by correcting the structure, adding 3D hydrogen atoms, and operating energy minimization. Docking simulations were then conducted to estimate the ligands’ binding affinities within DHPS receptor pockets. The co-crystallized ligand (7VJ) was comprised as a reference standard, and docking parameters were constituted as described in prior studies. The resulting docking interactions were envisioned and analyzed operating Biovia Discovery Studio Visualizer (https://biovia-discovery-studio-64-bit-client.software.informer.com/4.5/). The docking protocol was validated using the co-crystallized ligand (7VJ) bound to its corresponding protein target by means of AutoDock Vina (<http://vina.scripps.edu/>).

***ADME Profiling***

The ADME properties of all substates were studied by the SwissADME free web tool (<http://www.swissadme.ch/index.php>). This platform facilitates the calculation of key physicochemical descriptors and offers *in silico* predictions related to pharmacokinetic behavior, drug-likeness, and medicinal chemistry relevance. Two-dimensional representations of the synthesized compounds were translated into SMILES (Simplified Molecular Input Line Entry System) notation and uploaded to the server for computational evaluation.

**Spectral Data**:


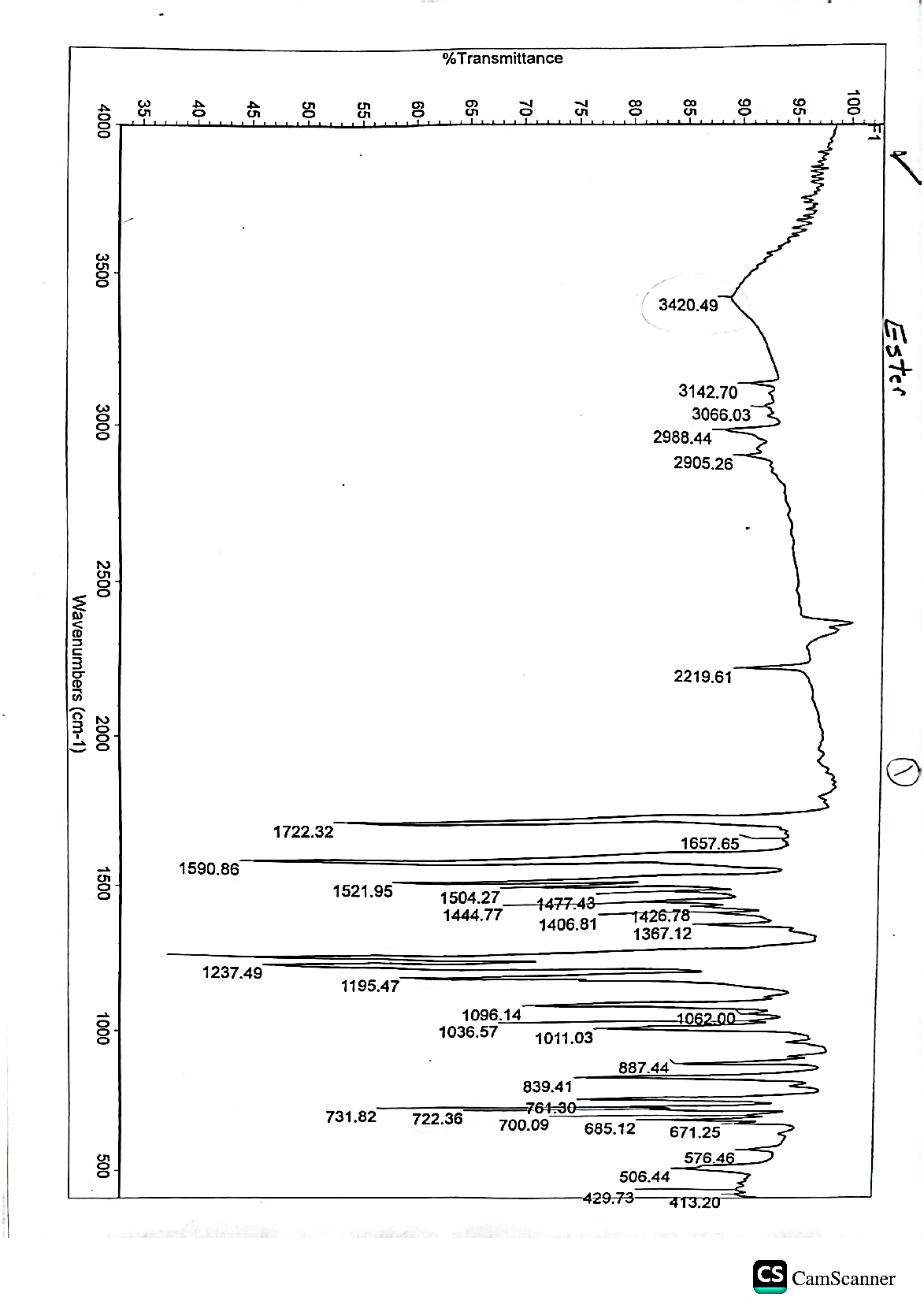


IR spectrum of compound 1


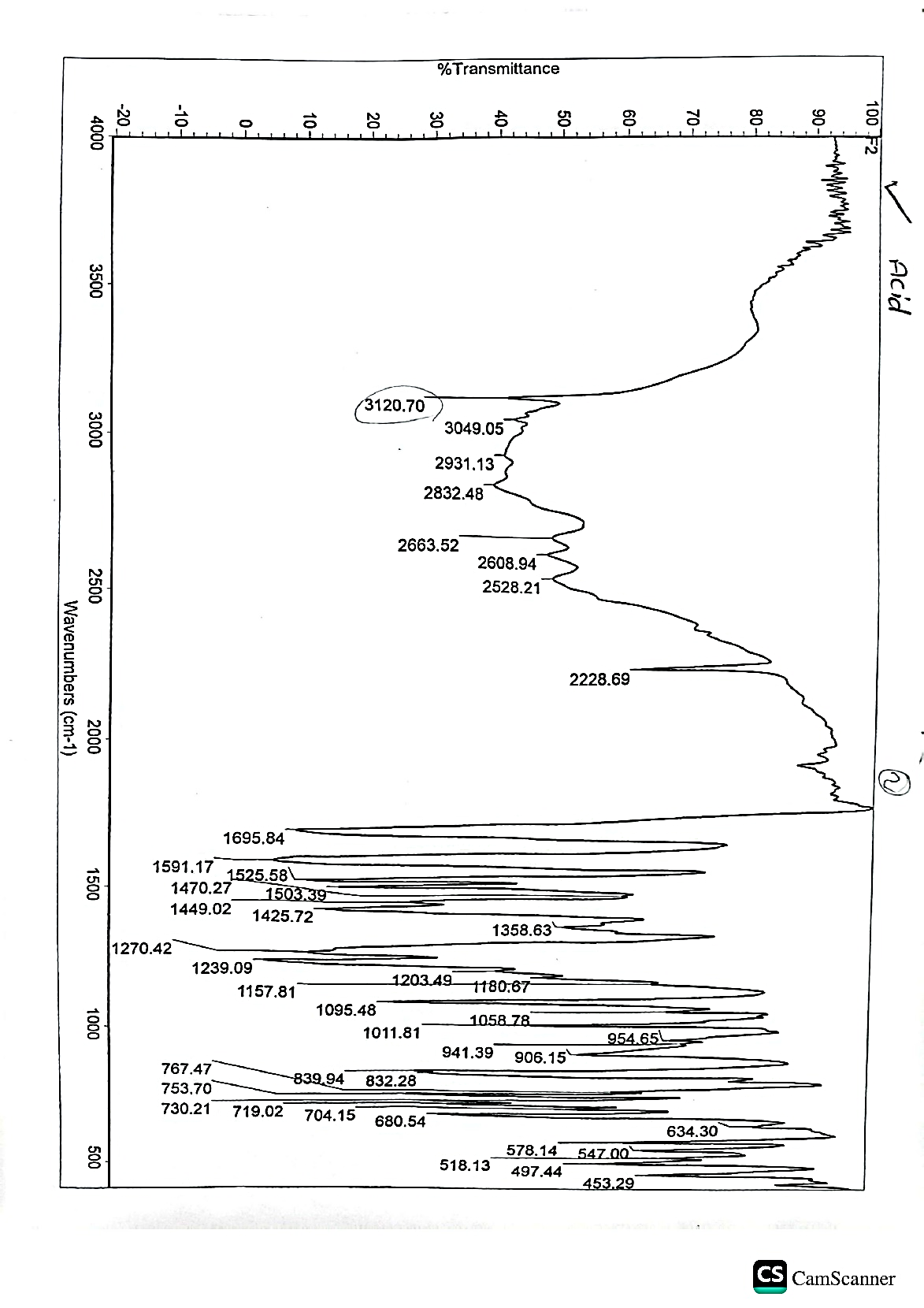


IR spectrum of compound 2


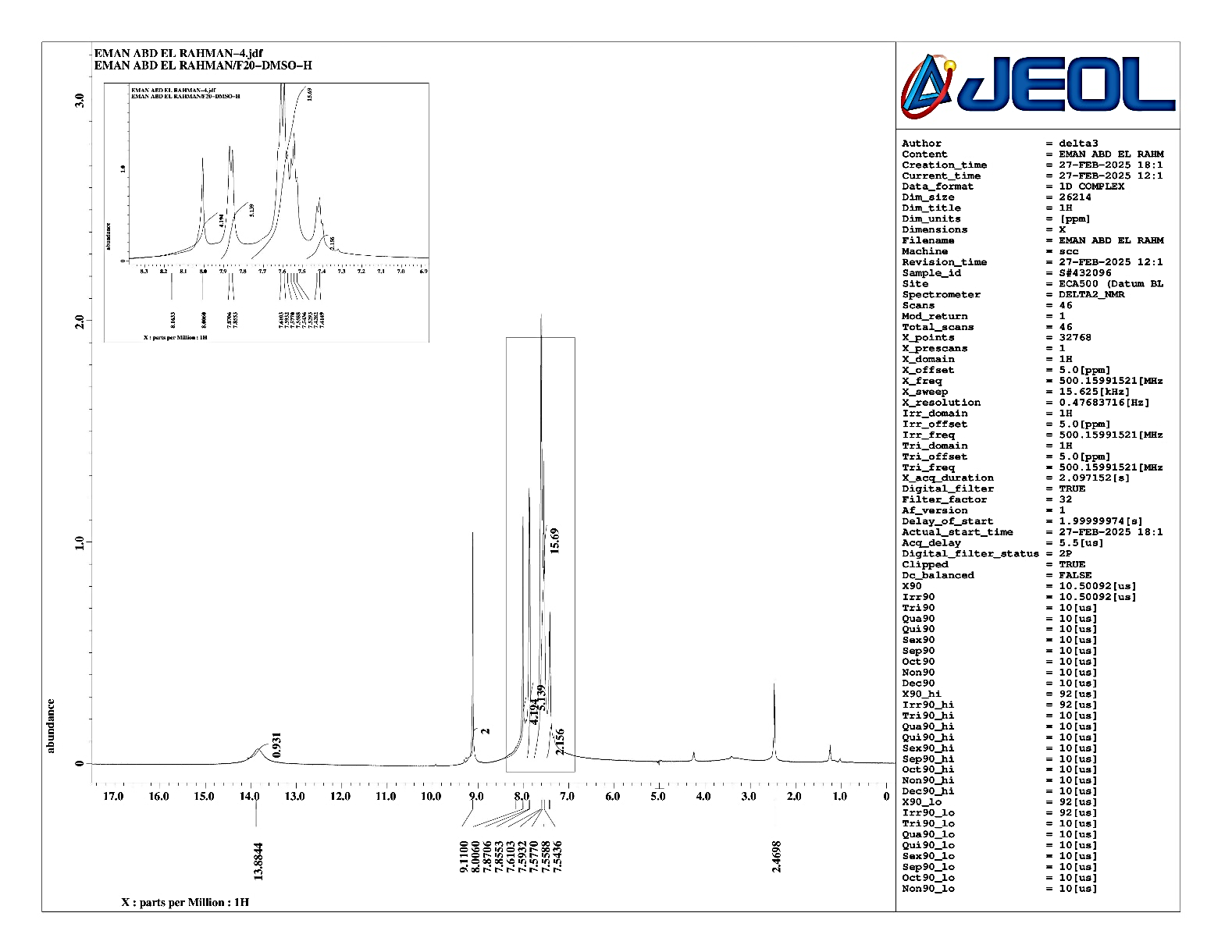


^1^H NMR spectrum (DMSO-*d*_6_) of compound 2


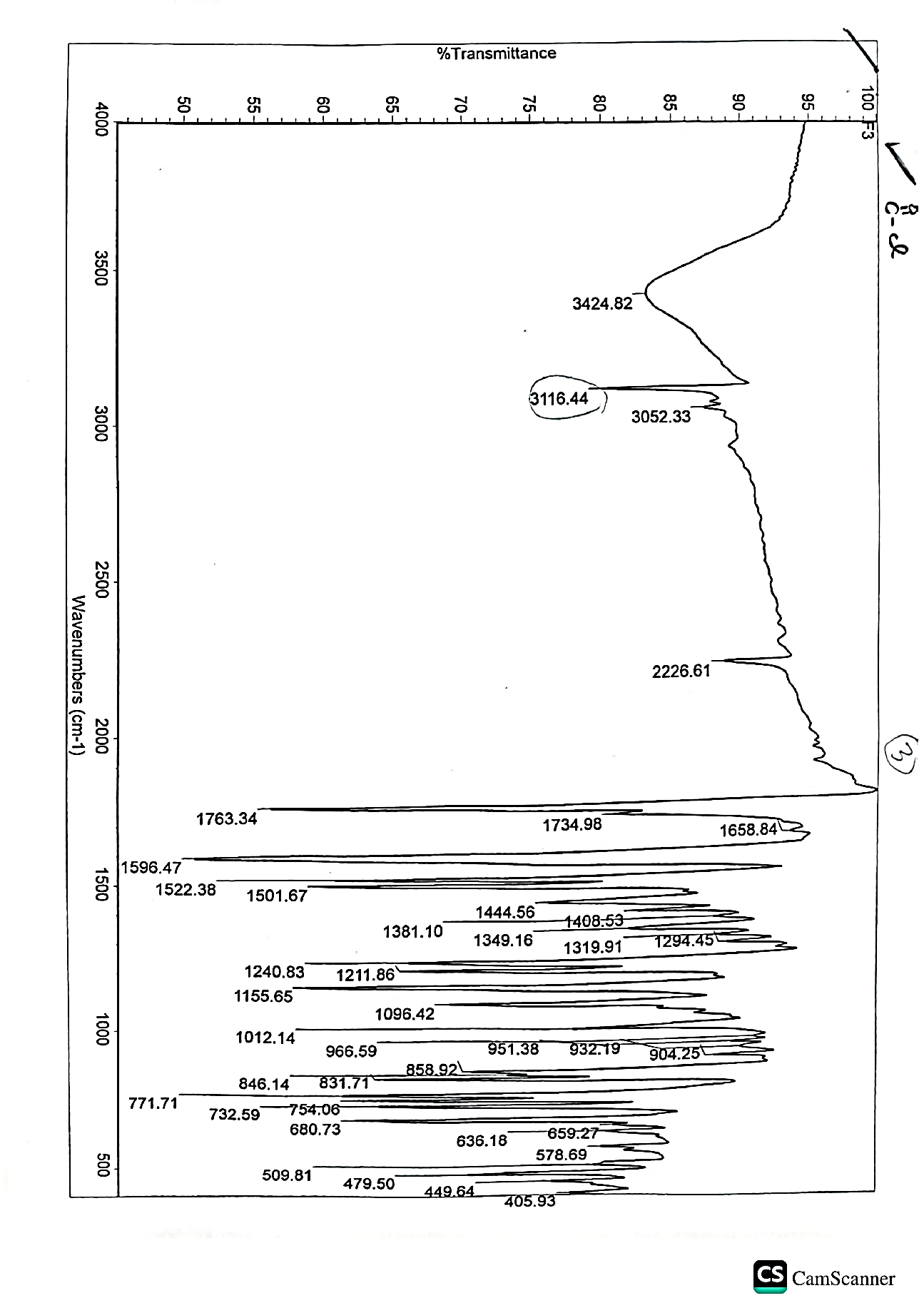


IR spectrum of compound 3


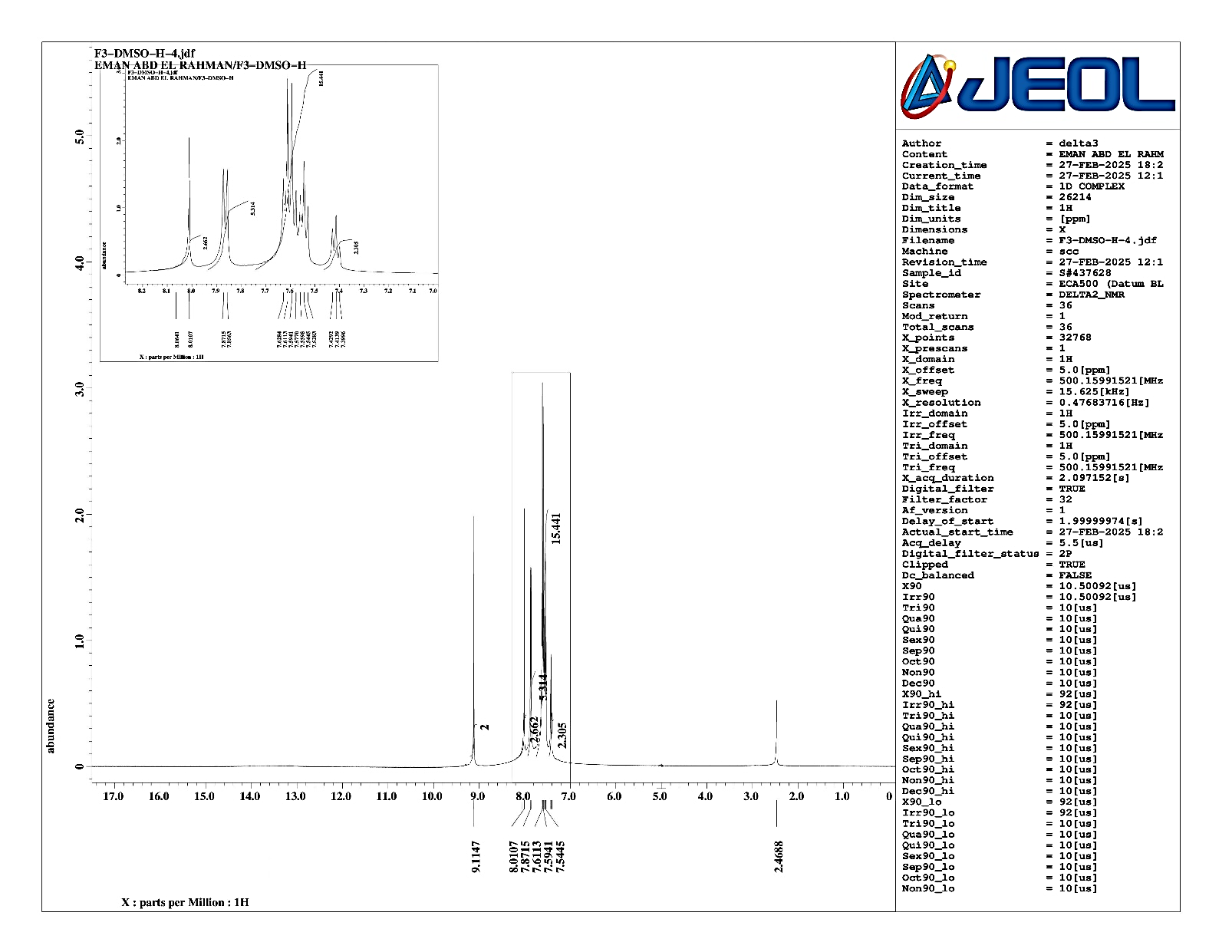


^1^H NMR spectrum (DMSO-*d*_6_) of compound 3


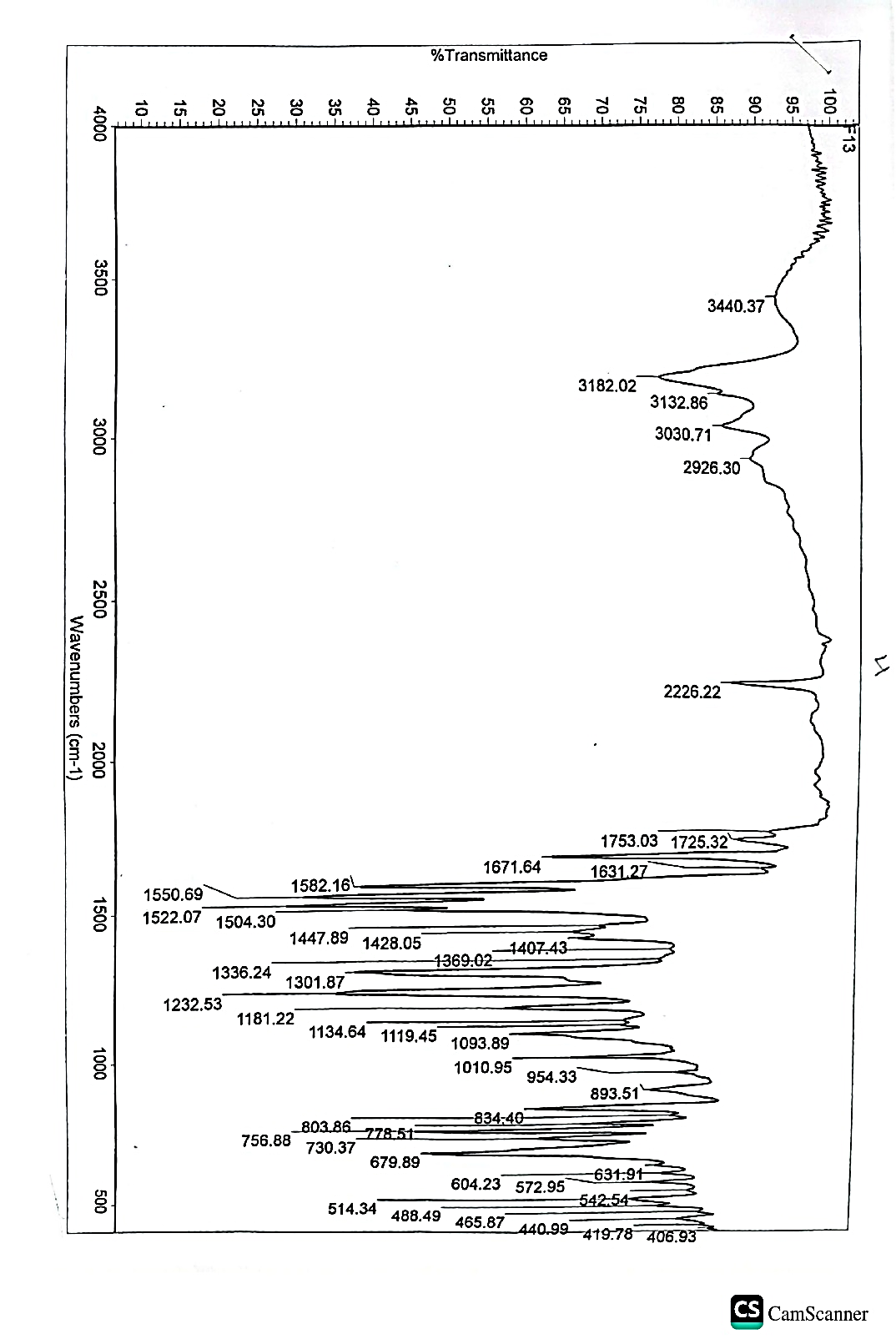


IR spectrum of compound 4


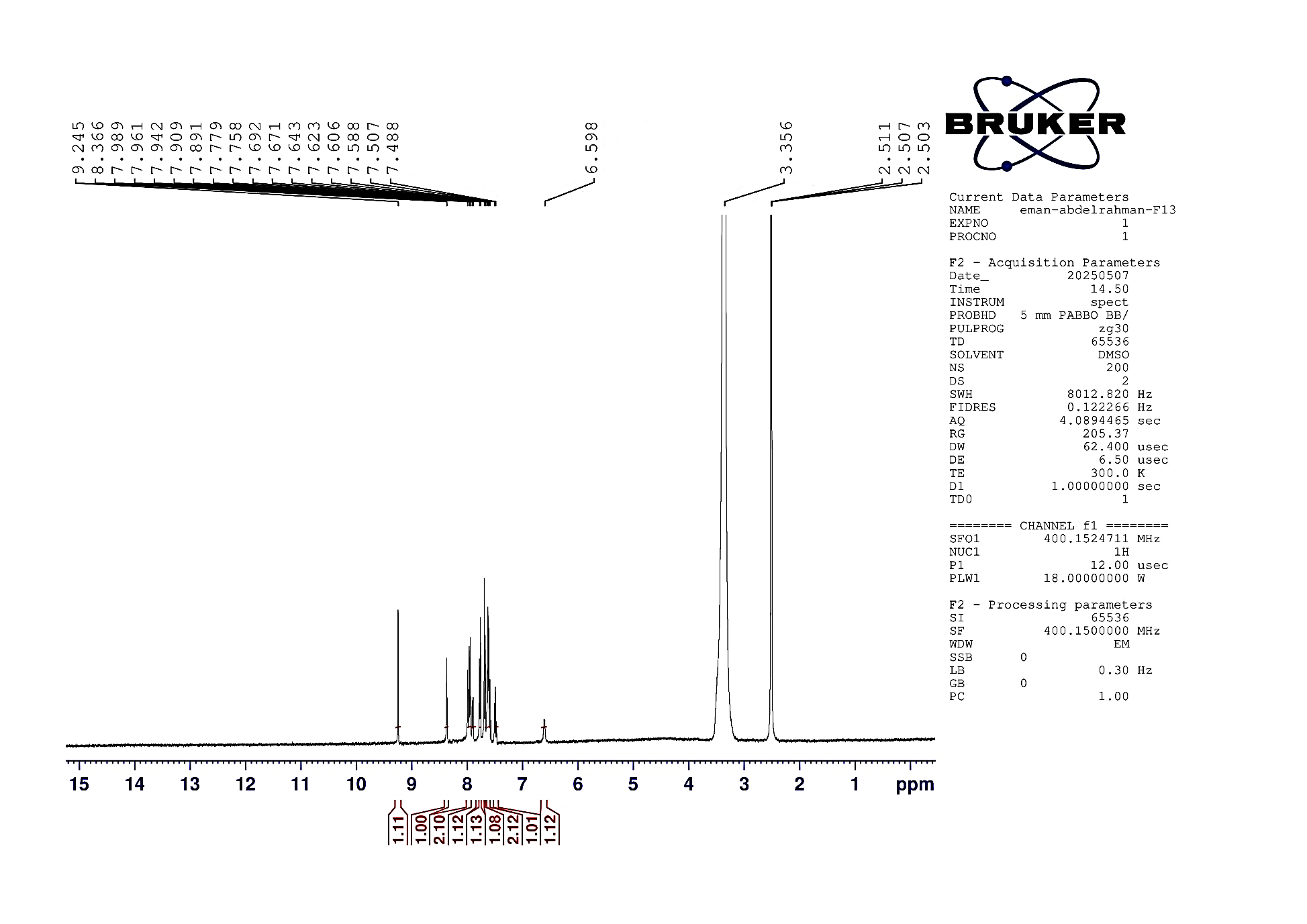


^1^H NMR spectrum (DMSO-*d*_6_) of compound 4


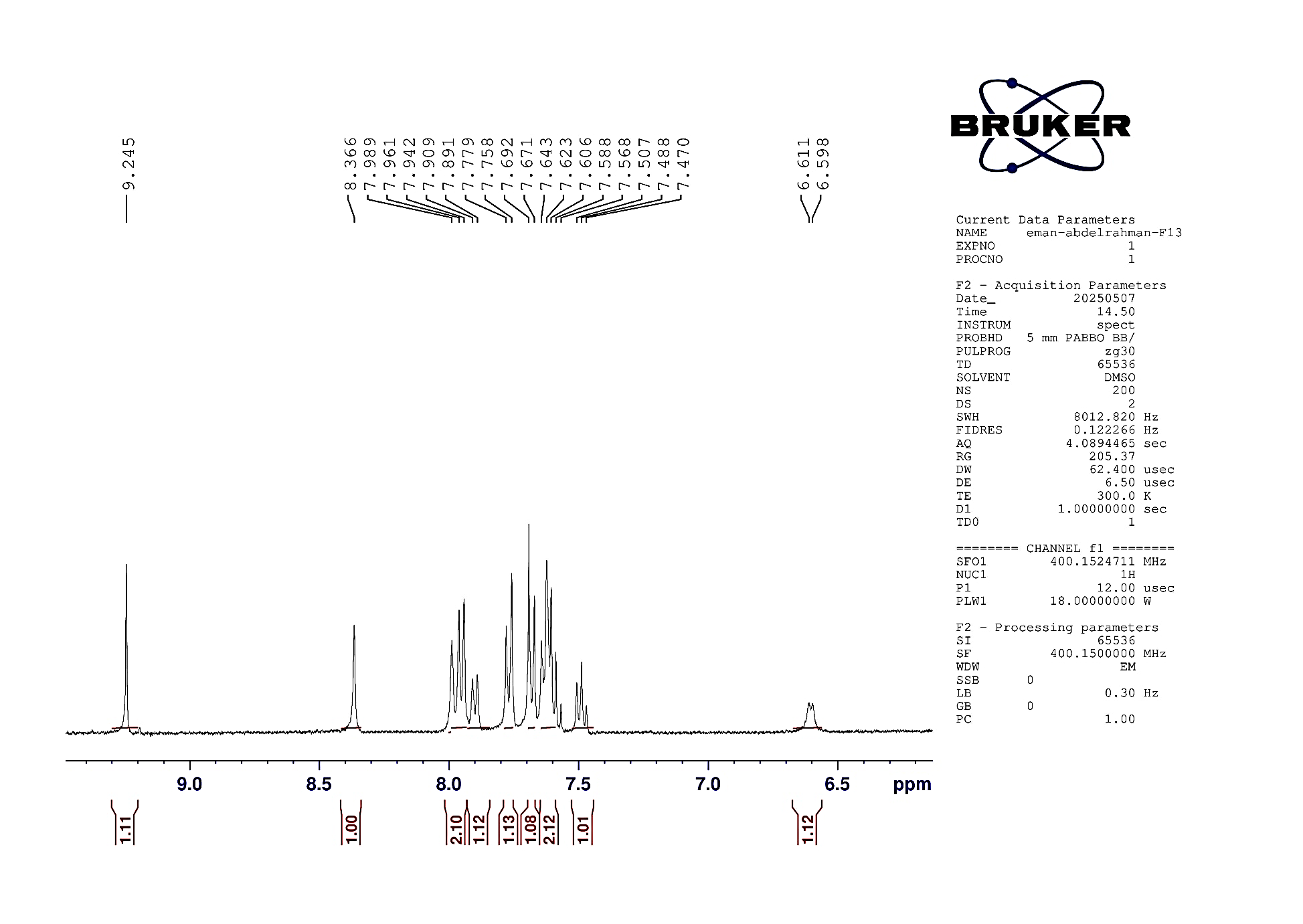


Cont. ^1^H NMR spectrum (DMSO-*d*_6_) of compound 4


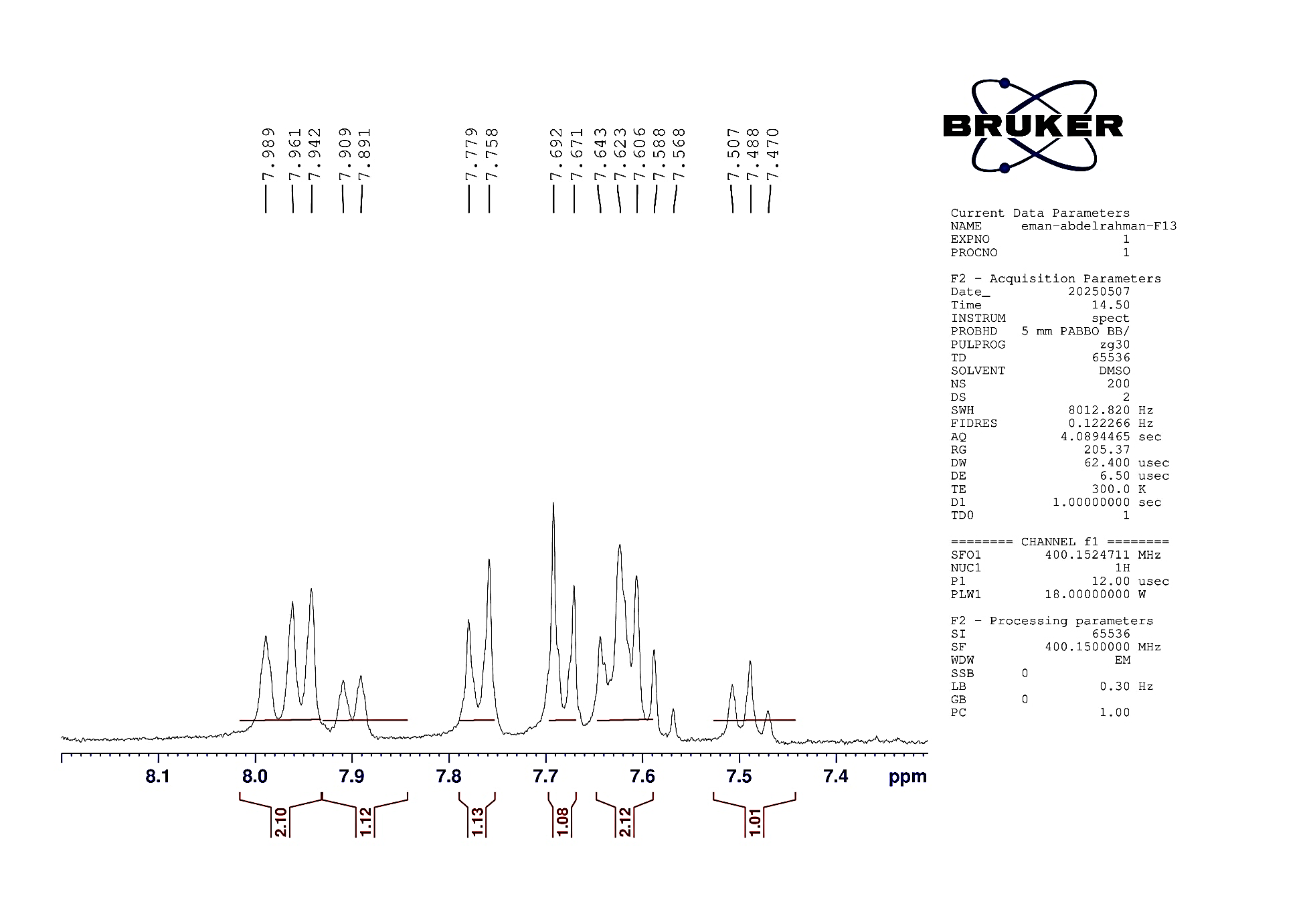


Cont. ^1^H NMR spectrum (DMSO-*d*_6_) of compound 4


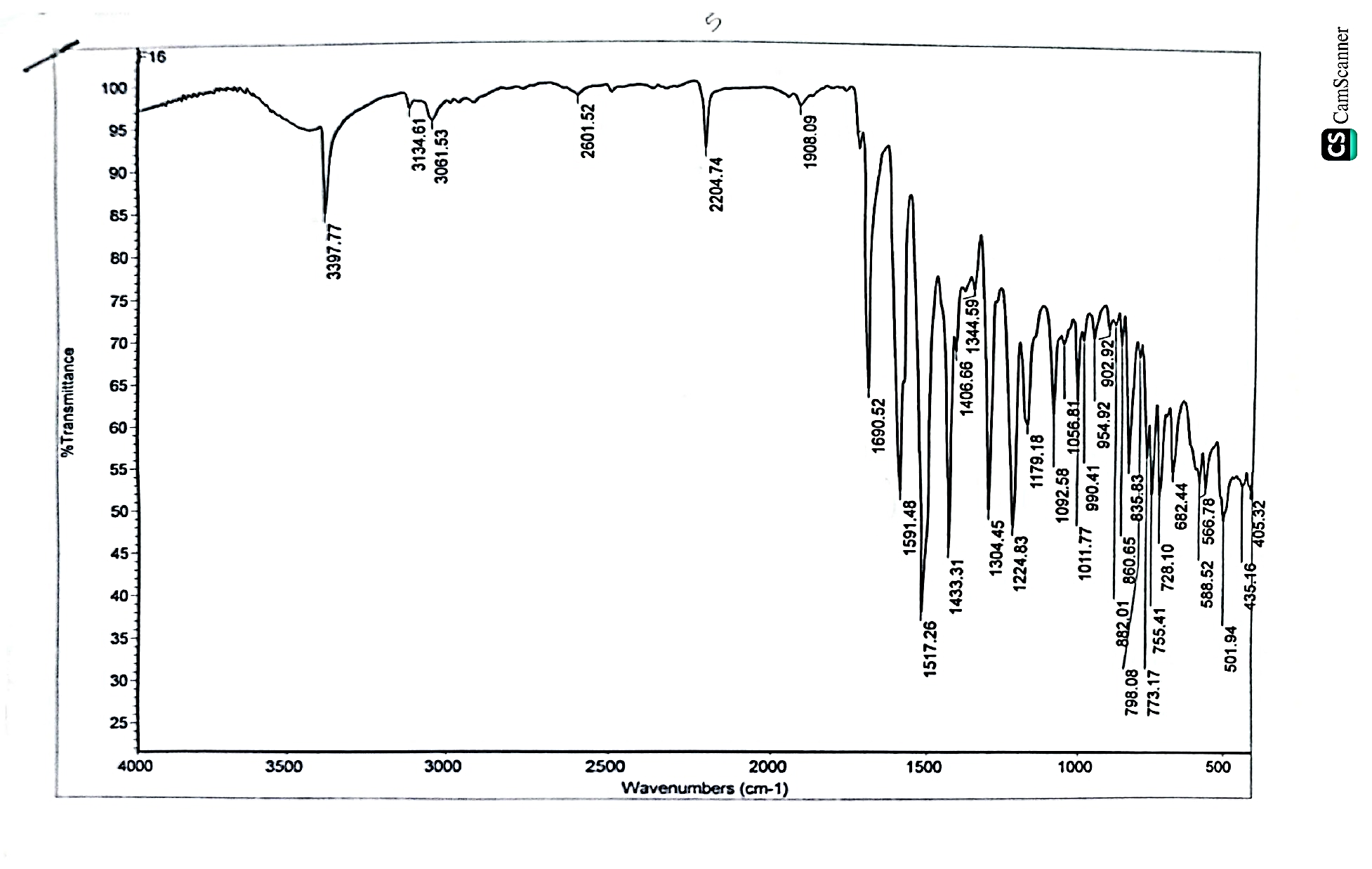


IR spectrum of compound 5


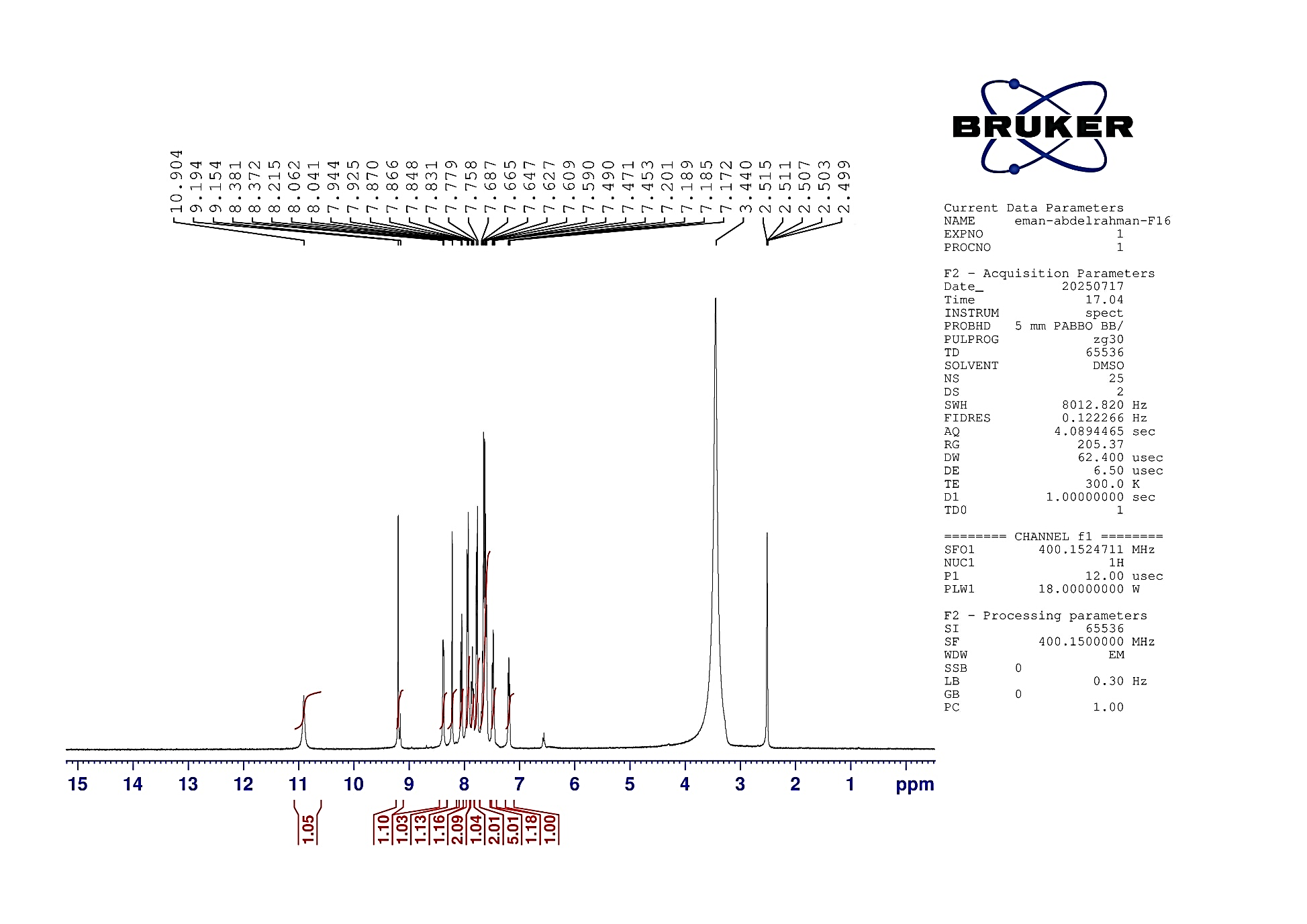


^1^H NMR spectrum (DMSO-*d*_6_) of compound 5


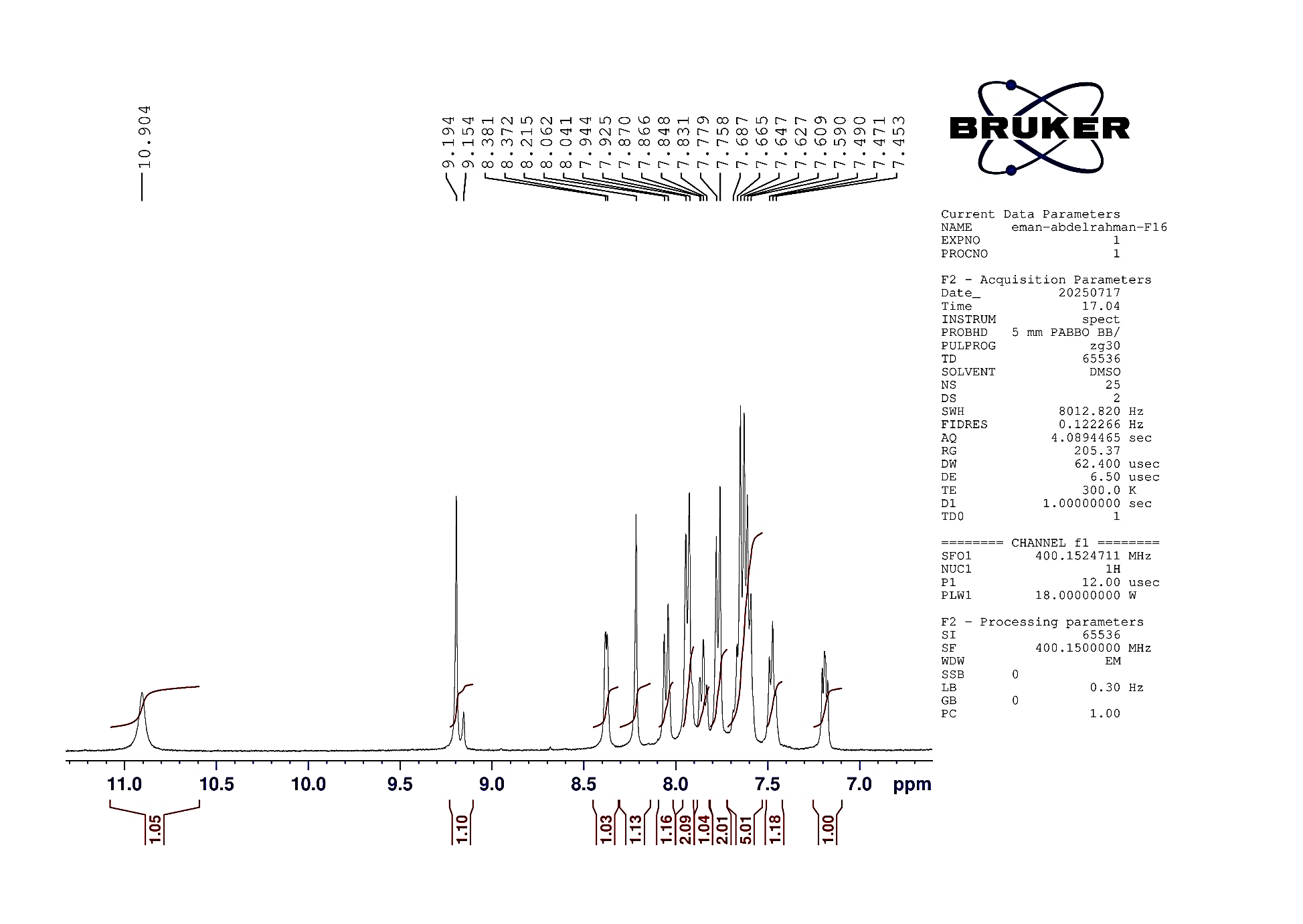


Cont. ^1^H NMR spectrum (DMSO-*d*_6_) of compound 5


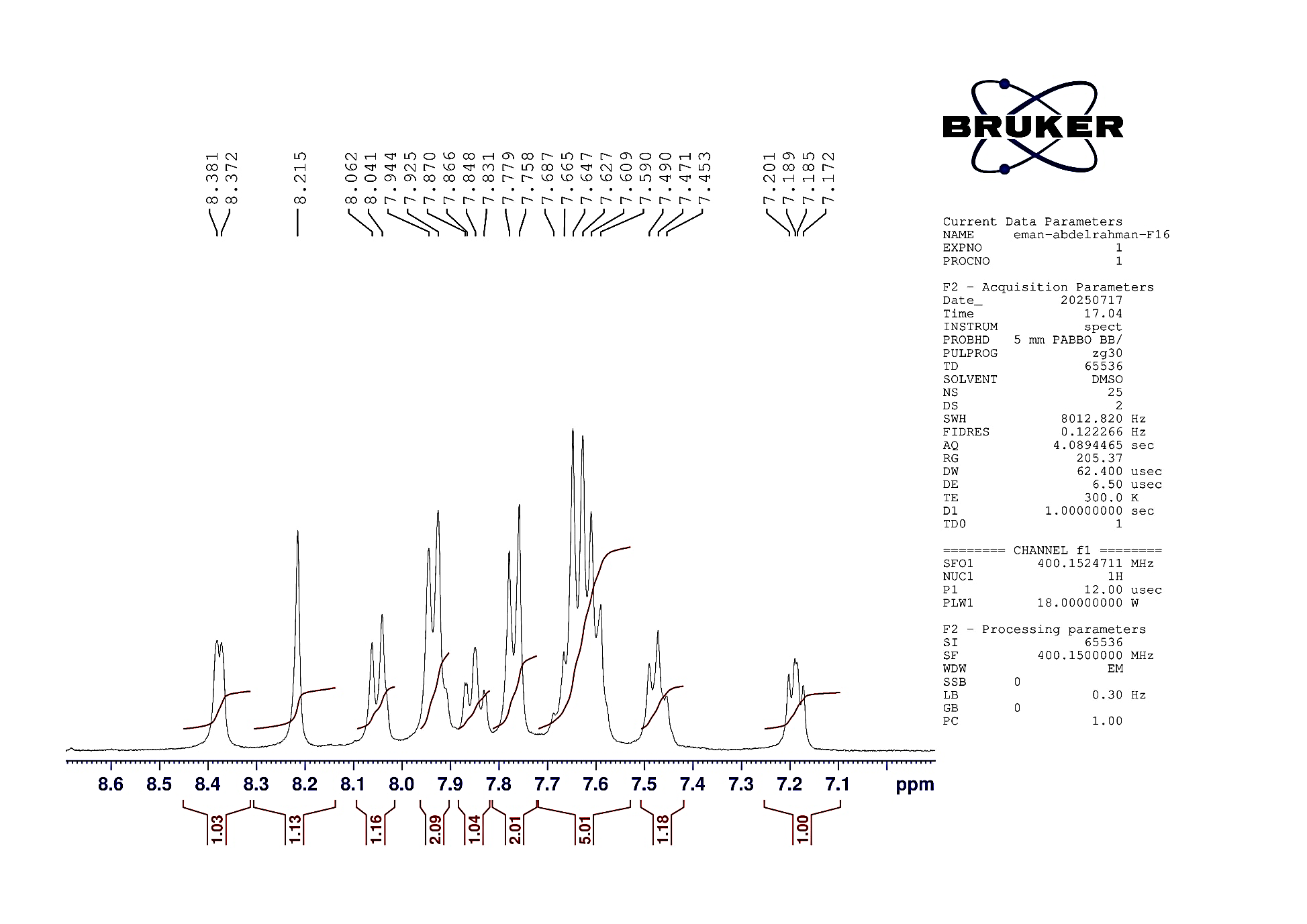


Cont. ^1^H NMR spectrum (DMSO-*d*_6_) of compound 5


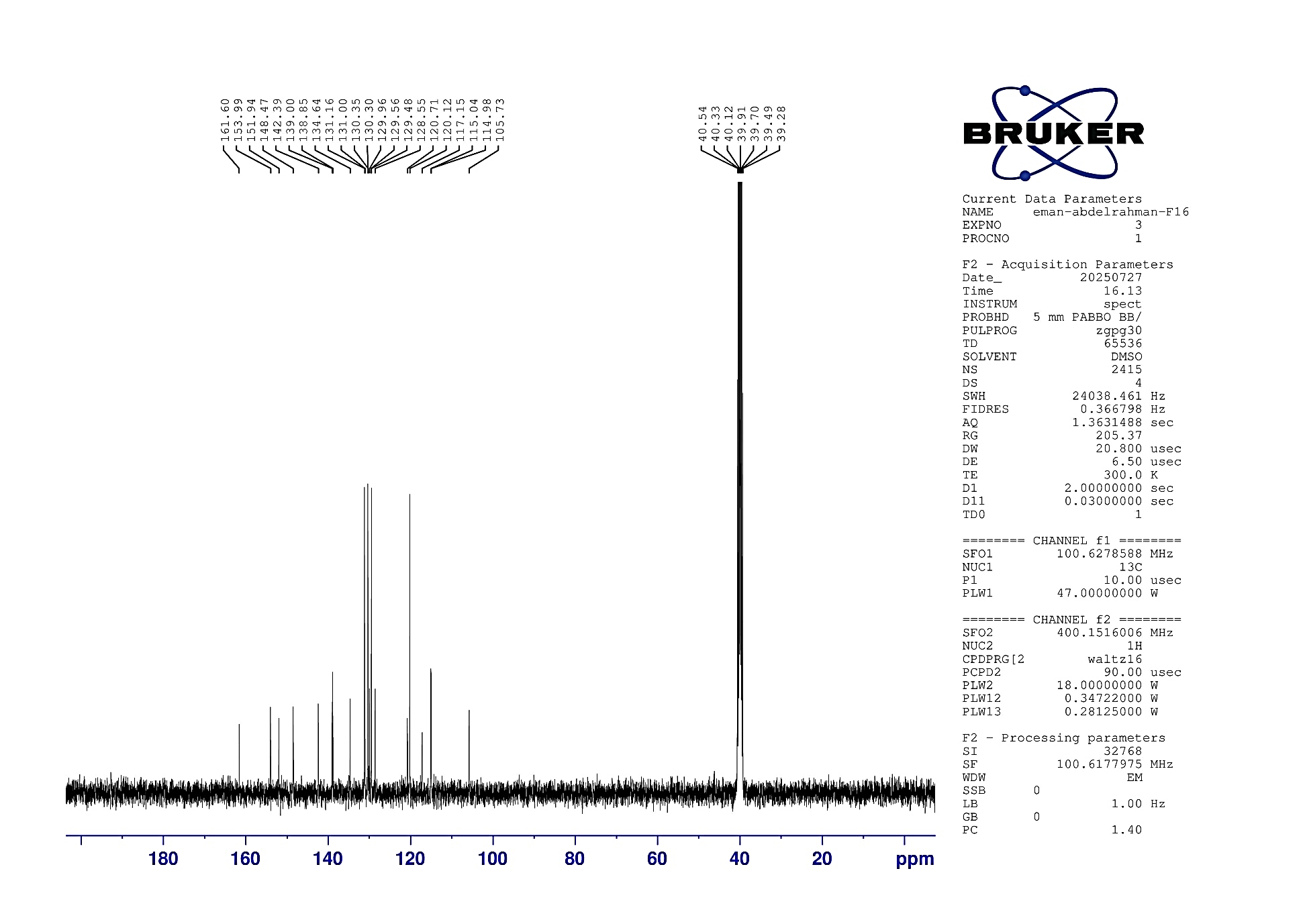


^13^C NMR spectrum (DMSO-*d*_6_) of compound 5


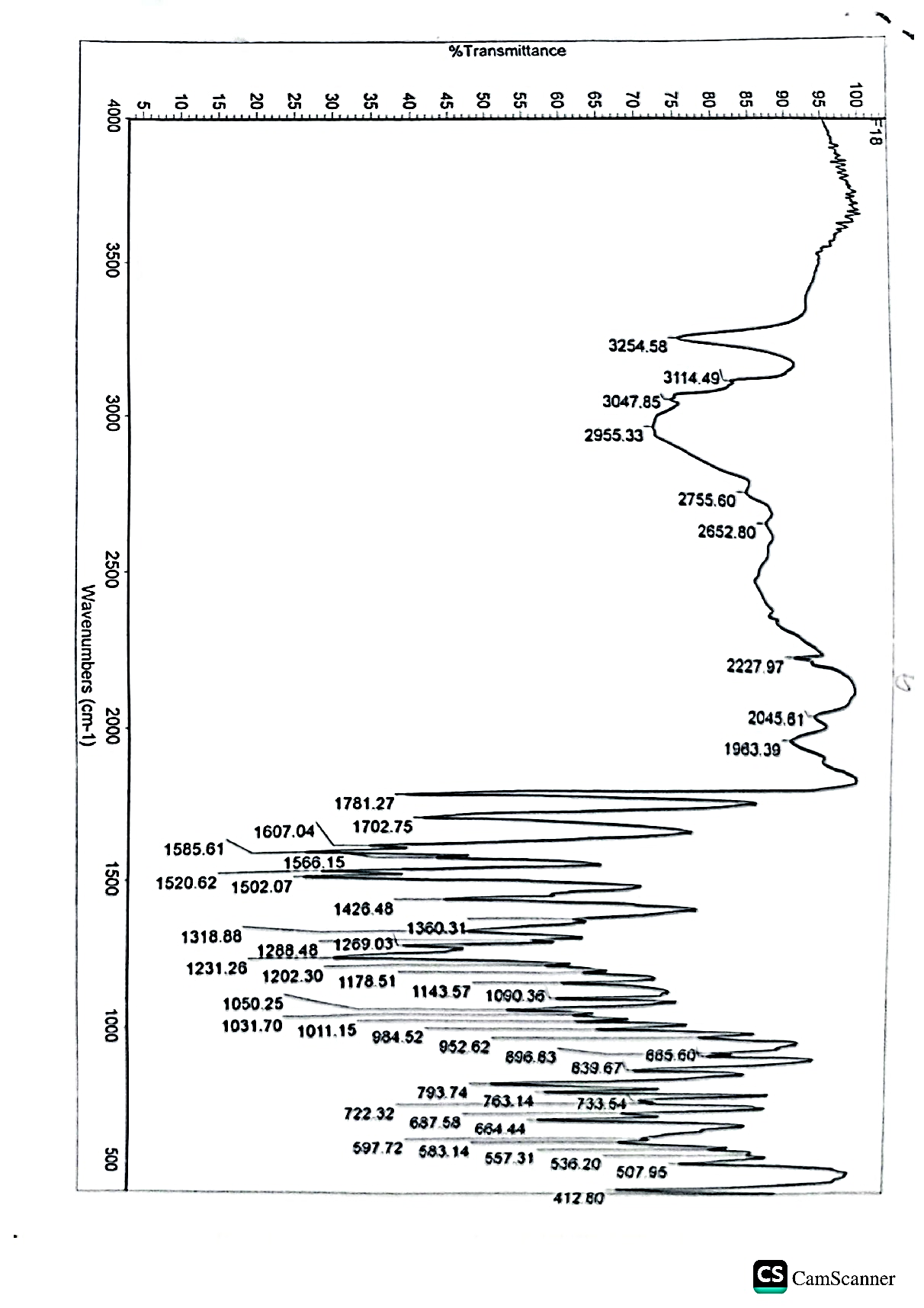


IR spectrum of compound 6


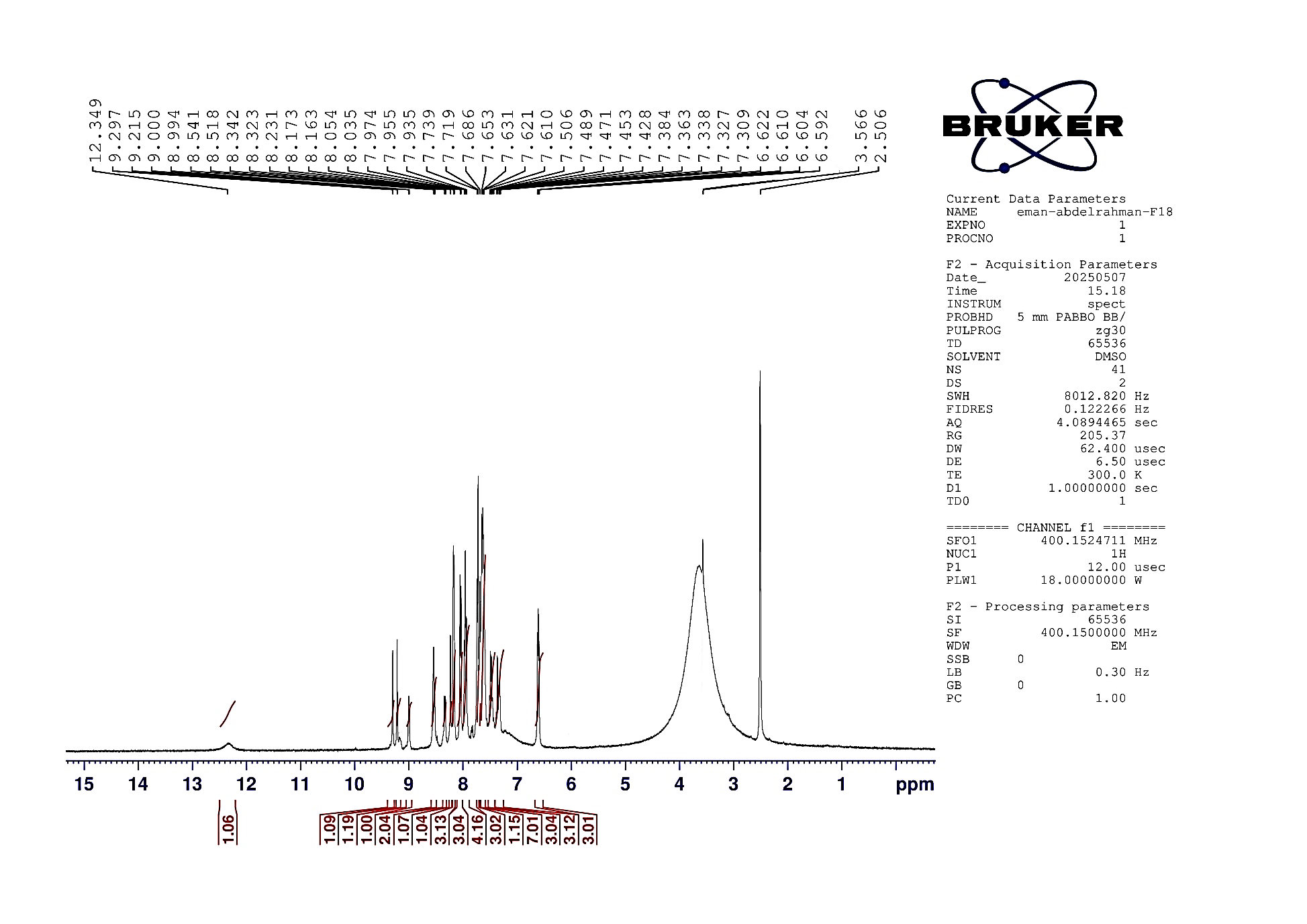


^1^H NMR spectrum (DMSO-*d*_6_) of compound 6


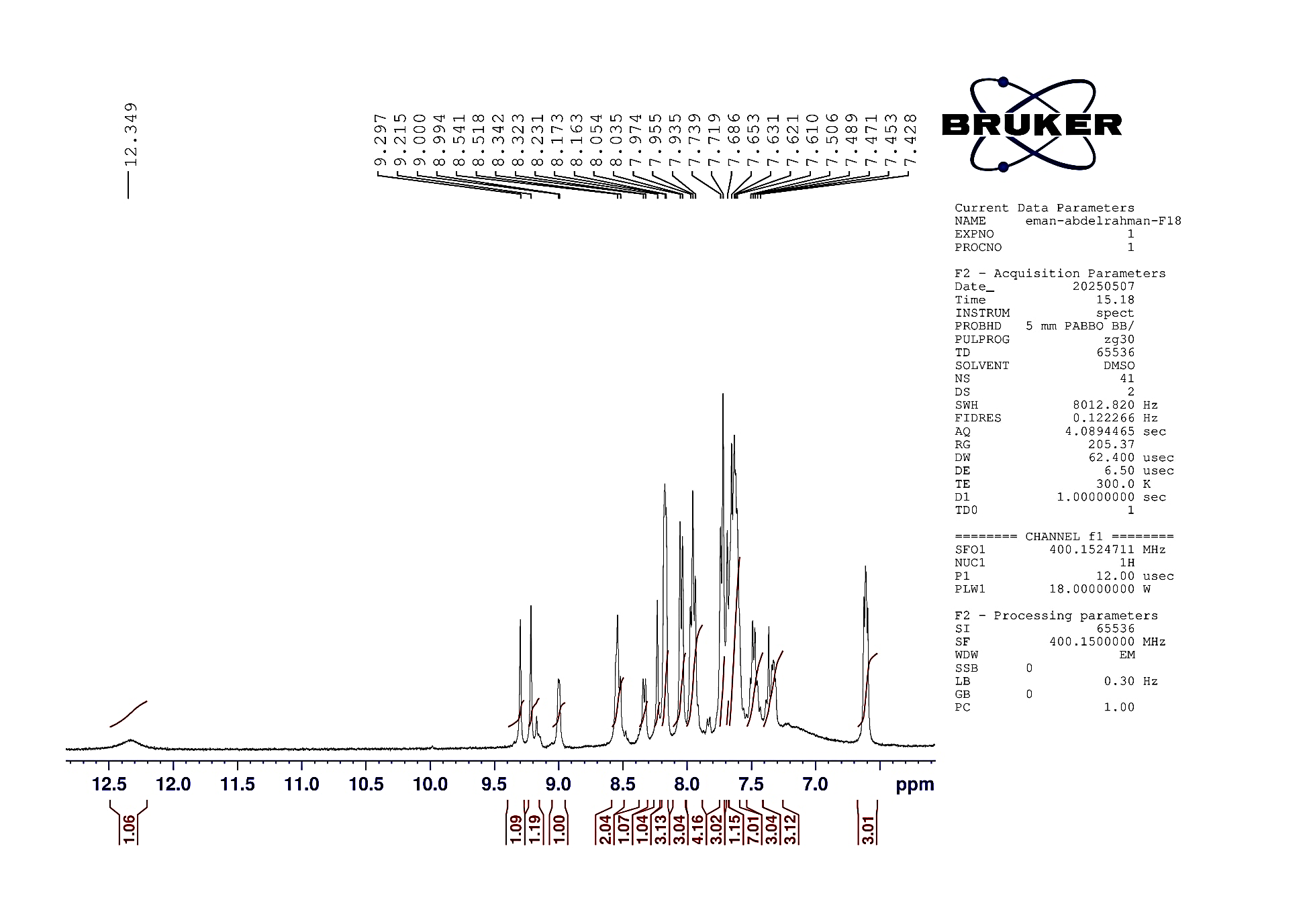


Cont. ^1^H NMR spectrum (DMSO-*d*_6_) of compound 6


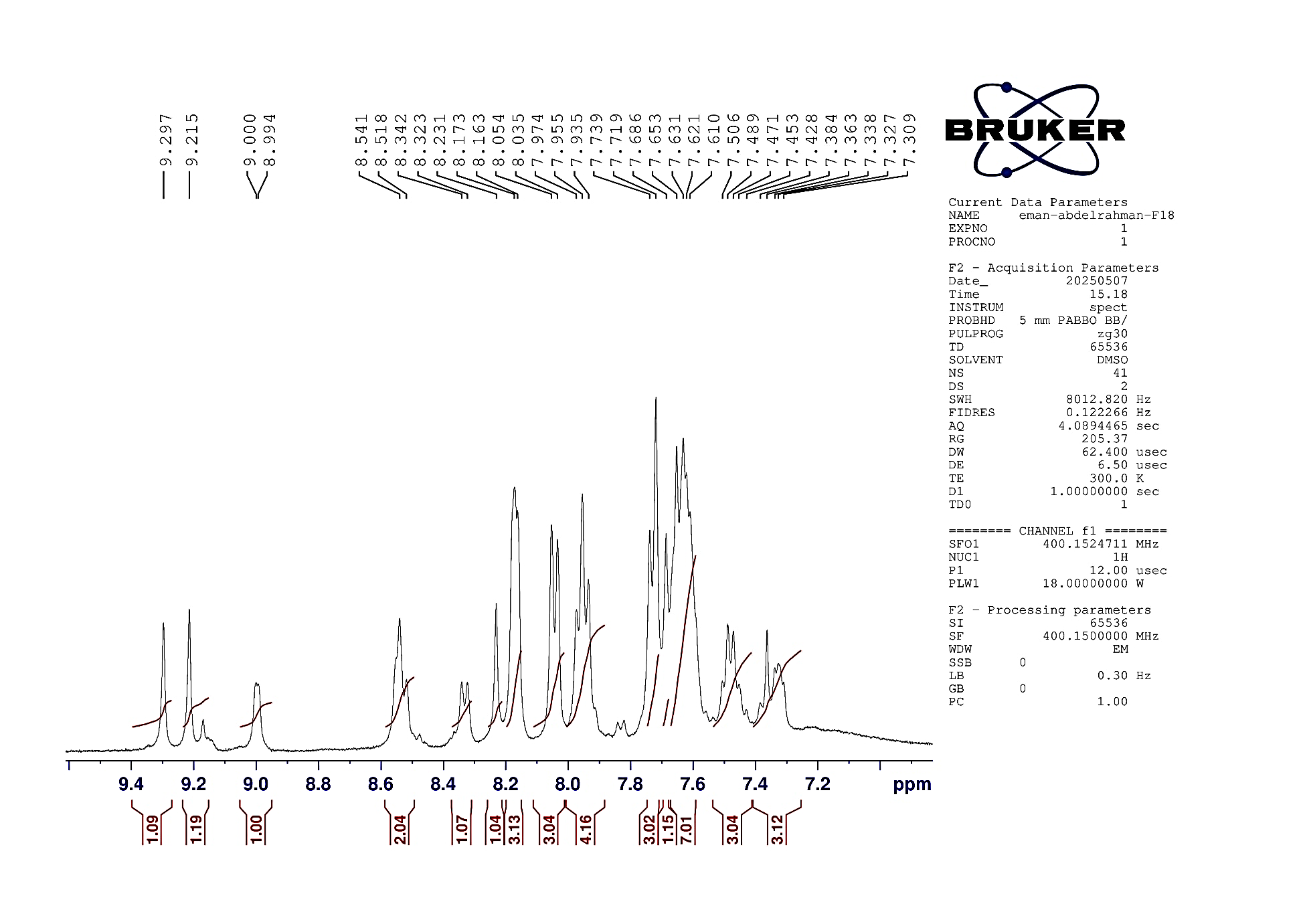


Cont. ^1^H NMR spectrum (DMSO-*d*_6_) of compound 6


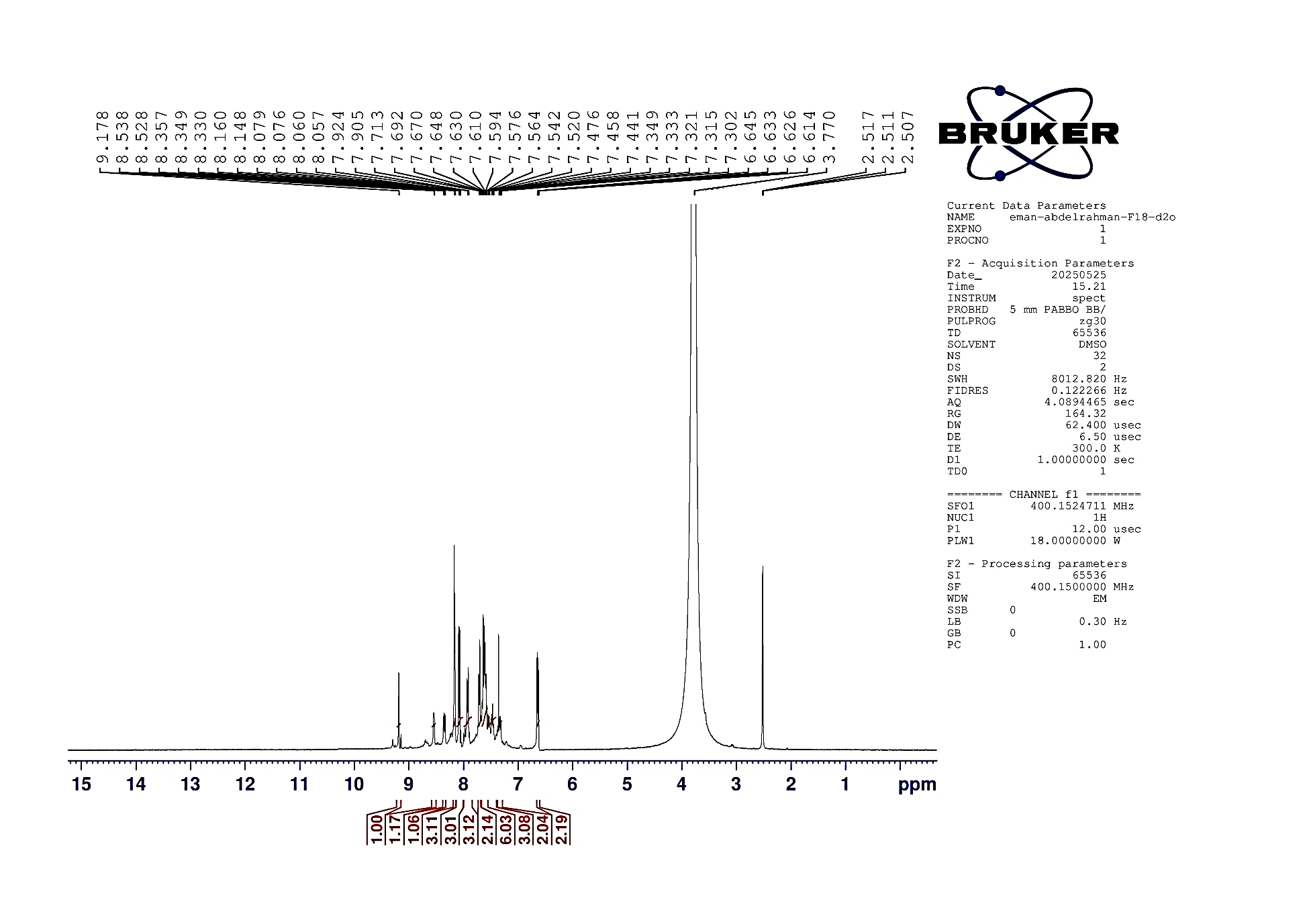


^1^H NMR spectrum (DMSO-*d*_6_+D_2_O) of compound 6


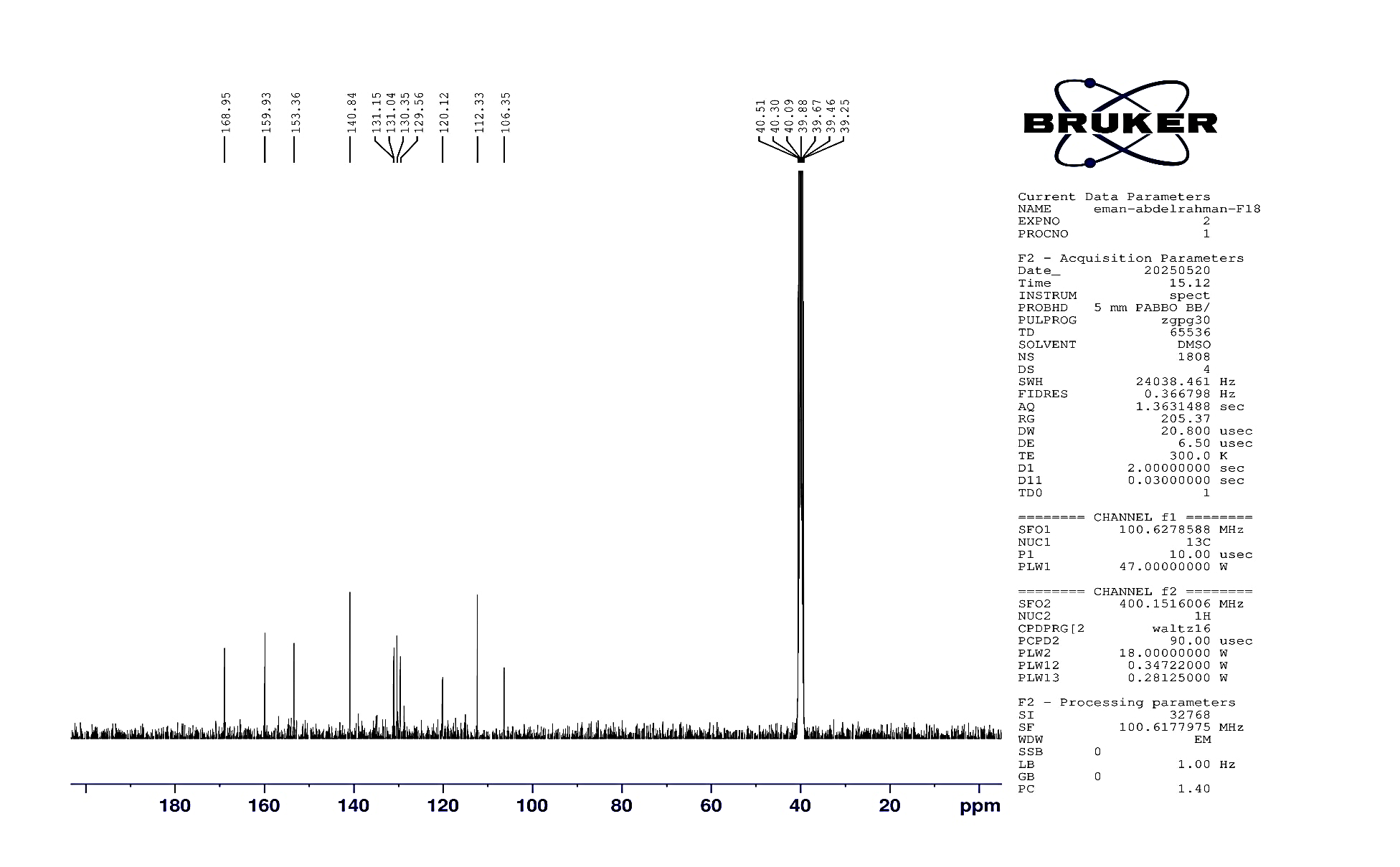


^13^C NMR spectrum (DMSO-*d*_6_) of compound 6


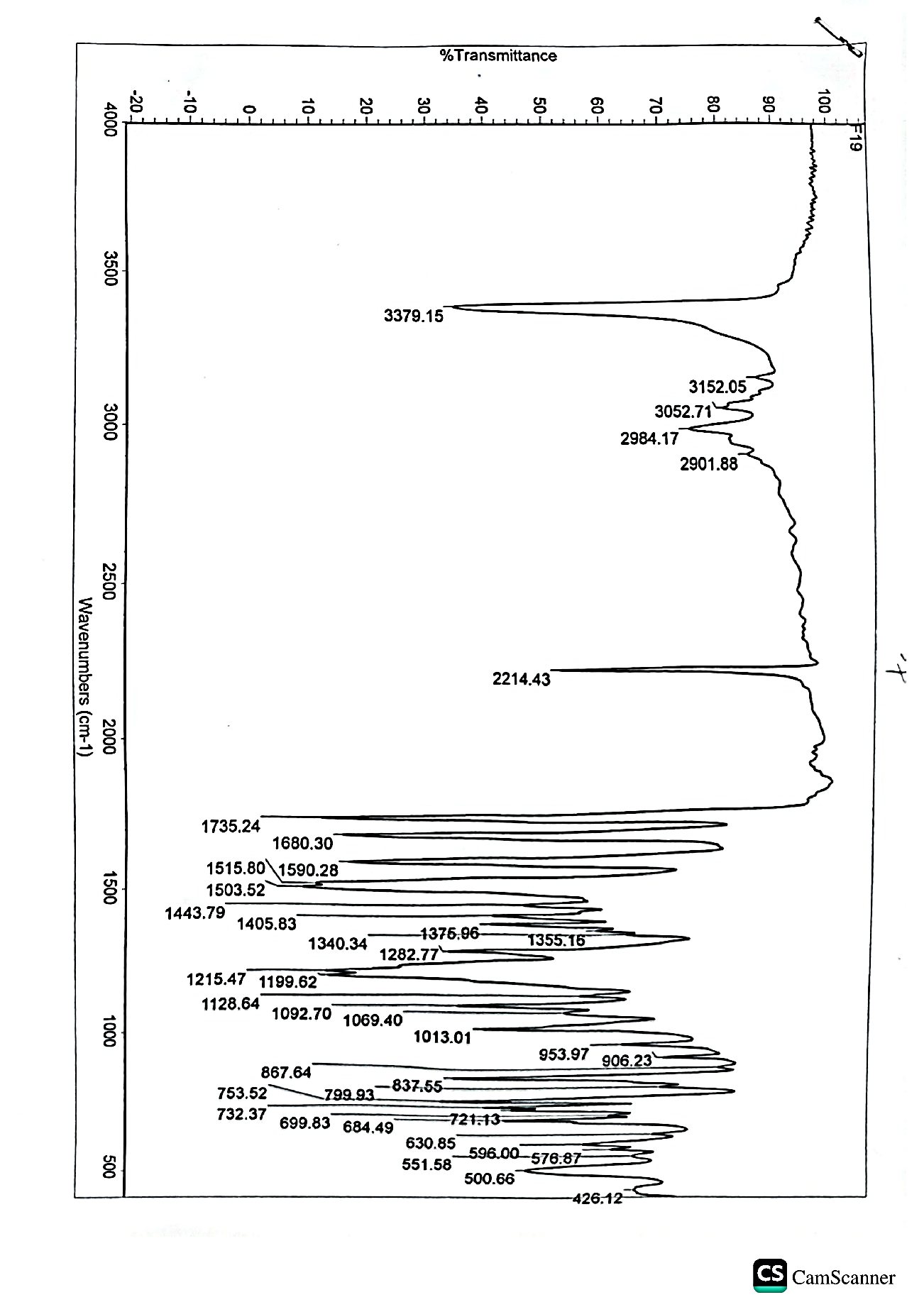


IR spectrum of compound 7


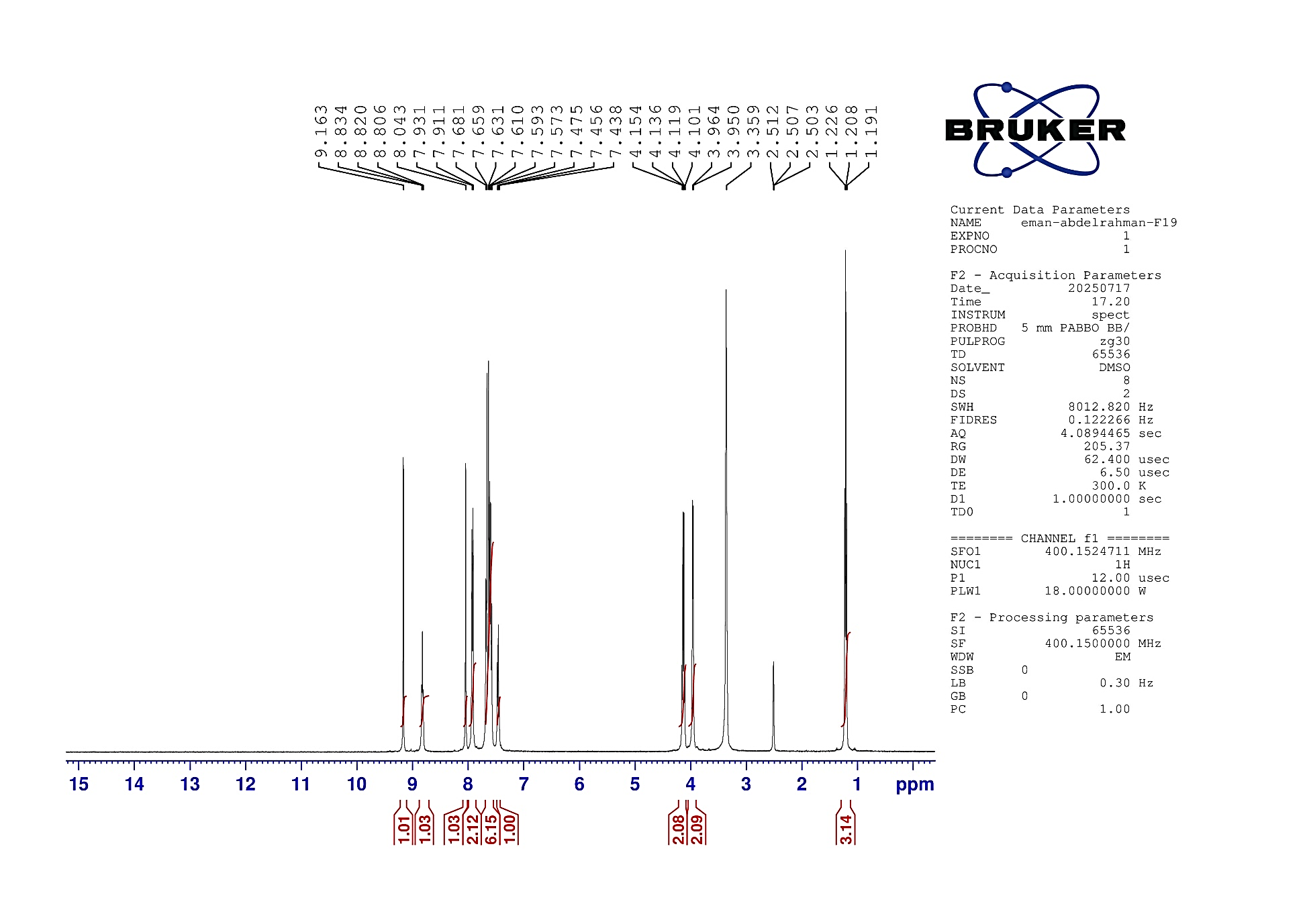


^1^H NMR spectrum (DMSO-*d*_6_) of compound 7


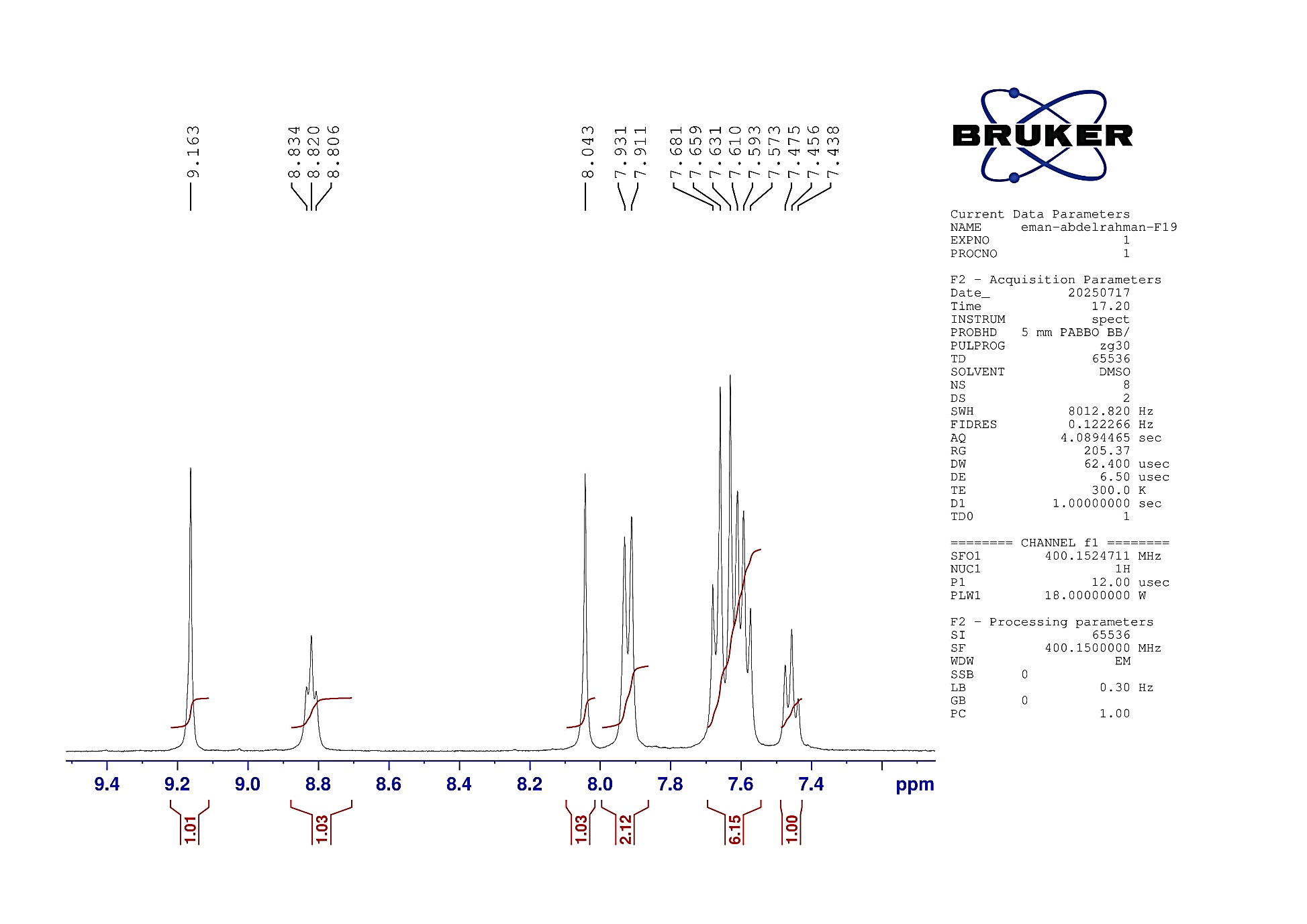


^1^H NMR spectrum (DMSO-*d*_6_) of compound 7


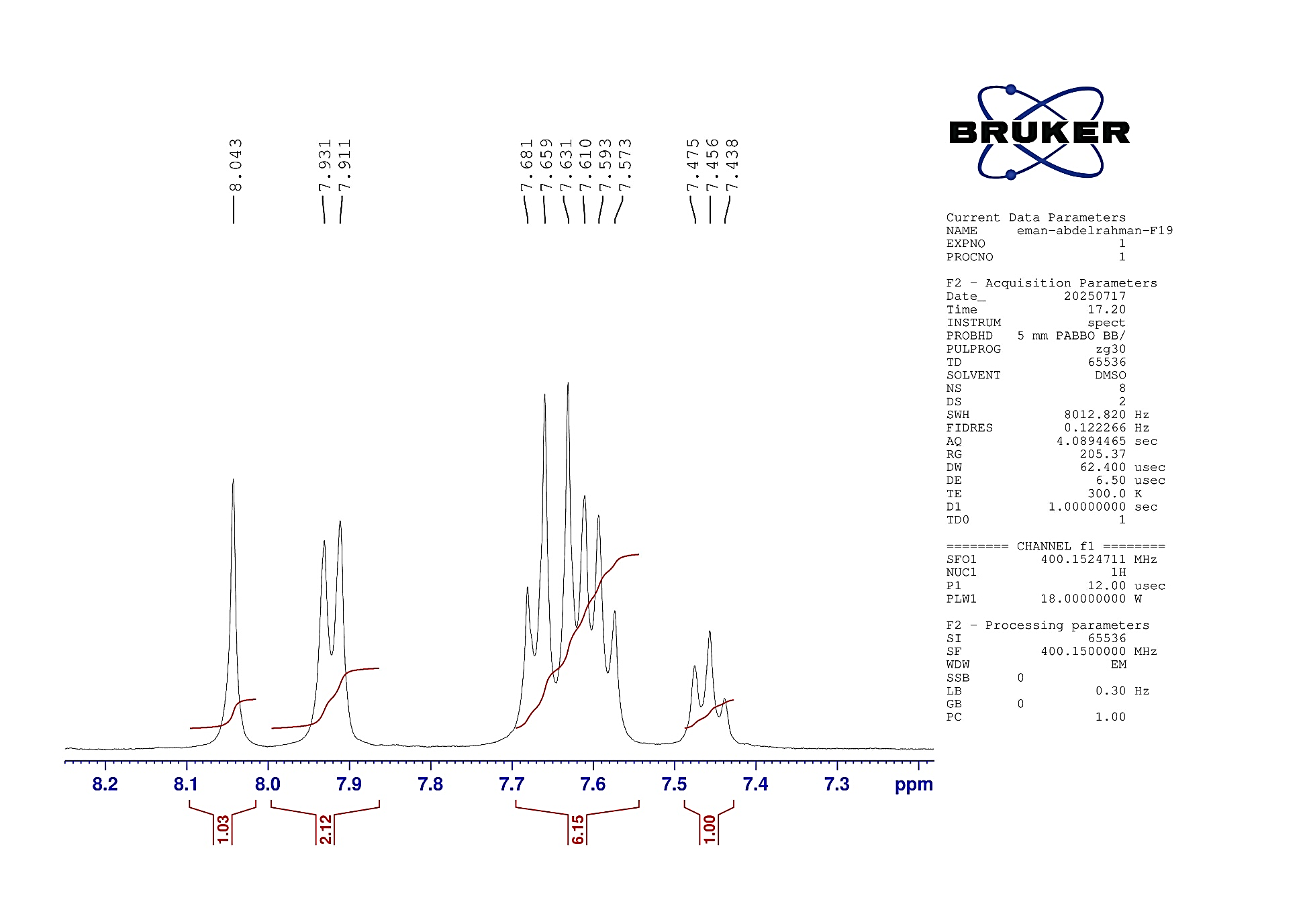


Cont. ^1^H NMR spectrum (DMSO-*d*_6_) of compound 7


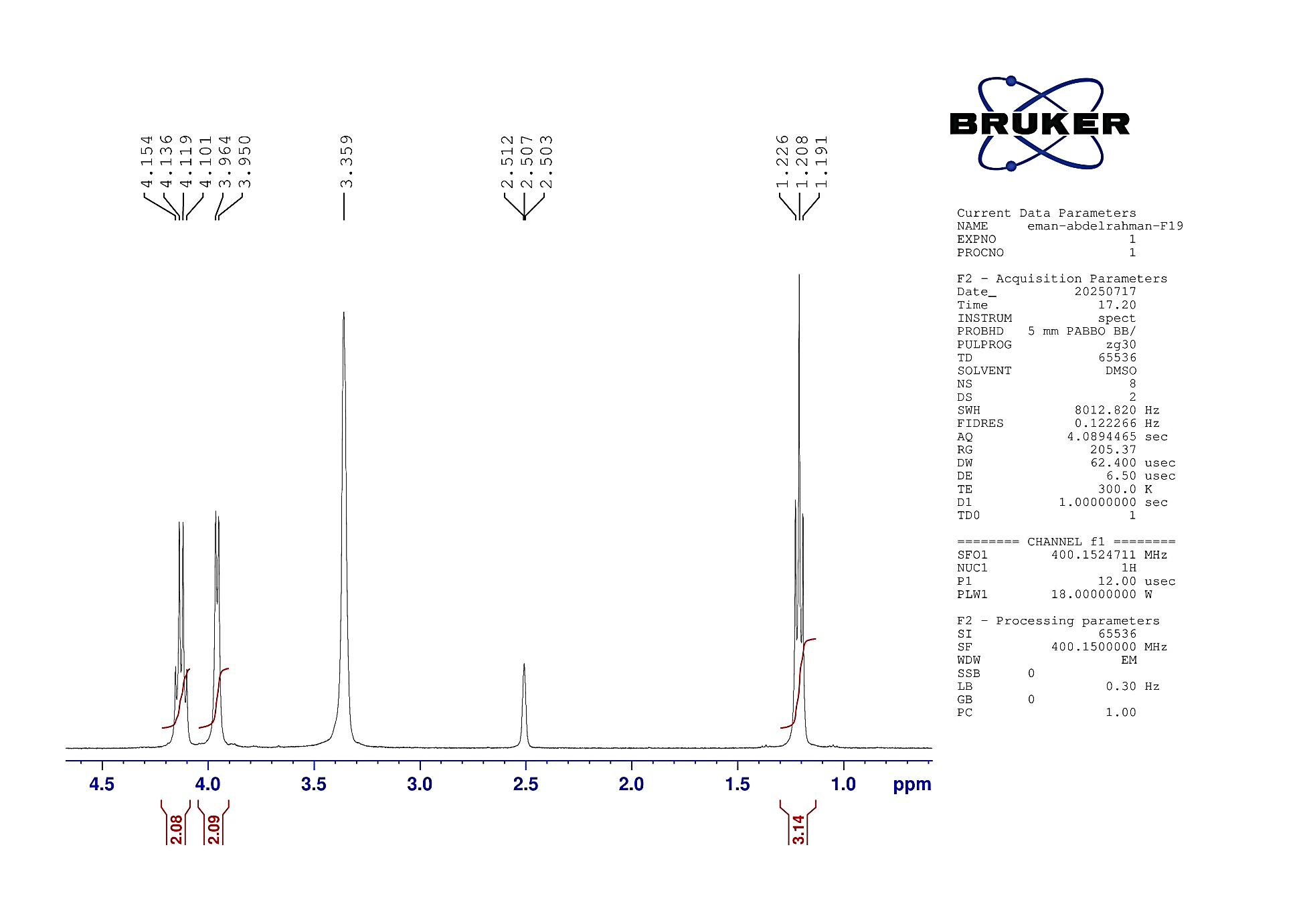


Cont. ^1^H NMR spectrum (DMSO-*d*_6_) of compound 7


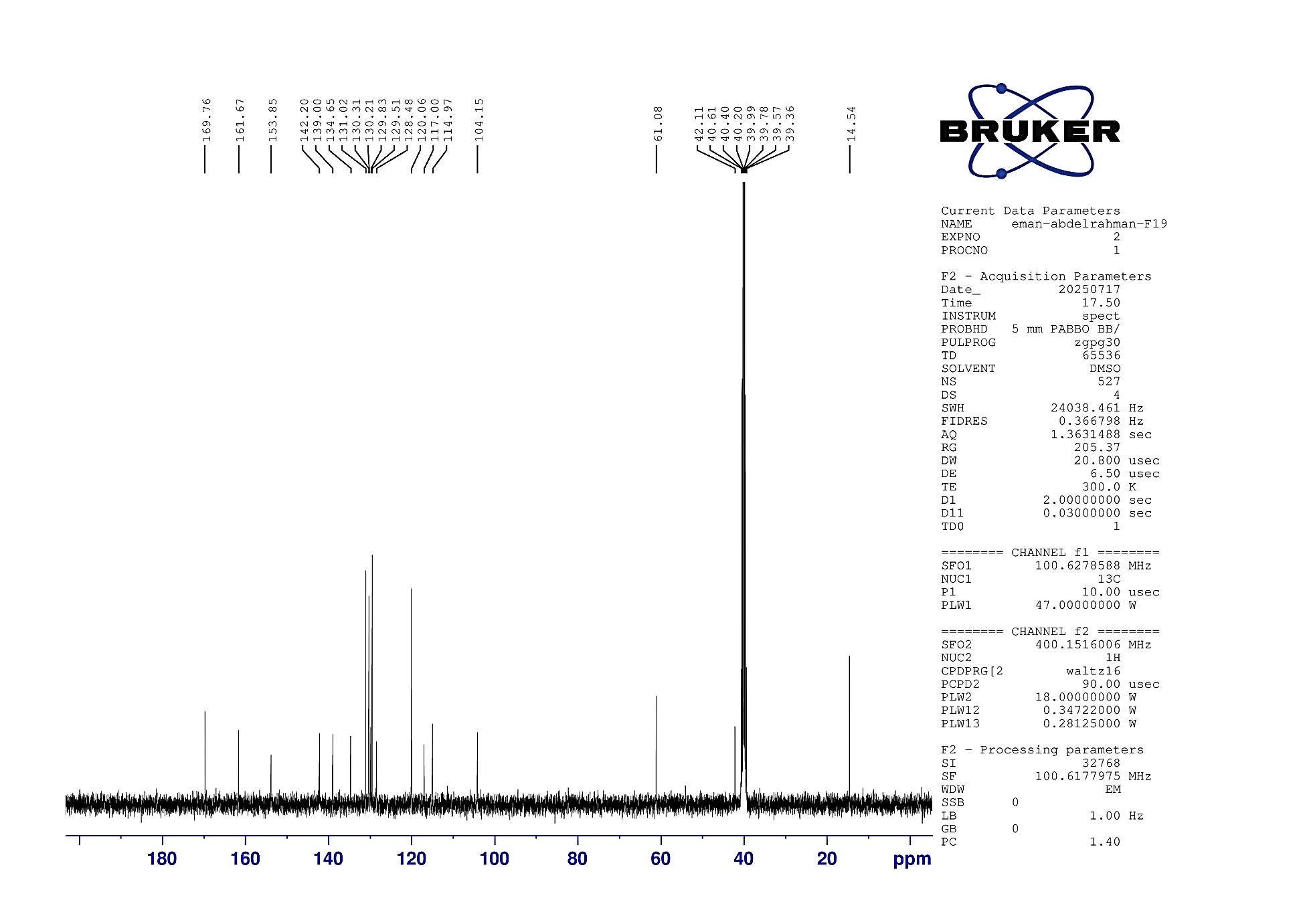


^13^C NMR spectrum (DMSO-*d*_6_) of compound 7


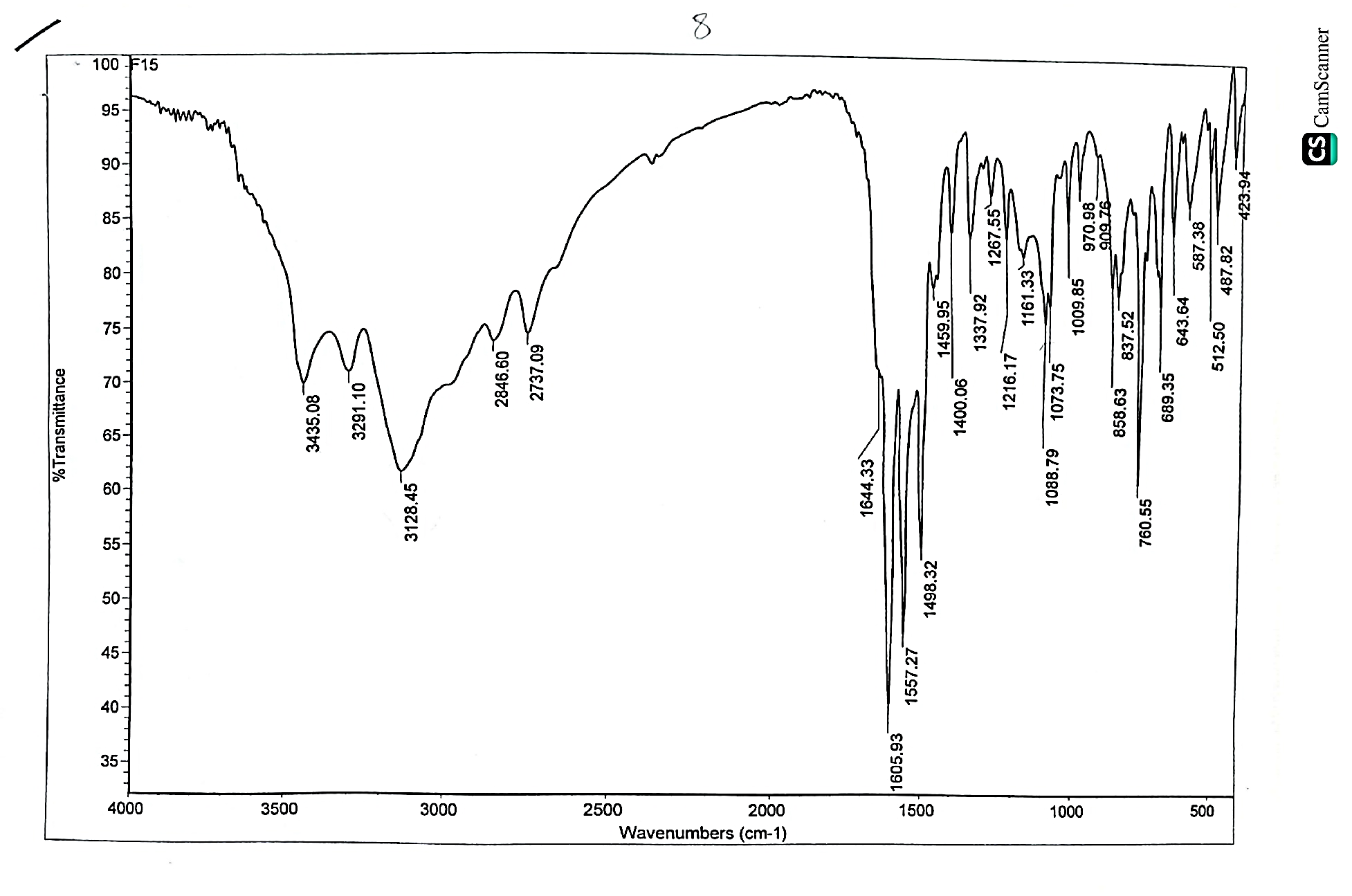


IR spectrum of compound 8


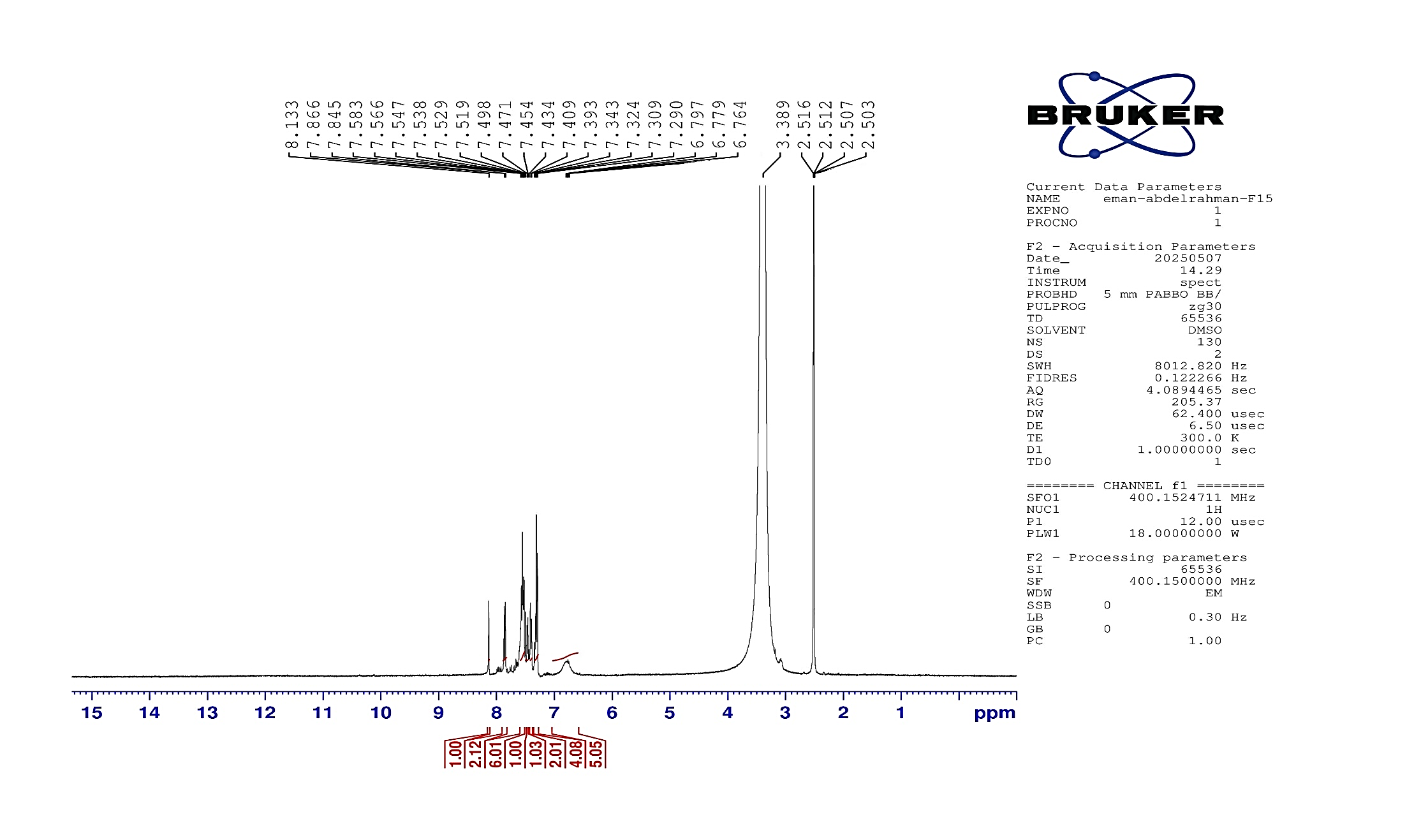


^1^H NMR spectrum (DMSO-*d*_6_) of compound **8**


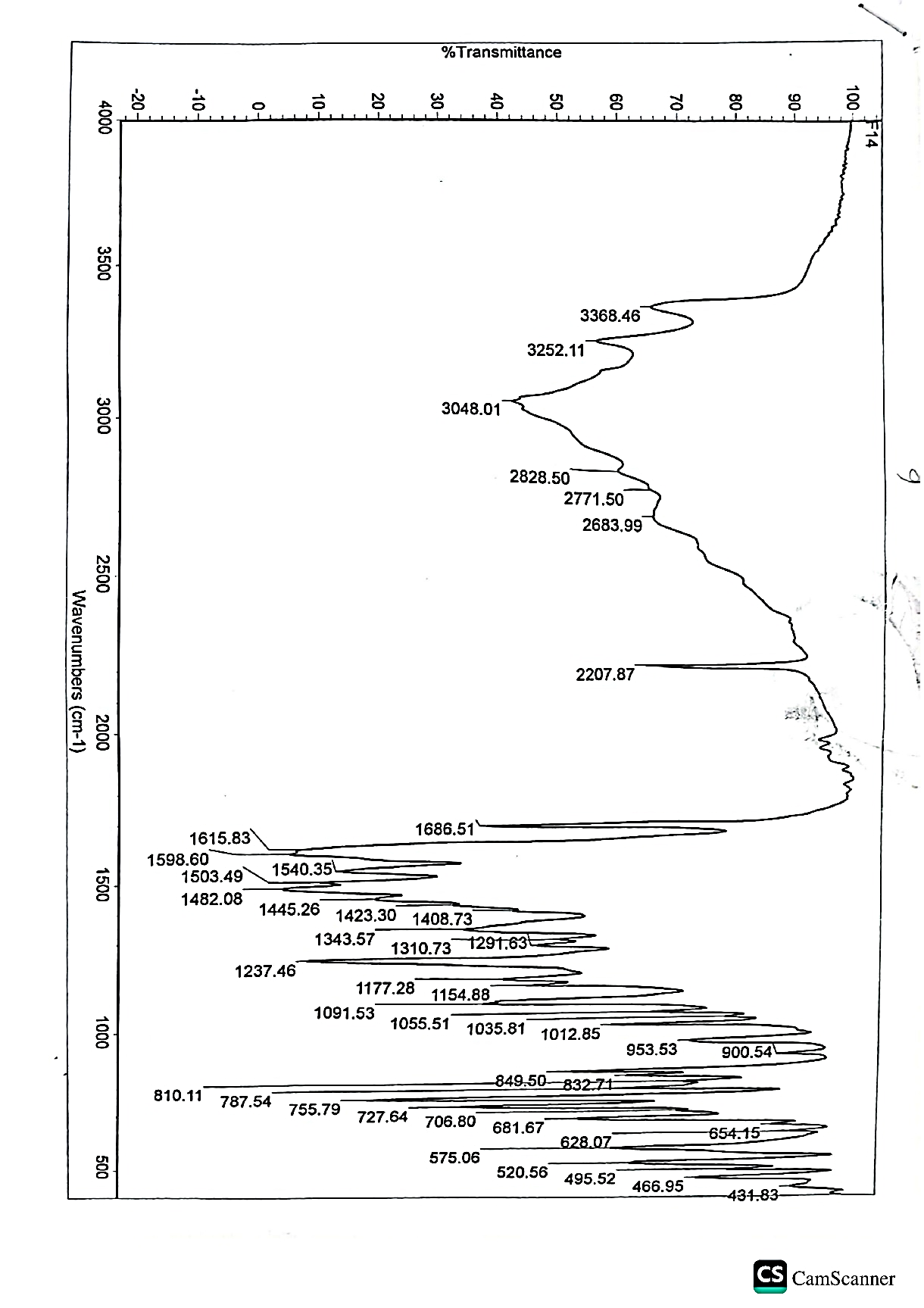


IR spectrum of compound 9


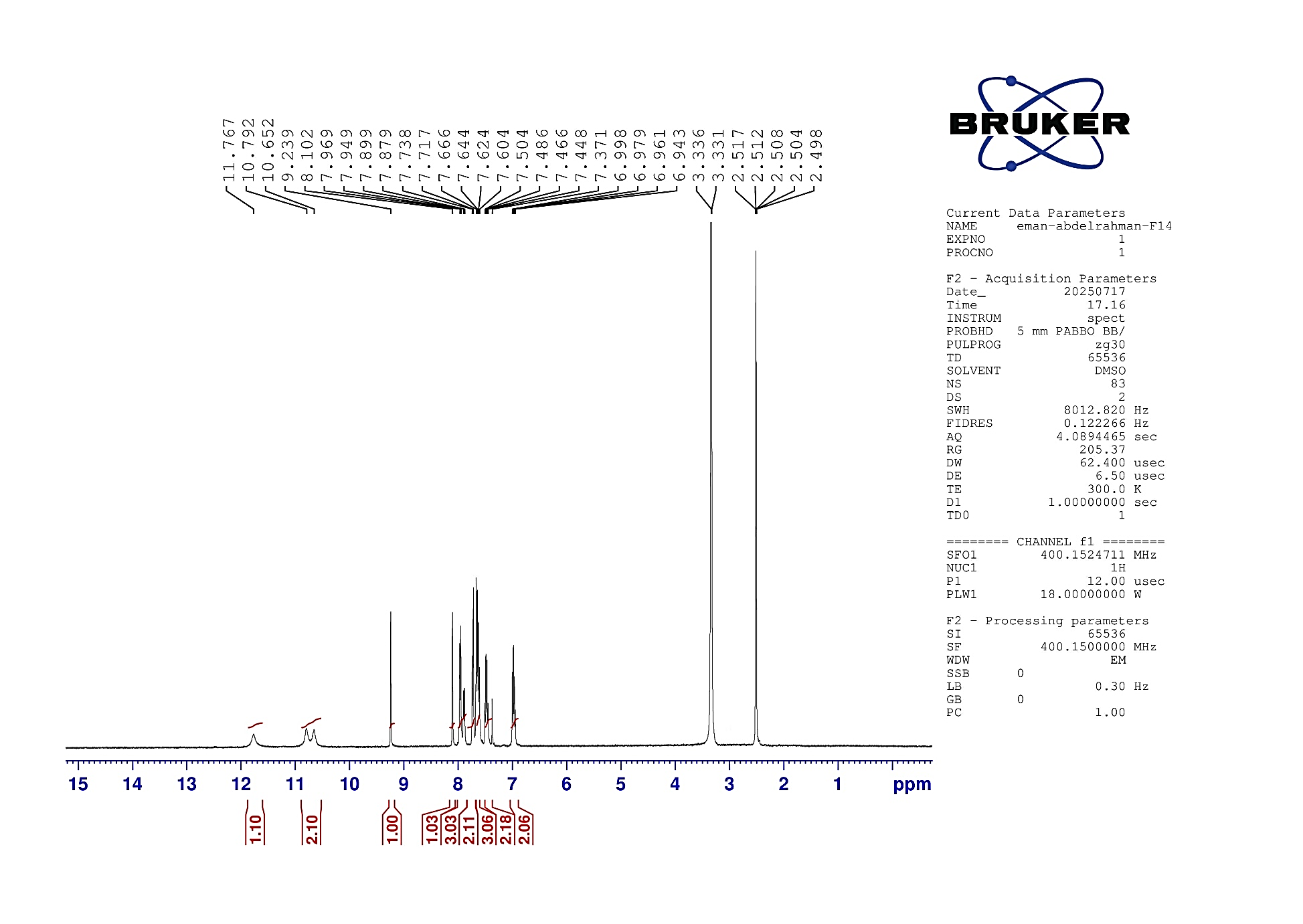


^1^H NMR spectrum (DMSO-*d*_6_) of compound **9**


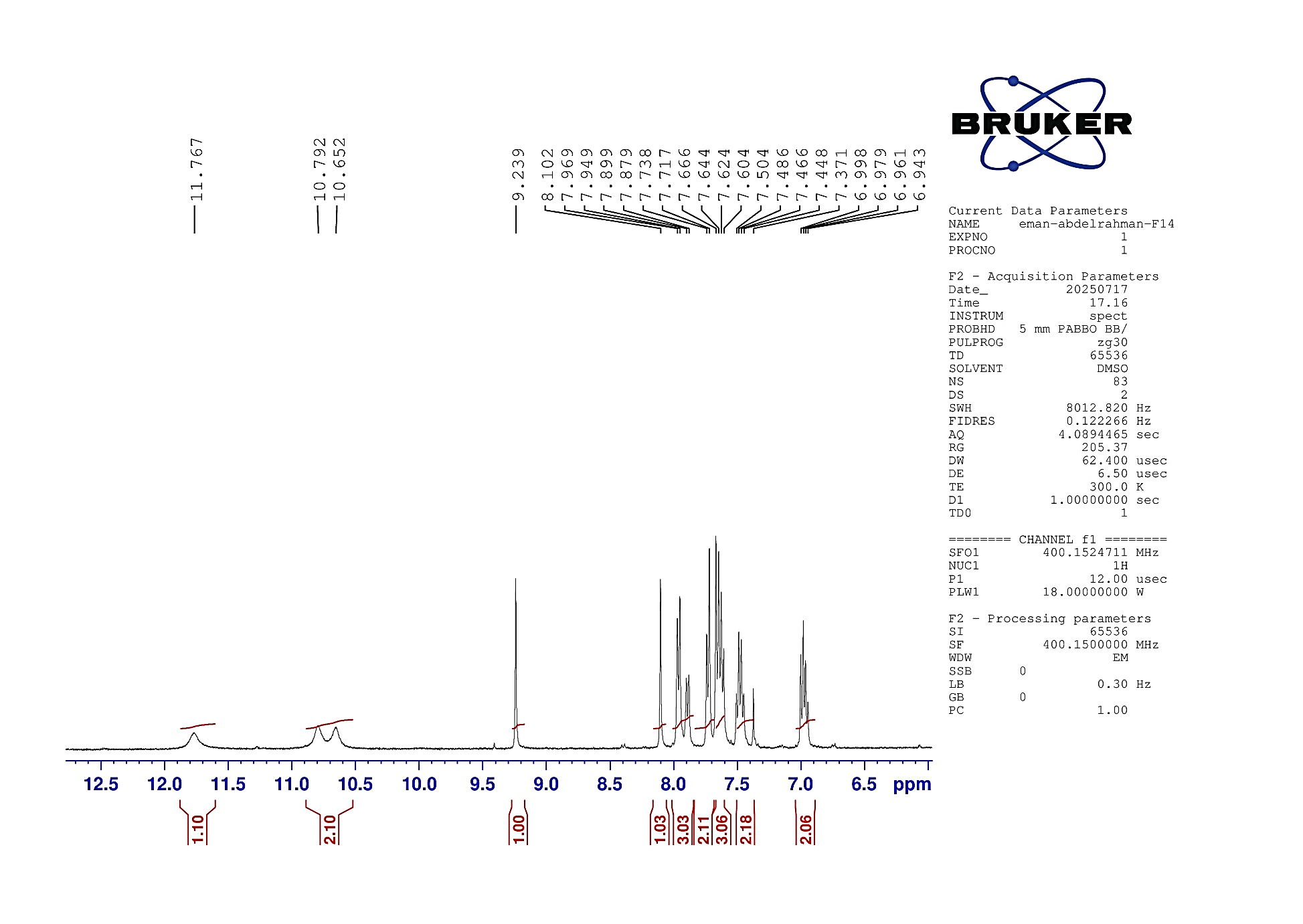


Cont. ^1^H NMR spectrum (DMSO-*d*_6_) of compound **9**


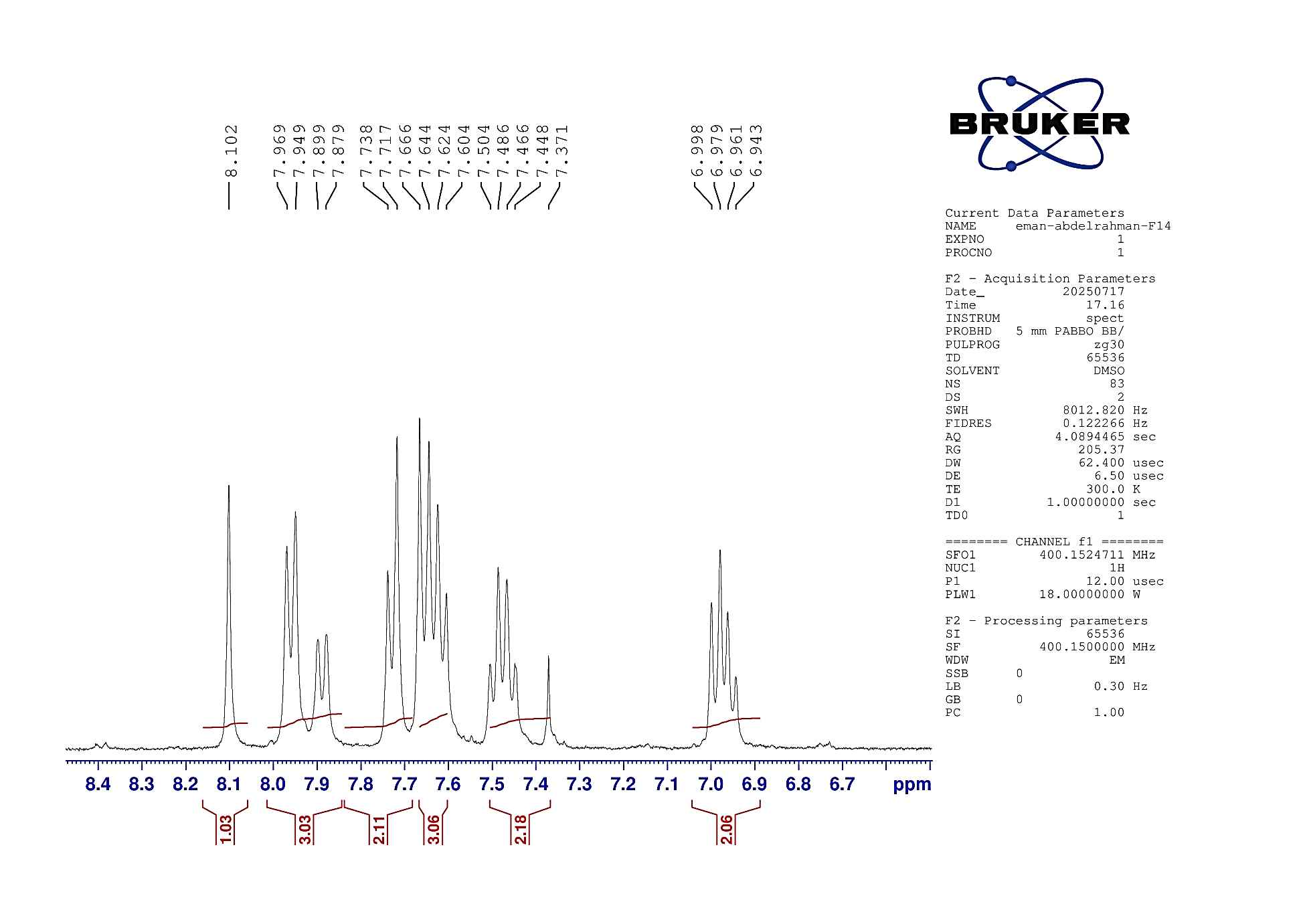


^1^H NMR spectrum (DMSO-*d*_6_) of compound **9**


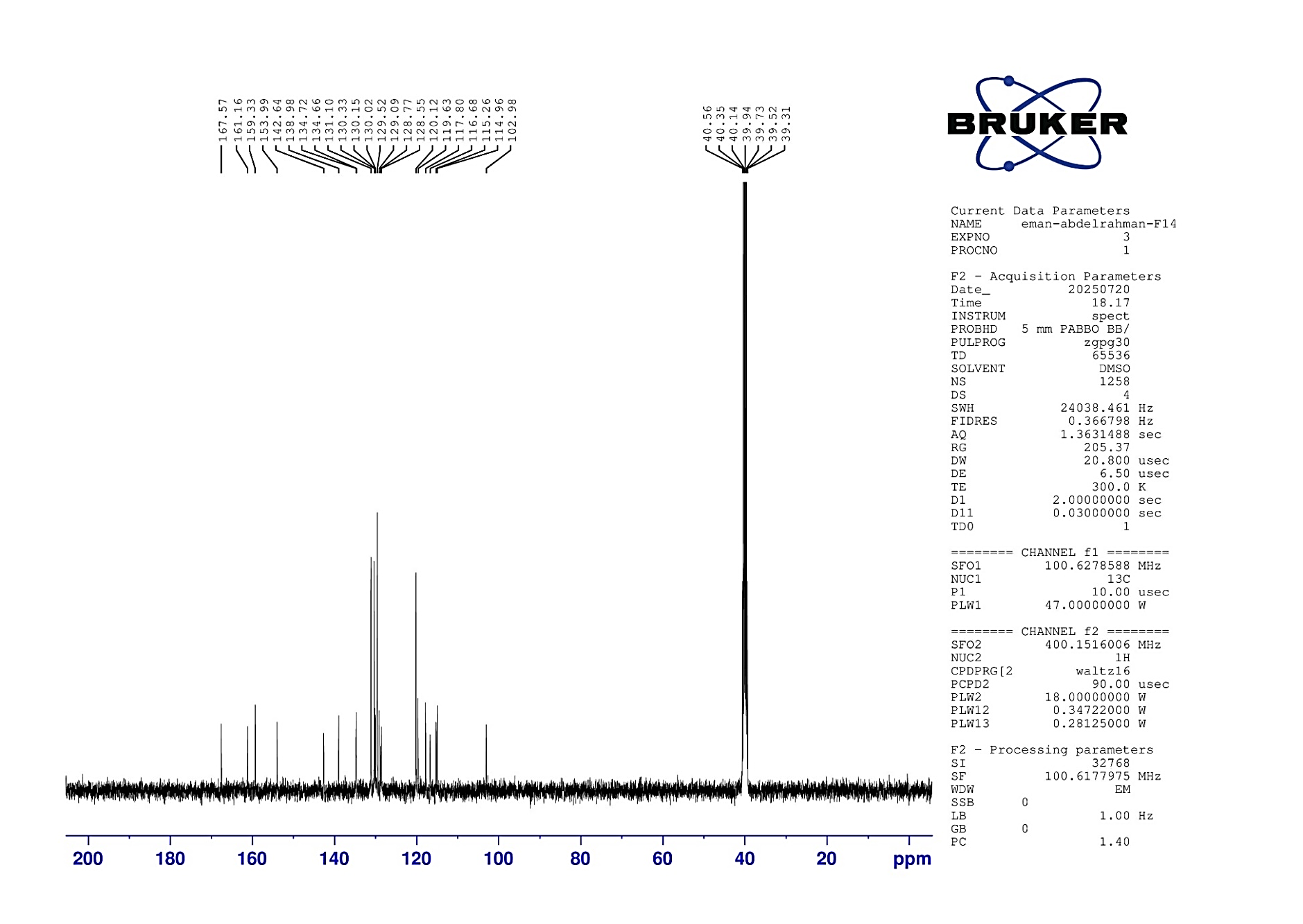


^13^C NMR spectrum (DMSO-*d*_6_) of compound **9**


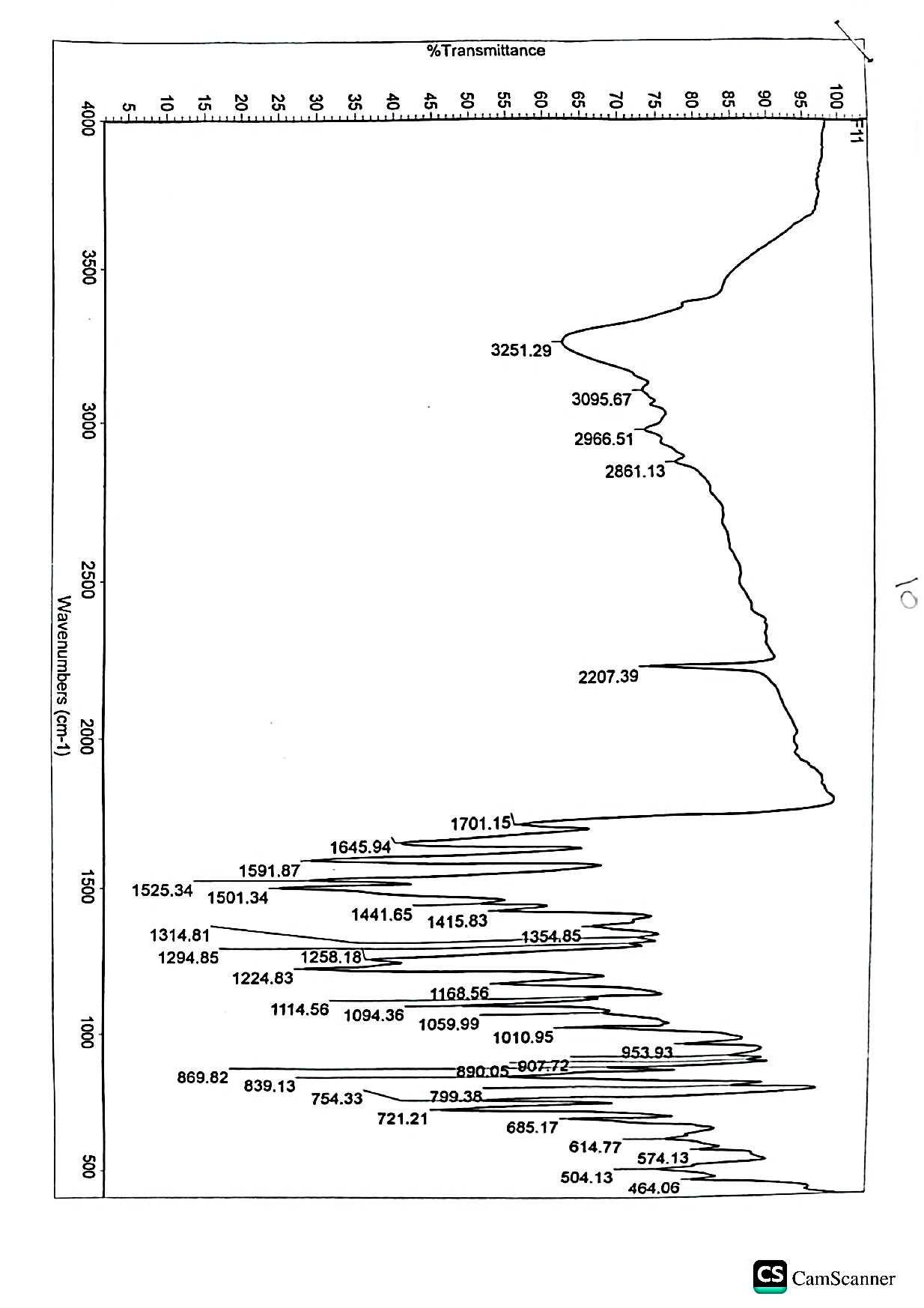


IR spectrum of compound 10


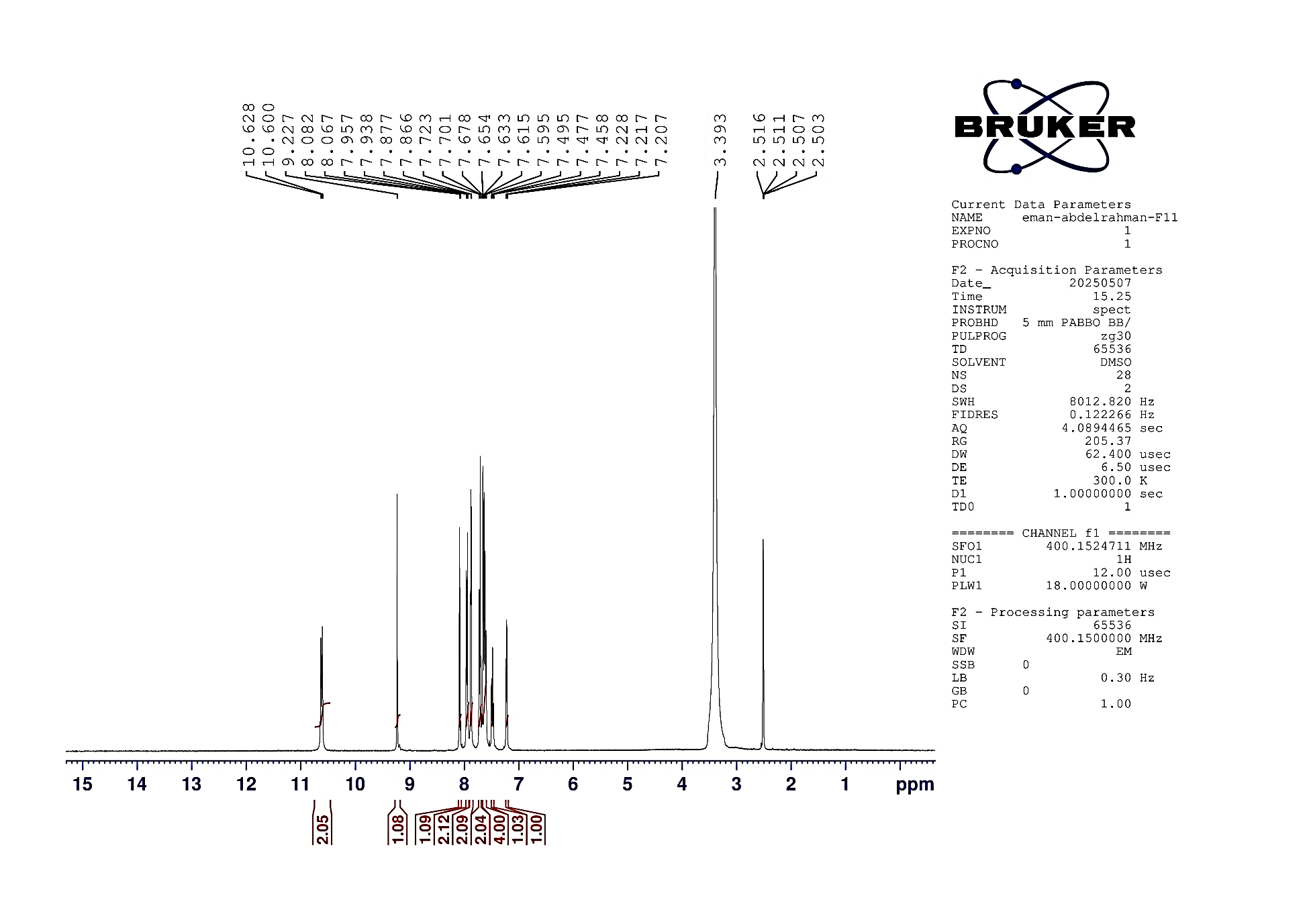


^1^H NMR spectrum (DMSO-*d*_6_) of compound 10


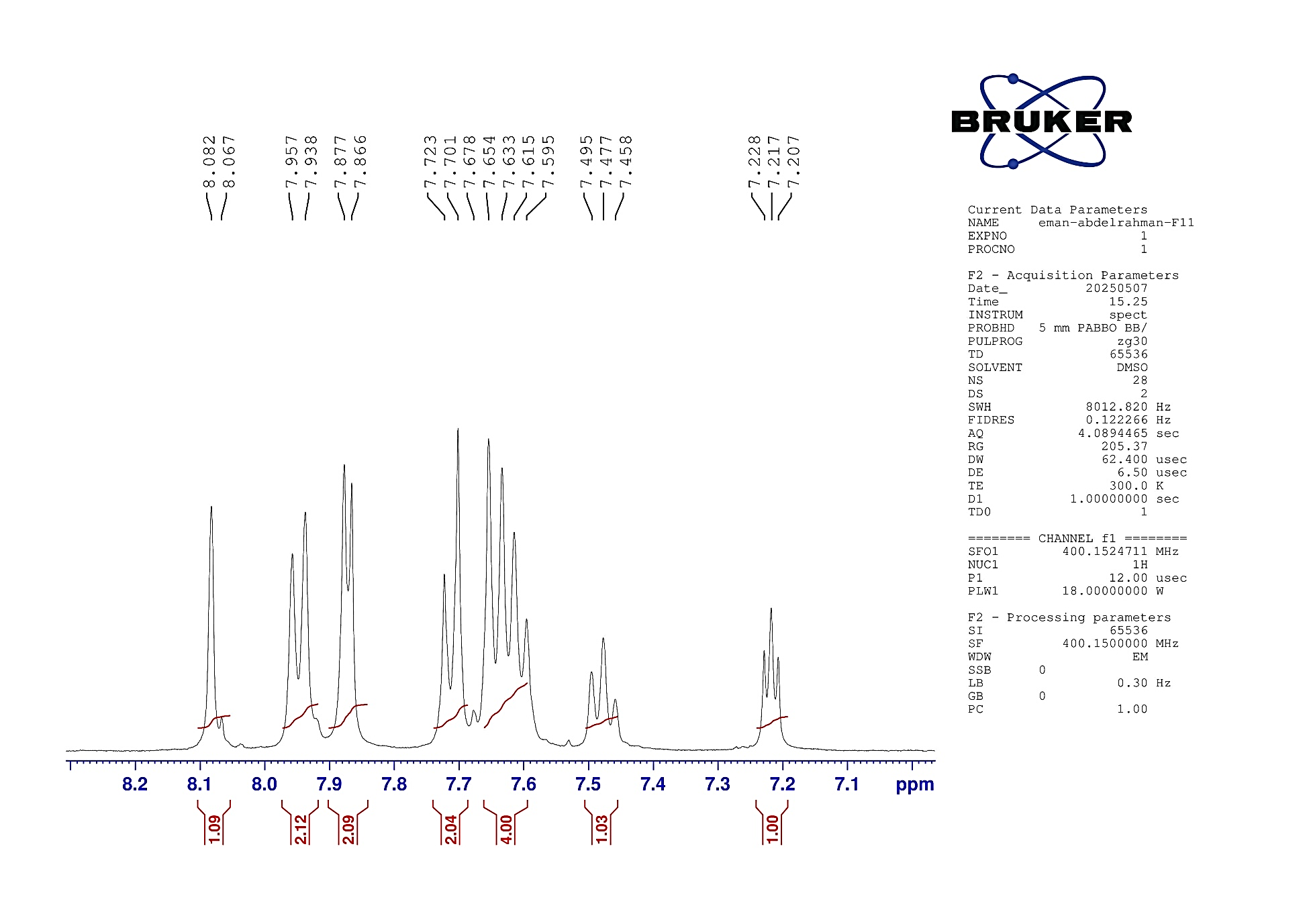


Cont. ^1^H NMR spectrum (DMSO-*d*_6_) of compound 10


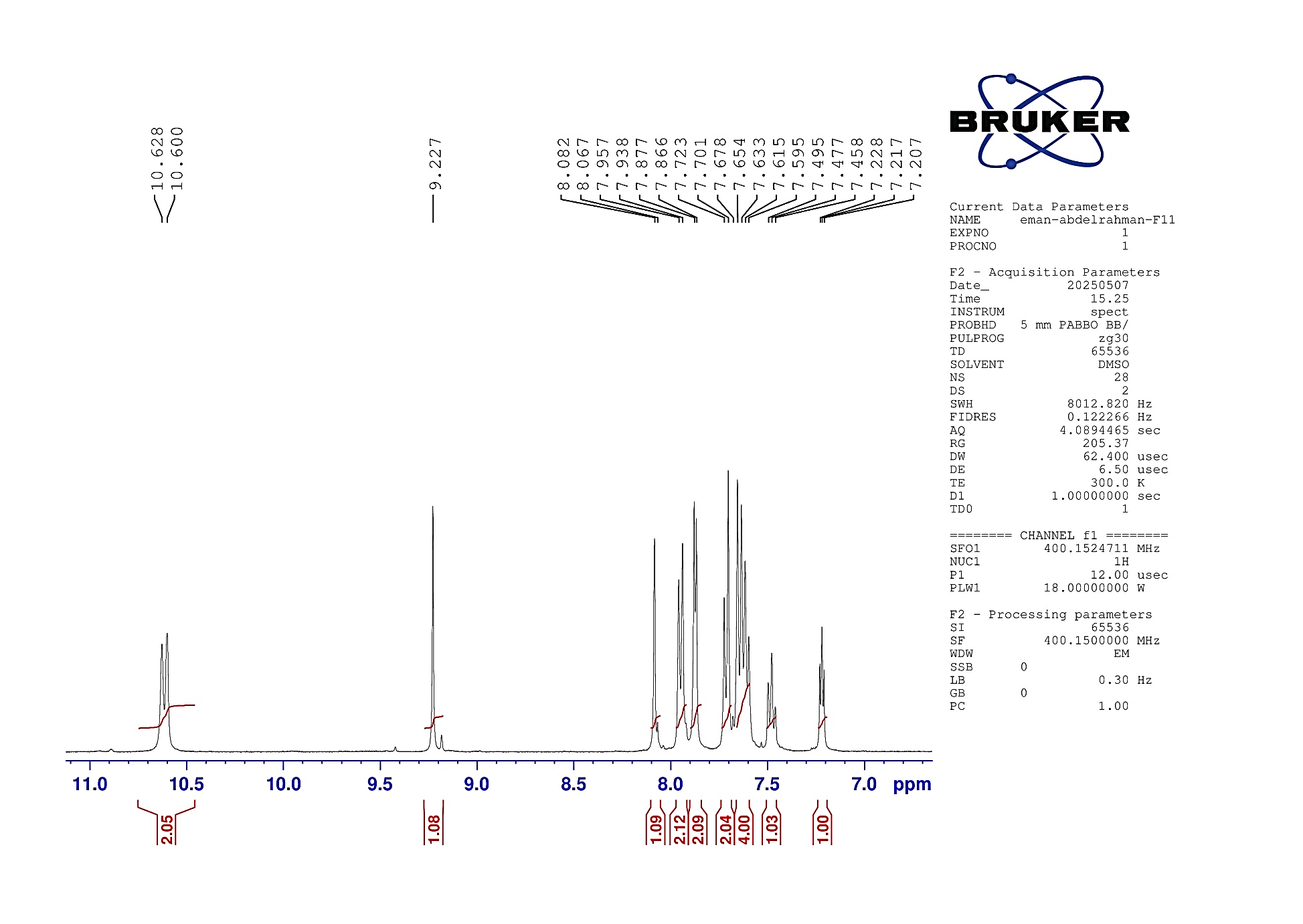


^1^H NMR spectrum (DMSO-*d*_6_) of compound 10


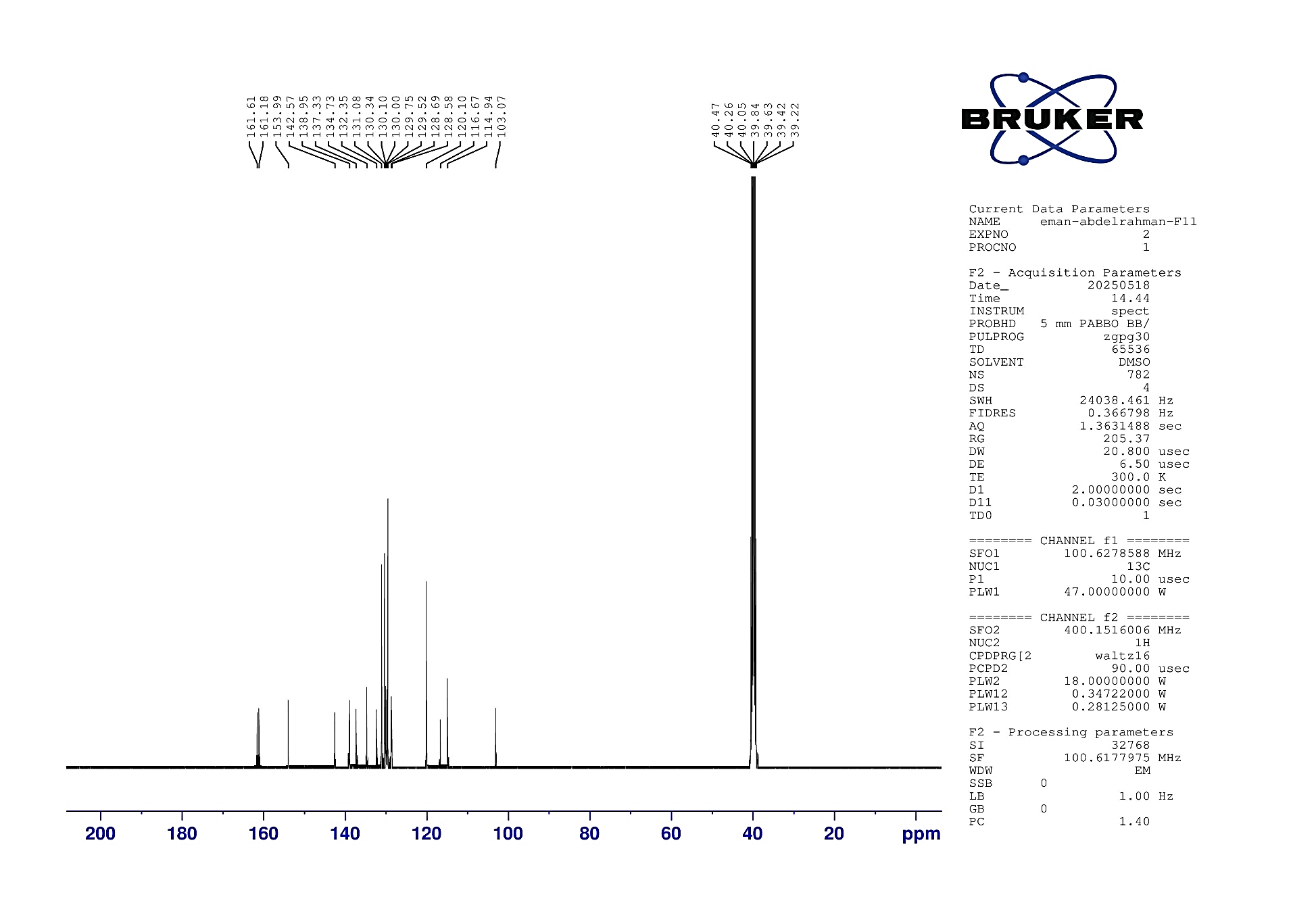


^13^C NMR spectrum (DMSO-*d*_6_) of compound 10


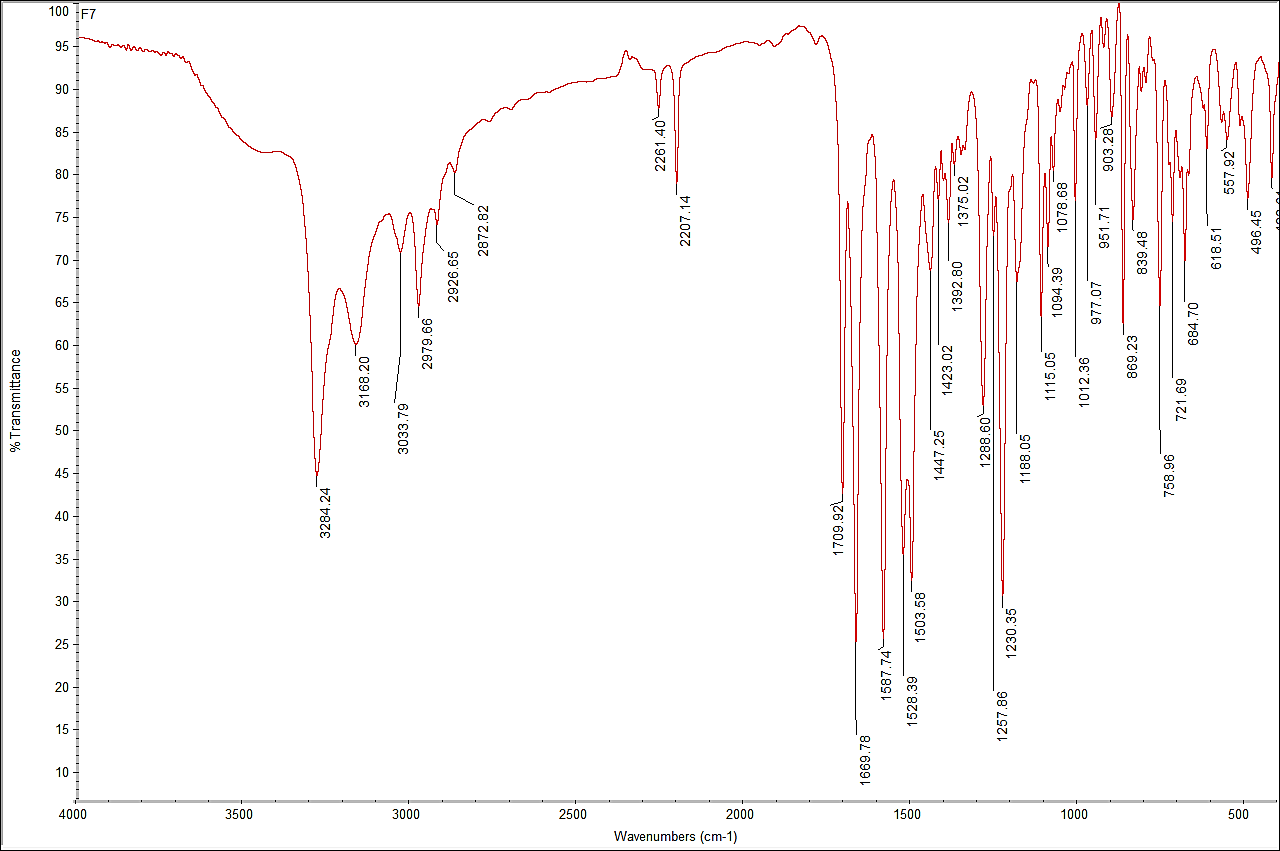


FT-IR spectrum of compound 11


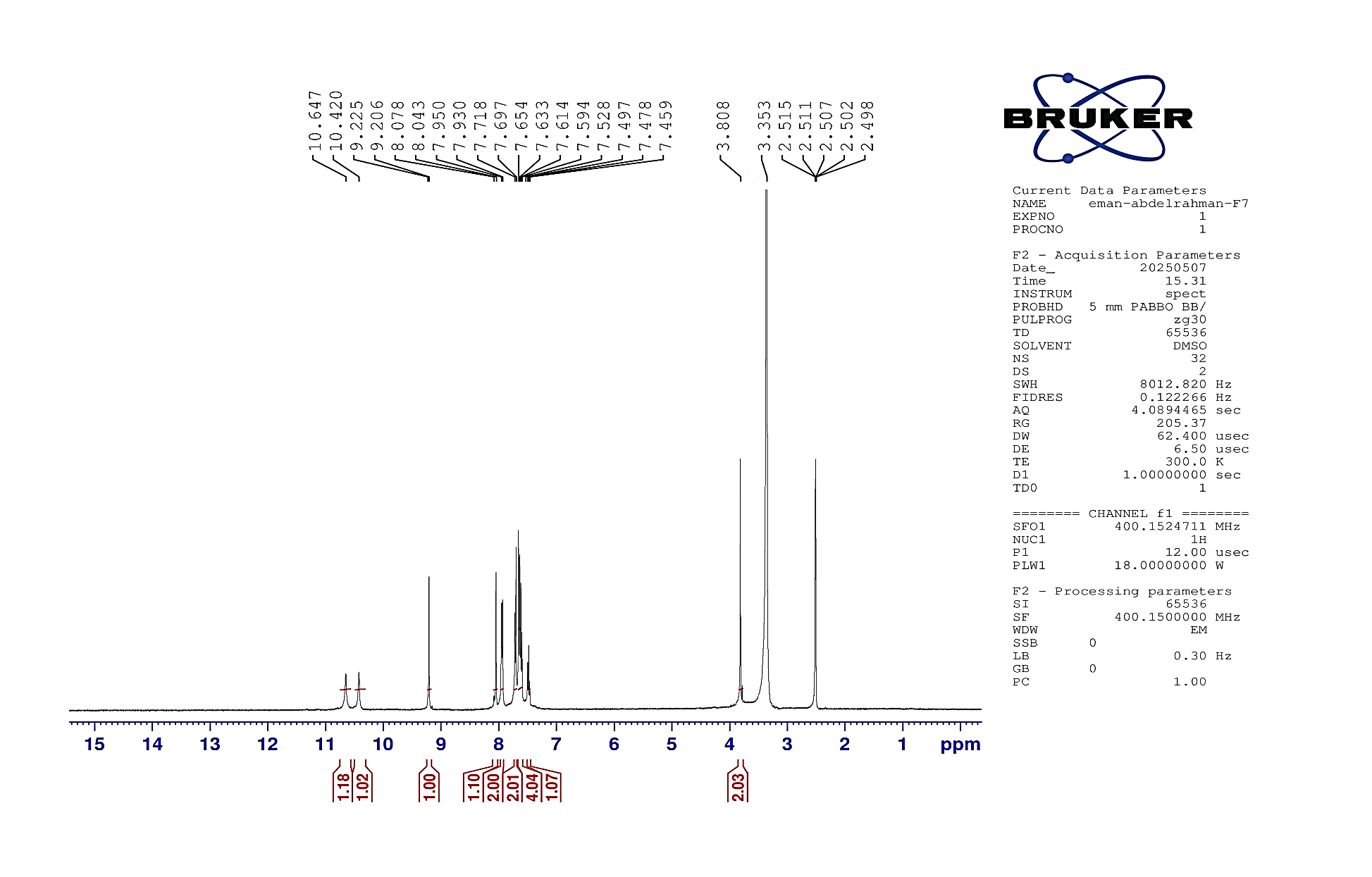


^1^H NMR spectrum (DMSO-*d*_6_) of compound 11


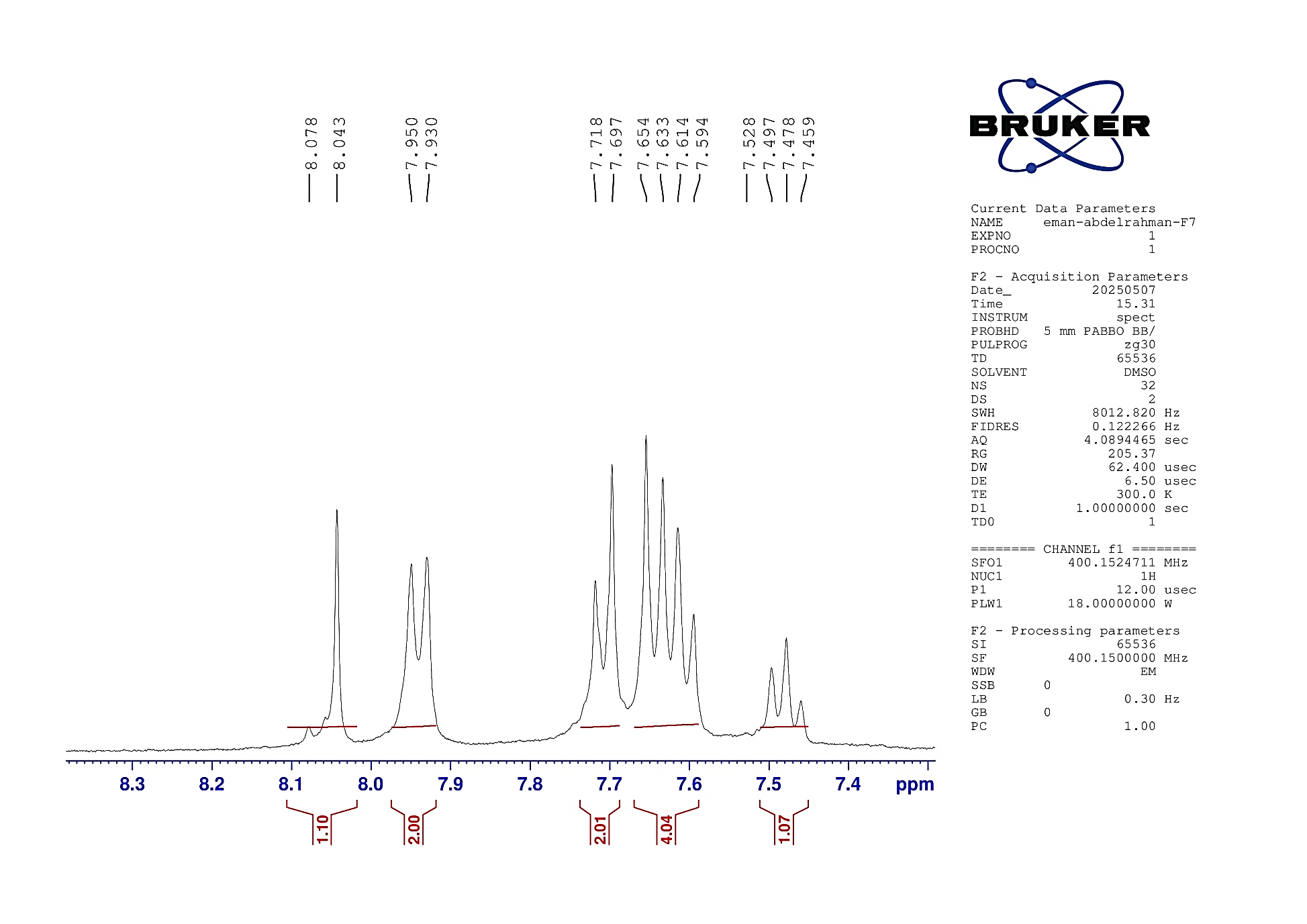


Cont. ^1^H NMR spectrum (DMSO-*d*_6_) of compound 11


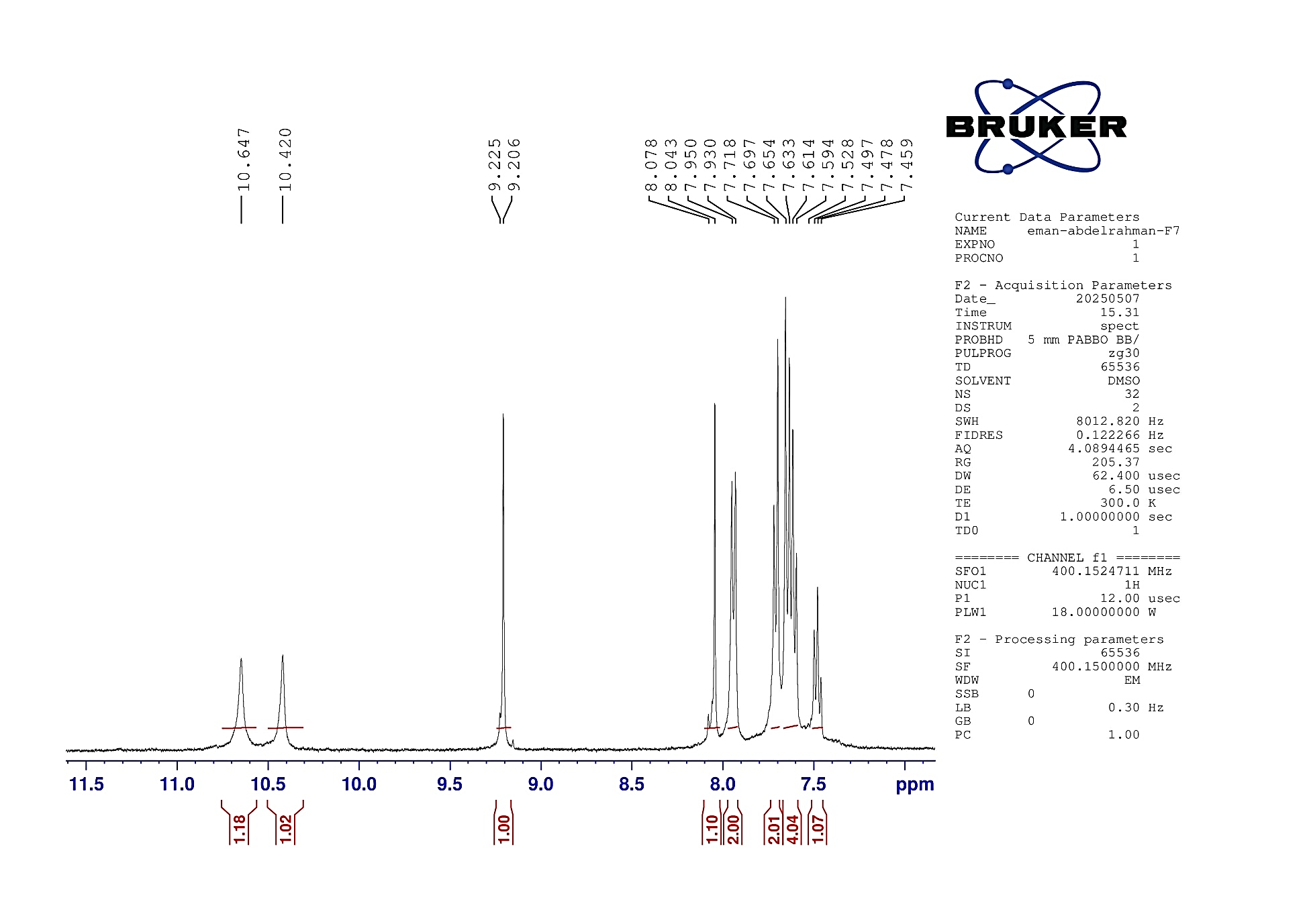


Cont. ^1^H NMR spectrum (DMSO-*d*_6_) of compound 11


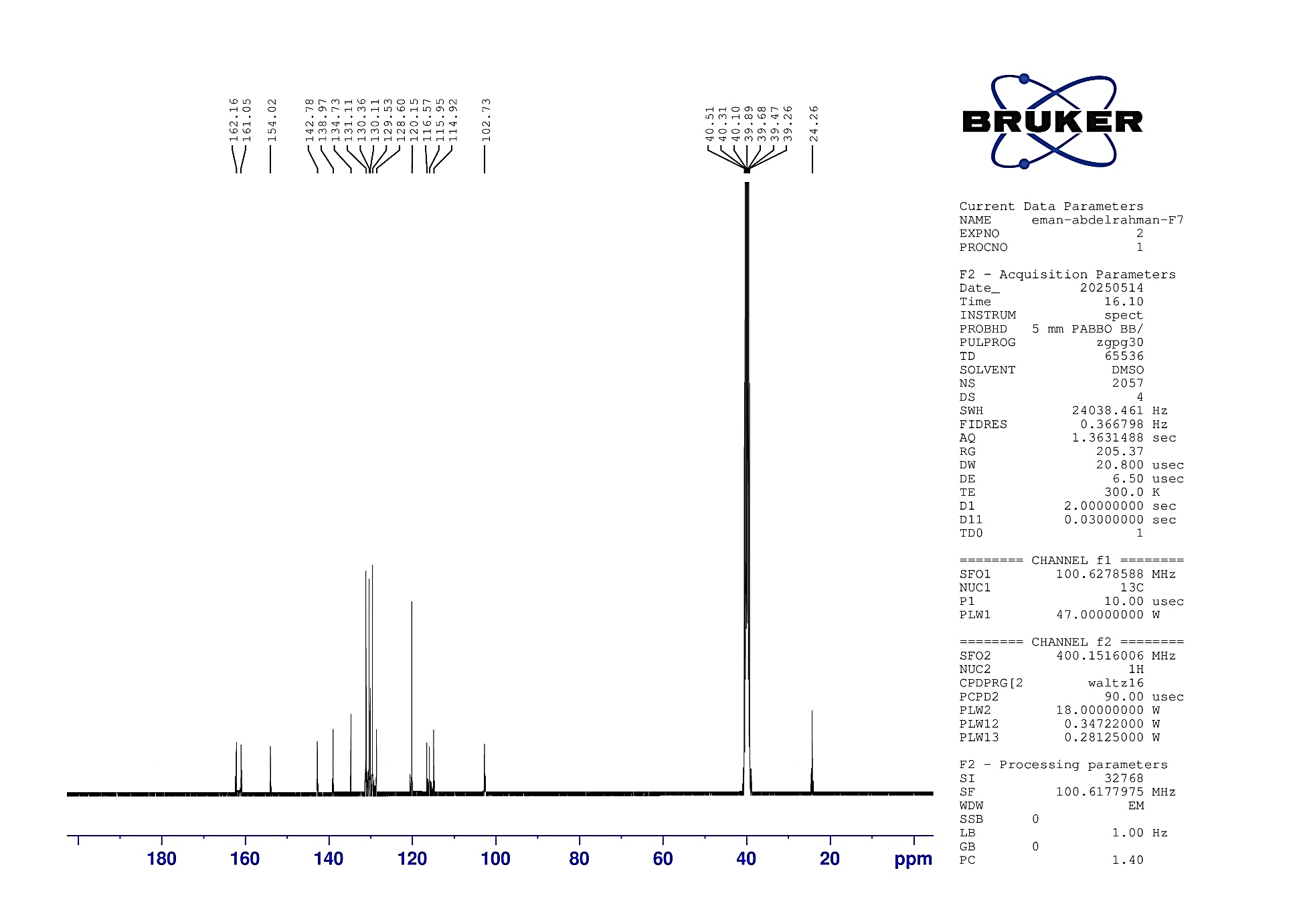


^13^C NMR spectrum (DMSO-*d*_6_) of compound 11


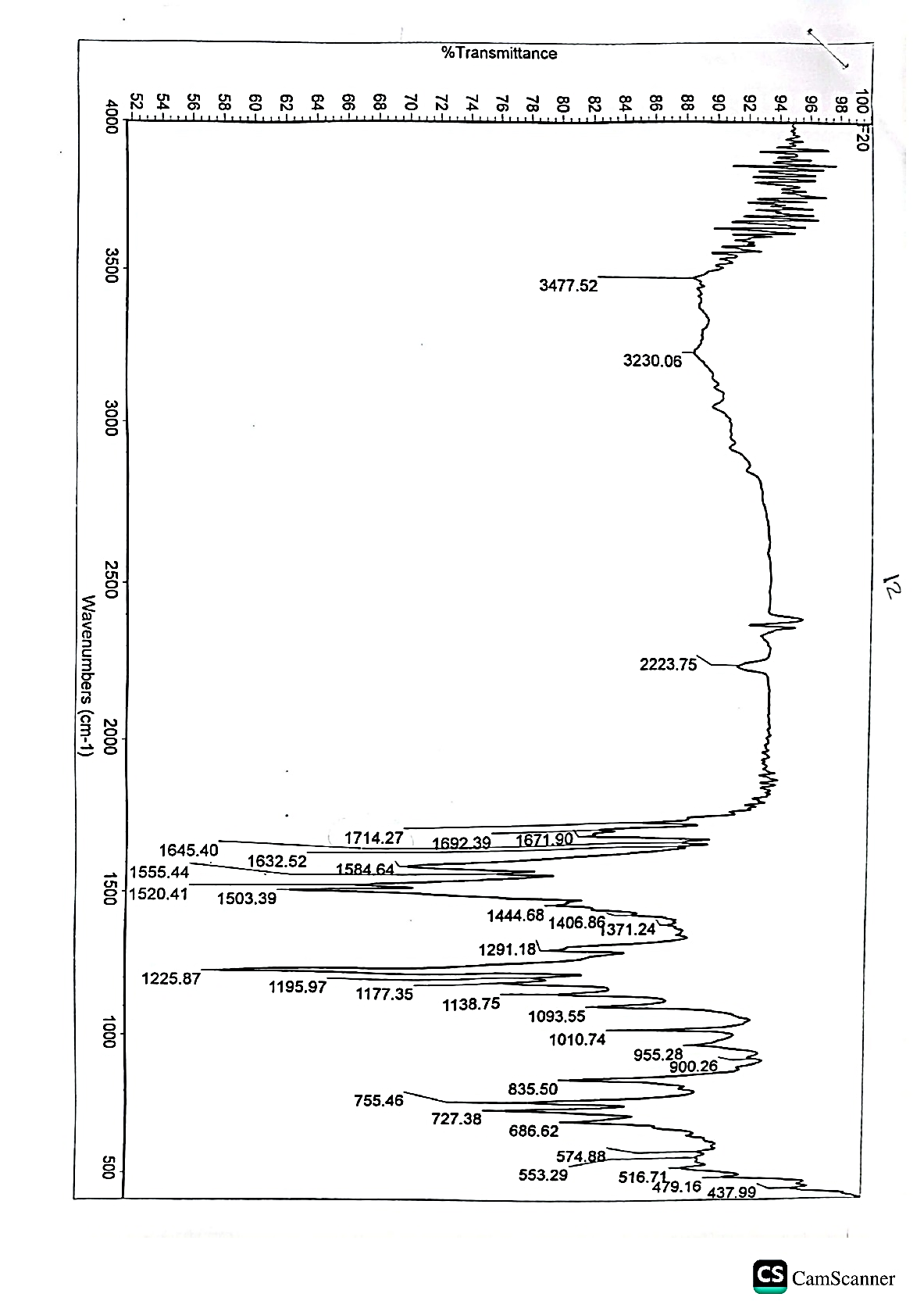


IR spectrum of compound 12


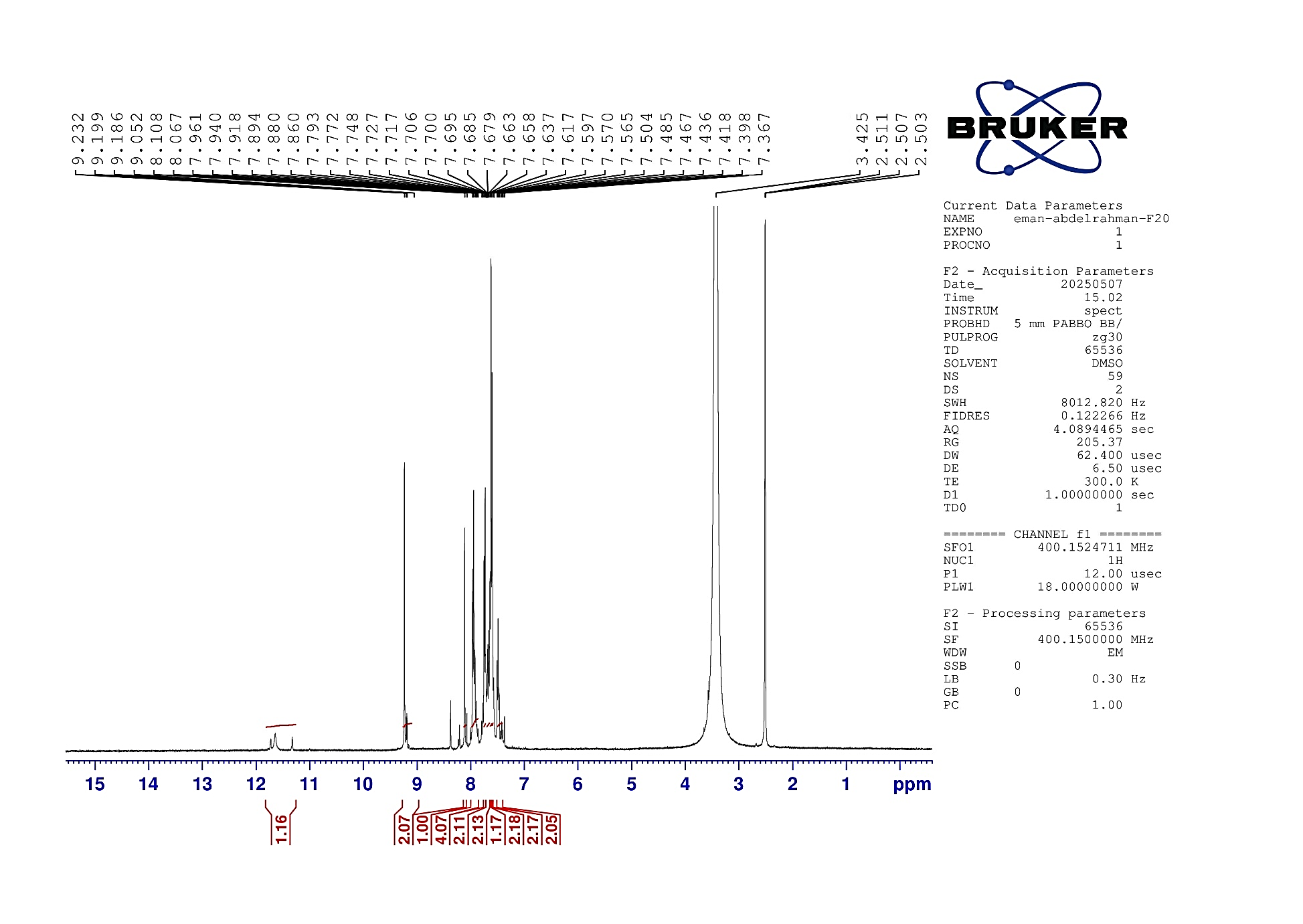


^1^H NMR spectrum (DMSO-*d*_6_) of compound 12
